# Multiscale Neural Network Potential with Anisotropic Message Passing for the Fast and Accurate Simulation of Protein Dynamics and Enzymatic Reactions

**DOI:** 10.1021/jacs.6c00217

**Published:** 2026-07-01

**Authors:** Moritz Thürlemann, Felix Pultar, Igor Gordiy, Enrico Ruijsenaars, Sereina Riniker

**Affiliations:** † Department of Chemistry and Applied Biosciences, 27219 ETH Zürich, Vladimir-Prelog-Weg 2, 8093 Zürich, Switzerland

## Abstract

We present the next generation of AMP, a neural network potential (NNP) with anisotropic message passing designed to study large biomolecular systems at DFT accuracy in the condensed phase using a multiscale approach similar to quantum-mechanics/molecular-mechanics (QM/MM) with electrostatic embedding. We trained AMPv3 on our recently published biomolecular multiscale simulation (BMS25) data set and demonstrated the model’s high efficiency, which enabled us to simulate proteins involving thousands of atoms at DFT accuracy in addition to explicit MM solvent for up to 100 ns, which presents a major leap for contemporary NNPs. We observe excellent scaling to large systems on a single GPU. AMPv3-BMS25 (or AMP-BMS for short) shows promising performance on benchmarks, and we demonstrate that the model can be used to accurately estimate experimental properties, including solvation free energies of small molecules and structural features of proteins. Finally, AMP-BMS/MM was employed to predict the free-energy profiles of reactions catalyzed by the enzymes chorismate mutase and fluoroacetate dehalogenase. In total, AMP-BMS/MM was used to simulate proteins in the condensed phase for a cumulative 23 μs simulation time or 48 billion integration steps. This work establishes AMP-BMS as a highly efficient and accurate model for multiscale simulations of biomolecules.

## Graphical abstract

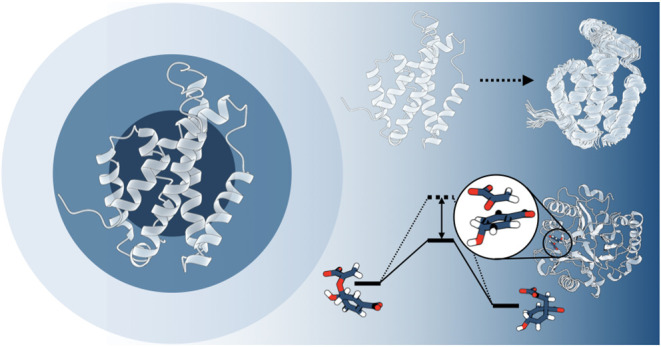

## Introduction

Accurate modeling of biological processes such as protein dynamics and enzymatic reactions has long been appreciated as one of the main objectives of computational chemistry.^1^ Molecular dynamics (MD) simulations provide insight into biological processes by connecting interactions at the atomic scale to macroscopic properties,^2–6^ with applications in the *in silico* design of pharmaceuticals^7–9^ or the development of enzymes with tailored catalytic functions and selectivities.^10–13^ Quantum mechanics (QM) provides the theoretical foundation underlying chemistry^14^ and led to the formulation of electronic structure methods such as Møller–Plesset perturbation theory^15^ and Kohn–Sham density functional theory (DFT).^16,17^ However, despite major advances in hardware capabilities^18^ and advances in electronic structure code (e.g., refs 19–22 ), it is currently not feasible to perform *ab initio* MD simulations of systems involving hundreds of thousands of atoms for extended periods of time due to the unfavorable scaling of these methods. Thus, classical force fields^23^ have been developed since the 1970s to study such extended systems using classical mechanics to describe interactions between atoms. This simplified description of atomic interactions offers favorable computational scaling with system size compared to QM methods, however, at the cost of accuracy and transferability. In a typical MD simulation of a protein solvated in water, interactions between water molecules account for the majority of interactions of the whole system. As a result, a significant portion of computational resources are allocated to water–water interactions, which are often of less interest. Therefore, early on, multiscale approaches like quantum-mechanics/molecular-mechanics (QM/MM)^24–27^ have been proposed, in which the system is split into a QM zone and a MM zone. By following this formalism, the higher accuracy of QM methods can be retained for the region of interest (QM zone), while exploiting the better scaling of classical force fields for the environment (MM zone). Such schemes reduce the computational cost dramatically compared to *ab initio* MD, however, QM/MM schemes with DFT for the QM zone are still too expensive for long time scale simulations of proteins in solution.

Neural network potentials (NNPs) have emerged as a potential solution to this trade-off between accuracy and computational efficiency^28,29^ and have been suggested to serve as cheap proxies for expensive QM Hamiltonians when trained on corresponding reference data. Recent years have seen significant efforts in developing the theory and architectures of the underlying machine learning (ML) models.^30–45^ As the field has matured, efforts have increasingly shifted toward enabling practical applications by integrating NNPs with existing MD and multiscale simulation packages and developing user-friendly interfaces for the broader community.^46–54^

MD simulations involving NNPs fall into two categories. In the first category, the entire system is described by a NNP similar to *ab initio* MD.^55–59^ While much cheaper than DFT, simulations with NNPs in this category are still limited by computational cost and memory requirements regarding sampling times and applications to large systems. Multi-GPU setups are needed to access larger systems, see e.g., ref 39. Therefore, overly short graph cutoffs are often applied, which can limit accuracy in condensed-phase systems due to the importance of long-range interactions.^60^ Attempts to address these limitations involve the addition of long-range interaction terms.^36,61–65^

In the second category, the system is partitioned analogously to QM/MM schemes into a region of high accuracy evaluated by the NNP and a region of reduced accuracy described by a classical force field.^66^ Following the similarity to QM/MM, such schemes are often termed ML/MM and allow simulations of much larger systems at reduced computational cost.^46,51,67–80^ For these designs to be successful, the coupling between the different subsystems is of utmost importance. Currently, most approaches in this category rely on a mechanical-embedding scheme^46,67,68,72–74,77,81^ or approximated electrostatic embedding.^82,83^ Although computationally efficient and easy to implement with NNPs, mechanical embedding is known to suffer from the lack of polarization of the QM zone by the MM zone.^25,27,84,85^ To avoid these limitations, we and others have developed ML/MM schemes that use electrostatic embedding directly instead.^51,69–71,76,86–90^ In 2021, we published a first approach employing a high-dimensional NNP (HDNNP),^63,91,92^ which was trained for each system separately. In 2023, we published the anisotropic message passing (AMP) architecture^86^ designed for ML/MM simulations. The second version of the AMP architecture (AMPv2) was used to propagate MD simulations of small peptides and to estimate dissociation free energies of challenging molecular systems including charged species and transition metals.^51^ Again, an AMP model was trained for each system and we explored the transferability of the model to similar molecules. Encouraged by the promising performance of AMPv2, we set out to develop a foundational (or global) AMP model that can be applied to simulate proteins and even enzymatic reactions featuring tens of thousands of atoms. In this work, we introduce the third version of the AMP architecture (AMPv3, Figure 1), which is trained on QM/MM reference data of approximately 60’000 molecular systems with 1.5 million conformations of small molecules and peptides including explicit solvation (BMS25 data set^93^) to provide a generally applicable NNP for electrostatic-embedding ML/MM MD simulations of biomolecular condensed-phase systems. For the naming convention of the AMP models, we propose the format: [AMP architecture]-[embedding scheme]-[training set]. The presented model is thus AMPv3-EE-BMS25. For simplification, we will use the shorthand notation AMP-BMS in the following. In addition to 100 ns simulations of proteins in water, this work also demonstrates the use of AMP-BMS for the description of enzyme-catalyzed reactions in water, which is an important application of ML/MM schemes.

**Figure 1:**
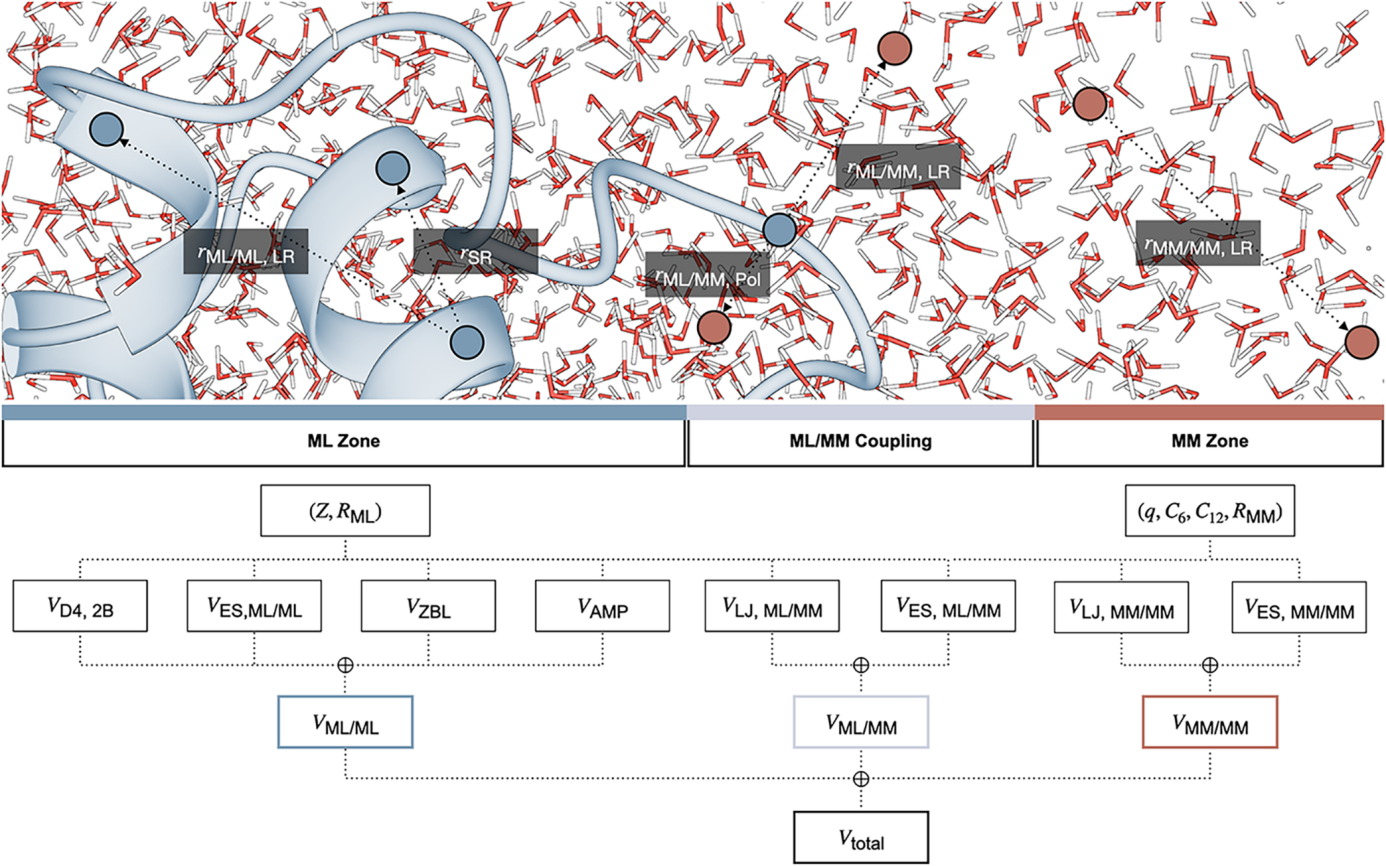
Summary of the architecture of the AMPv3 model for ML/MM simulations with electrostatic embedding. The different terms are described in the Theory section.

## Theory

The model described in this work is the third version of the anisotropic message passing (AMP) architecture proposed in previous work^86^ combined with physics-based terms such as the shifted reaction-field method^94^ for description of the long-range electrostatics and D4 ^95–97^ to account for dispersion. The AMP model was developed as an *SO*(3)-equivariant message-passing architecture for the description of ML/MM systems within an electrostatic embedding formalism. The concept has since been expanded and applied to a variety of systems.^51^ In this work, the AMP architecture was modified to allow more efficient scaling to larger systems with increased accuracy. These modifications and an overall description are given in the following sections.

Essential to the AMP architecture and the QM/MM formalism is a separation into subsystems and the coupling between them. For the present work, all protein and ligand atoms are assigned to the ML subsystem and the solvent molecules are assigned to the MM subsystem. The total potential energy can thus be expressed as

1 $$V_{\mathrm{total}}=V_{\mathrm{ML}/\mathrm{ML}}+V_{\mathrm{ML}/\mathrm{MM}}+V_{\mathrm{MM}/\mathrm{MM}}$$

where *V*_ML/ML_ denotes the potential energy between particles in the ML zone and *V*_ML/MM_ the interaction between the ML and MM zone. Interactions between particles in the MM zone, *V*_MM/MM_, are described by a classical force field. The most important feature distinguishing the AMP formalism from other NNP architectures is the coupling between ML and MM particles, which is described below.

### Interactions Within the ML Zone (*V*_ML/ML_)

Interactions between particles in the ML subsystem are decomposed into four components:

2 $$V_{\mathrm{ML}/\mathrm{ML}}=V_{\mathrm{AMP}}+V_{D4,2B}+V_{\mathrm{ES},\mathrm{ML}/\mathrm{ML}}+V_{\mathrm{ZBL}}$$

where *V*_AMP_ denotes the sum of atomic contributions to the potential energy, *V*_D4,2B_ is a dispersion correction according to the two-body component in the D4 model, *V*_ES,ML/ML_ describes the long-range electrostatics modeled by the shifted reaction field method,^94^ and *V*_ZBL_ is a short-range repulsion potential modeled by the Ziegler-Biersack-Littmark (ZBL) stopping potential.^98^

#### Definition of *V*_AMP_

The AMP model builds atomic multipoles **M**_*i*_^*k*^ of order *k* on each atom *i*, which are then used to incorporate directional information in subsequent message passing steps. Given the unit vector *r⃗*_*ij*_ pointing from node *i* to *j*, each multipole is constructed from a linear combination of local bases **R**_*ij*_^*k*^ formed by a tensor product:

3 $$R_{\mathrm{ij}}^{k}=\underset{k\mathrm{times}}{\underset{︸}{{\mathrm{r⃗}}_{\mathrm{ij}}⊗{\mathrm{r⃗}}_{\mathrm{ij}}⊗...}}$$

with **R**_*ij*_^0^ = 1 and *k* referring to the multipole order. Each multipole of order *k* is then expressed as

4 $$M_{i}^{k}=\sum_{j\in N\left(i\right)}c_{\mathrm{ij}}^{k}R_{\mathrm{ij}}^{k}$$

where *N*(*i*) denotes the set of neighboring atoms of atom *i*. The scalar coefficients *c*_*ij*_^*k*^ are predicted for each interaction as

5 $$c_{\mathrm{ij}}^{k}=\phi_{M\left(k\right)}\left(h_{i},h_{j},u_{\mathrm{ij}}\right)$$

from its edge feature *u*_*ij*_ and the hidden node features *h*_*i*_ and *h*_*j*_. ϕ_*M*(*k*)_ refers to a neural network parametrized learnable function. Edge features are composed of Bessel function embedded distances *b*(*r*_*ij*_) and the one-hot encoded vectors of the interacting elements using a single linear layer.^35^ Edges are introduced for all atom pairs within a short-range cutoff *r*_SR_.

For each message passing interaction, the multipoles **M**_*i*_^*k*^ are used to construct the anisotropic feature *g*_*ij*_. The components for a model of order *k* = 2 are given as follows:^99^

6 $$\begin{array}{l} g_{0}=M_{i,\alpha }^{1}R_{\mathrm{ij},\alpha }^{1}\\ g_{1}=M_{j,\alpha }^{1}R_{\mathrm{ij},\alpha }^{1}\\ g_{2}=M_{i,\alpha }^{1}M_{j,\alpha }^{1}\\ g_{3}=M_{i,\alpha \beta }^{2}R_{\mathrm{ij},\alpha \beta }^{2}\\ g_{4}=M_{j,\alpha \beta }^{2}R_{\mathrm{ij},\alpha \beta }^{2}\\ g_{5}={\left(M_{i,\alpha \beta }^{2}R_{\mathrm{ij},\beta }^{1}\right)}_{\alpha }M_{j,\alpha }^{1}\\ g_{6}={\left(M_{j,\alpha \beta }^{2}R_{\mathrm{ij},\beta }^{1}\right)}_{\alpha }M_{i,\alpha }^{1}\\ g_{7}=M_{i,\alpha \beta }^{2}M_{j,\alpha \beta }^{2}\\ g_{8}={\left(M_{i,\alpha \beta }^{2}R_{\mathrm{ij},\beta }^{1}\right)}_{\alpha }{\left(M_{j,\alpha \beta }^{2}R_{\mathrm{ij},\beta }^{1}\right)}_{\alpha } \end{array}$$

Contractions are performed over the Greek indices. These components have been simplified compared to the initial publication of the AMP model^86^ and no longer include products of scalars, such as the monopole-monopole term. Each message feature is thus composed of the hidden features of the interacting nodes, edge features *u*_*ij*_, and the multipole interaction components *g*_*ij*_. In summary, the modified message passing is defined as

7 $$\begin{array}{l} M_{i}^{k}=\sum_{j\in N\left(i\right)}\phi_{M\left(k\right)}\left(h_{i},h_{j},u_{\mathrm{ij}}\right)R_{\mathrm{ij}}^{k}\\ h_{i}^{l+1}=h_{i}^{l}+\phi_{h}\left(h_{i}^{l},\sum_{j\in N\left(i\right)}\phi_{e}\left(h_{i}^{l},h_{j}^{l},u_{\mathrm{ij}},g_{\mathrm{ij}}\right)\right) \end{array}$$

During each message passing step, multipoles **M**_*i*_^*k*^ are first expanded on each atom followed by the calculation of the anisotropic features *g*_*ij*_ for each pair of nodes *h* connected by an edge with an edge-feature *u*(*r*_*ij*_) embedding the distance between two nodes. Finally, hidden features *h* ^*l*^ are updated to *h* ^*l*+1^ using a learnable function ϕ_*h*_ and skip connections from *h* ^*l*^. The potential energy is then expressed as a sum over contributions of each node using a neural network-parametrized potential ϕ_*V*_:

8 $$V_{\mathrm{AMP}}=\sum_{i}^{N_{\mathrm{ML}}}\phi_{V}\left(h_{i}\right)$$

Compared to the first AMP version in ref 86, several changes have been made to the AMP architecture to reduce computational cost and improve accuracy. In addition to the simplification of the multipole interaction coefficients *g*_*ij*_ and small modifications of the message passing scheme already described, the most important change concerns the dimensionality of the multipole tensor. Instead of working with a single set of multipoles, several sets of multipoles are expanded on each atom. While only one set of multipoles bears physical meaning and is used in calculations involving electrostatic interactions (see below), this change was made to improve the resolution of directional information using the anisotropic message passing scheme.

#### Definition of *V*_D4,2B_

The description of the long-range dispersion interactions within the ML subsystem follows the same treatment proposed in SpookyNet,^36^ i.e., using the two-body component of the D4 dispersion model.^95–97^ As the accurate description of the molecular dipole is essential for the interaction between the ML and the MM particles, *C* ^6^ coefficients are not obtained from the same partial charges that are used to calculate the electrostatic interaction within the ML subsystems and between the ML and MM subsystems (see below), but instead, *C* ^6^ coefficients are estimated as the product of the *C* ^6^ coefficient of the respective element *in vacuo C*_*i*,ref_^6^ and a scaling factor:

9 $$C_{i}^{6}=\phi_{C^{6}}\left(h_{i}\right)\cdot C_{i,\mathrm{ref}}^{6}$$

with the scaling factor predicted by a neural network-parametrized function ϕ_*C* ^6^_ given the hidden features of atom *i*. Pairwise *C* ^6^ coefficients are obtained as

10 $$C_{\mathrm{ij}}^{6}=\sqrt{C_{i}^{6}\cdot C_{j}^{6}}$$

and then used to obtain the pairwise D4 dispersion term:

11 $$V_{D4,2B}=-\sum_{i>j}^{N_{\mathrm{ML}}}\sum_{n=6,8}s_{n}\frac{C_{\mathrm{ij}}^{n}}{r_{\mathrm{ij}}^{n}}f_{\mathrm{BJ}}^{n}\left(r_{\mathrm{ij}}\right)$$

using the Becke-Johnson damping function *f*_*BJ*_^*n*^(*r*_*ij*_).^100,101^ Damping- and scaling-coefficients of the reference method (ωB97M) are used without any modifications (*s*_6_ = 1.0, *s*_8_ = 0.7761, *a*_1_ = 0.7514, *a*_2_ = 2.7099).^96^ The dispersion interaction term is applied to all pairs of ML particles within the long-range cutoff, i.e., *r*_*ij*_ < *r*_ML/ML,LR_.

#### Definition of *V*_ES,ML/ML_

In addition to the dispersion interaction, long-range electrostatic interactions were included using

12 $$V_{\mathrm{ES},\mathrm{ML}/\mathrm{ML}}=\sum_{i>j}^{N_{\mathrm{ML}}}\left(1-f_{\mathrm{switch}}\left(r_{\mathrm{ij}}\right)\right)\cdot u_{\mathrm{RF}}\left(r_{\mathrm{ij}}\right)\frac{q_{i}q_{j}}{4\pi \epsilon_{0}}$$

with monopoles *q*, *u*_RF_(*r*_*ij*_) referring to the shifted reaction-field proposed by Kubincová et al.^94,102^ and *f*_switch_(*r*_*ij*_) to a switching function:^103^

13 $$\begin{array}{l} f_{\mathrm{switch}}\left(x\right)=1-6x^{5}+15x^{4}-10x^{3},\\ x\left(r\right)=\frac{\left(r-r_{\mathrm{switch}}\right)}{(r_{\mathrm{SR}}-r_{\mathrm{switch})}} \end{array}$$

We set *r*_switch_ = 0 Å and *f*_switch_(*x*) is clipped to 0 for *x* > 1, i.e., full reaction-field electrostatics were applied to all pairs of ML particles with *r*_SR_ ≤ *r*_*ij*_ < *r*_ML/ML,LR_. Electrostatic interactions within the ML subsystem were only computed using monopoles assuming that short-ranged higher-order contributions are captured by the ML potential. Default exponents for the reaction field were used (*m* = 4 and *n* = 6)^94^ and the dielectric permittivity was set to that of water ϵ = 78.4.^104^

14 $$M_{i}^{0}=\phi_{\mathrm{mono}}\left(h_{i}^{l}\right)-\frac{1}{N_{\mathrm{ML}}}\sum_{j=1}^{N_{\mathrm{ML}}}\phi_{\mathrm{mono}}\left(h_{j}^{l}\right)-Q_{\mathrm{ML}}$$

The monopoles (one per node) used for long-range electrostatic-energy calculations are predicted by the learnable function ϕ_mono_ from the final node embedding *h*_*i*_^*l*^ after all ML-ML and ML-MM message-passing steps are done as defined in eq 14. The model receives the total molecular charge *Q*_ML_ as an input and guarantees charge conservation by distributing the residual charge over the whole molecule.

#### Definition of *V*_ZBL_

Following earlier work,^36^ a short-range repulsion is included on the basis of the ZBL stopping potential:^98^

15 $$V_{\mathrm{ZBL}}=\frac{1}{4\pi \epsilon_{0}}\sum_{\begin{array}{l} i<j\\ r_{\mathrm{ij}}<r_{\mathrm{SR}} \end{array}}^{N_{\mathrm{ML}}}\frac{Z_{i}Z_{j}}{r_{\mathrm{ij}}}f_{\mathrm{cut}}\left(r_{\mathrm{ij}}\right)\left(\sum_{k=1}^{4}c_{k}\exp\left(-a_{k}r_{\mathrm{ij}}\left(Z_{i}^{p}+Z_{j}^{p}\right)/d\right)\right)$$

where *a*_*k*_, *c*_*k*_, *p*, and *d* are the parameters given by ref 98, which were not modified during training. *f*_cut_ is the switching function of the form:

16 $$f_{\mathrm{cut}}\left(r_{\mathrm{ij}}\right)=\{\begin{array}{ll} 1 & r_{\mathrm{ij}}\leq r_{\mathrm{SR}}-1\\ \mathrm{clip}\left(1-6x^{5}+15x^{4}-10x^{3},0,1\right) & r_{\mathrm{SR}}-1<r_{\mathrm{ij}}<r_{\mathrm{SR}}\\ 0 & r_{\mathrm{ij}}\geq r_{\mathrm{SR}} \end{array}$$

where *x*(*r*_*ij*_) = *r*_*ij*_ – (*r*_SR_ – 1). This term is included for all edges, i.e., *r*_*ij*_ < *r*_SR_. We found that this potential is in principle not necessary but helpful in cases where an initial geometry includes clashes.

### Interactions Between the ML and MM Zones (*V*_ML/MM_)

One of the defining features of the AMP architecture is the ability to describe the polarization introduced by a surrounding charge distribution efficiently. The ML/MM interaction is split into two components, electrostatic interaction and the Lennard-Jones (LJ) term:

17 $$V_{\mathrm{ML}/\mathrm{MM}}=V_{\mathrm{ES},\mathrm{ML}/\mathrm{MM}}+V_{\mathrm{LJ},\mathrm{ML}/\mathrm{MM}}$$

To account for the polarization of the ML zone by the MM partial charges, an additional set of multipoles is expanded during the last step of message passing:

18 $$M_{i,\mathrm{ML}/\mathrm{MM}}^{k}=\lambda_{\mathrm{pol}}\sum_{j}^{N_{\mathrm{MM}}}\phi_{\alpha_{i}^{k}}\left(h_{i}\right)\phi_{b}\left(u_{\mathrm{ij}}^{*}\right)R_{\mathrm{ij}}^{k}\frac{M_{j}^{0}}{r_{\mathrm{ij}}^{2}}$$

where *u*_ij_^*^ is the ML-MM edge embedding. In other words, the contribution to the polarization of the ML zone by the MM zone is described as the product of a polarizability α_*i*_^*k*^, the electric field caused by the MM particles, a distance dependent contribution *b*_*ij*_, and a constant damping factor λ_pol_, which accounts for overpolarization (see below). These contributions are only included for dipoles and quadrupoles, i.e., for *k* > 0. The polarizability α_*i*_^*k*^ is predicted for each node in the ML zone by a neural network. Similarly, the distance-dependent factor ϕ_*b*_(*u*_*ij*_) is predicted for each pair of ML and MM particles given the Bessel function embedded radial features. This factor accounts for damping effects at short ranges.^105^ The resulting multipoles are then concatenated with the other multipoles expanded during message passing to introduce the coupling between the ML and MM zone. Only MM particles within a cutoff *r*_ML/MM,Pol_ of a ML particle contribute to its polarization. In the charge scaling scheme (see below), charges within the MM zone are scaled by a constant factor λ_ch_

19 $${\mathrm{M̂}}_{j}^{0}=\lambda_{\mathrm{ch}}M_{j}^{0}\forall j\in N_{\mathrm{MM}}$$

thus damping both polarization and electrostatic interactions. The electrostatic interaction is described as an interaction between atomic multipoles centered on each atom in the ML zone (same monopoles as defined in eq 14) and the partial charges of the MM zone:

20 $$V_{\mathrm{ES},\mathrm{ML}/\mathrm{MM}}=\frac{1}{4\pi \epsilon_{0}}\sum_{l}^{2}\sum_{i}^{N_{\mathrm{ML}}}\sum_{j}^{N_{\mathrm{MM}}}B^{l}\left(r_{\mathrm{ij}}\right)G^{l}\left(R_{\mathrm{ij}}\right)$$

with indices *i* iterating over particles in the ML zone and *j* over particles in the MM zone. The superscript *l* refers to the order, which includes multipoles up to quadrupoles in the present work. The radial functions *B*(*r*_*ij*_) are given by

21 $$B^{l}\left(r_{\mathrm{ij}}\right)=\frac{\left(2l-1\right)!!}{r^{2l+1}}$$

with !! denoting the double factorial. The multipole interaction coefficients *G* ^*l*^(**R**_*ij*_) are given as

22 $$\begin{array}{l} G^{{}^{\circ}}\left(R_{\mathrm{ij}}\right)=M_{i}^{0}M_{j}^{0}\\ G^{1}\left(R_{\mathrm{ij}}\right)=\left(M_{i,\alpha }^{1}R_{\mathrm{ij},\alpha }^{1}\right)M_{j}^{0}\\ G^{2}\left(R_{\mathrm{ij}}\right)=\left(M_{i,\alpha \beta }^{2}R_{\mathrm{ij},\alpha \beta }^{2}\right)M_{j}^{0} \end{array}$$

where the tensor contractions are performed over the Greek indices, *G*_0_ denotes the interaction between two monopoles, *G*_1_ the interaction between the partial charge and the dipole, and *G*_2_ between the partial charge and the quadrupole.

Finally, the LJ interaction is computed as

23 $$V_{\mathrm{LJ},\mathrm{ML}/\mathrm{MM}}=\sum_{i}^{N_{\mathrm{ML}}}\sum_{j}^{N_{\mathrm{MM}}}\left(\frac{C_{\mathrm{ij}}^{12}}{r_{\mathrm{ij}}^{12}}-\frac{C_{\mathrm{ij}}^{6}}{r_{\mathrm{ij}}^{6}}\right)$$

with LJ parameters *C* ^6^ and *C* ^12^ (taken from a classical force field) to account for exchange-repulsion and dispersion interactions between the ML and MM zone. Only MM particles within *r*_ML/MM,LR_ of any ML particle contribute to these LJ interactions.

## Results and Discussion

We trained the AMPv3 architecture on the BMS25 data set,^93^ which consists of approximately 60’000 unique molecular systems with 1.5 million electrostatic embedding QM/MM reference calculations of short peptides (up to three residues), monopeptide dimers, small molecules, and snapshots from reaction pathways at the ωB97M-D4/ma-def2-TZVPP level of theory^95,96,100,106–110^ in TIP4P-FB water.^111^ The oligopeptides and miniproteins in the BMS25 data set were used as external test set (see below). The resulting AMP-BMS model was then used to perform ML/MM MD simulations of multiple systems of increasing complexity.

### Calibration of the Electrostatic Embedding

In QM/MM calculations, the compatibility between the classical force-field parameters and the QM Hamiltonian remains a long-standing and well-recognized challenge,^115–121^ which also translates to ML/MM setups.^83^ This issue arises from the restricted functional form of classical force fields and their parametrization done independently from the QM methods they are combined with. Nonbonded force-field terms are typically optimized to reproduce thermodynamic properties of liquids, such as the density and enthalpy of vaporization rather than energies, geometries, and other microscopic properties that match QM calculations.^122,123^ As a result, force-field parameters are effective parameters that may compensate for neglected interaction terms in the restricted functional form. For instance, TIP3P charges are overpolarized to compensate for the neglect of explicit polarization modeling.^124^ Such overpolarized MM partial charges lead in turn either to an overpolarization of the electron density of the QM region, which introduces an unphysical “spill-out” effect, i.e., the erroneous localization of QM electron density onto MM atoms due to the lack of Pauli repulsion, or to an overestimation of the electrostatic-interaction energy between QM and MM regions.^27,125–127^

For the AMP-BMS/MM approach, we investigated two strategies to address potential overpolarization and the resulting imbalance between ML/ML and ML/MM interactions. The first strategy, hereafter referred to as *polarization scaling*, damps multipoles induced in the ML zone by the MM zone. This scaling was applied *ad hoc* after the AMP model was trained through the parameter λ_pol_ ∈ [0, 1] in eq 18. In the limiting case λ_pol_ = 0, mechanical embedding is recovered. The second strategy, termed *charge scaling*, follows established practice in QM/MM simulations.^128,129^ In charge scaling, MM charges are scaled by λ_ch_ ∈ [0, 1] in eq 18 and eq 20, damping the magnitude of both the induced and the permanent multipoles. Both schemes apply only to interactions between ML and MM particles at inference and involve a single parameter (either λ_pol_ or λ_ch_) independent of distance, charge magnitude, or polarizability.

The parameters λ_pol_ and λ_ch_ were calibrated using hydration free energies (HFE) of 25 organic molecules selected for their chemical similarity to amino acid side chains. HFEs quantify the thermodynamic cost of transferring a solute from the gas phase into aqueous solution and have found widespread use in the validation and parametrization of classical force fields.^83^ Details of the absolute HFE simulation protocol and molecule selection pipeline are provided in Section S1 in the Supporting Information.

Results for both damping schemes are shown in Figure 2. For charge scaling, optimal performance was found for λ_ch_ = 0.9, yielding a mean absolute error (MAE) of 3.0 ± 0.7 kJ mol^–1^ with respect to experimental values. For polarization scaling, the optimum was identified at λ_pol_ = 0.35, with an MAE of 2.6 ± 0.5 kJ·mol^–1^. Notably, when using their respective optimal coefficients, both scaling schemes outperform the HFE values computed with the classical force field GAFF^112^ reported in the FreeSolv database^113^ (MAE of 5.3 kJ·mol^–1^). These findings were further corroborated by the radial distribution function (RDF) between the oxygen atom of an ML water molecule and the oxygen atoms of the surrounding MM water molecules (right panel of Figure 2), symmetry-adapted perturbation theory (SAPT) interaction energies of dimers, and NOE-derived inter-residue distances for the tryptophan cage protein (details of the simulation protocol and results see Sections S2–S4 in the Supporting Information).

**Figure 2:**
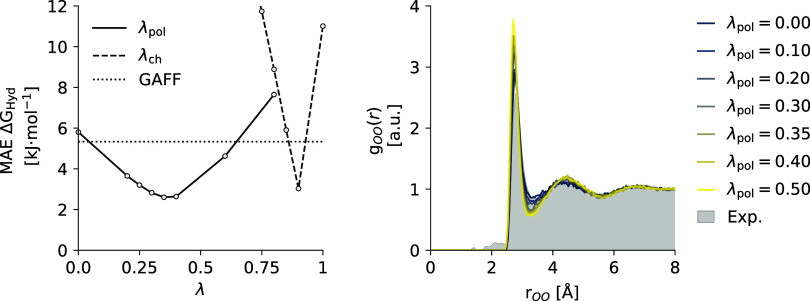
(Left): Mean absolute error (MAE) of computed hydration free energies of 25 small molecules with respect to experiment for different damping coefficients using polarization scaling (λ_pol_, solid) and charge scaling (λ_ch_, dashed). The dotted horizontal line indicates the MAE reported for GAFF^112^ for the same subset of molecules^113^ (see Supporting Information). (Right): Radial distribution function (RDF) between the oxygen atom of the central (ML) water molecule and the surrounding MM water molecules for different polarization scaling coefficients. Experimental values (gray shading) were taken from Sorenson et al.^114^

Results obtained for either scaling scheme were overall very similar and therefore only the results obtained by the polarization scaling with λ_pol_ = 0.35 are reported in the main text of the manuscript. The corresponding results for the charge scaling model are provided in Sections S1–S4 in the Supporting Information. For future applications of the AMP-BMS model, we recommend adopting the polarization scaling scheme, as it allows for easier system-specific calibration when necessary. In summary, these findings demonstrate that solute–solvent ML/MM interactions were significantly overestimated in the absence of damping, highlighting the need for calibration of ML/MM interactions in multiscale schemes. We recommend using the HFEs of small molecules as a calibration target for ML/MM interaction energies. This approach is advantageous because it does not require the extensive sampling needed to assess protein structural stability and can be performed in parallel across many molecules. Additionally, several databases provide experimental reference values, covering diverse functional groups and solvents.^113,130^

### Validation on Benchmarking Data Sets

The accurate representation of the potential-energy surface at a high level of theory is a necessary condition for the development of predictive NNPs. We validated AMP-BMS first on our own external test set of oligopeptides and miniproteins in water in the BMS25 data set^93^ to establish its ability to accurately reproduce energies and gradients of its reference method (ωB97M-D4/​ma-def2-TZVPP^95,96,100,106–110^ ) and second on several literature benchmarks *in vacuo* to assess its accuracy compared to QM calculations at the semiempirical, DFT, and CCSD­(T) levels of theory.

#### Benchmarks in the Condensed Phase

Training data sets of NNPs rarely feature extended chemical structures due to the unfavorable scaling of the reference QM calculations with increasing system size. NNPs must thus generalize from smaller systems presented during training to larger systems during inference. To investigate such an ability to generalize, the AMP-BMS model trained on short peptides, small molecules, and reaction pathways was evaluated on the oligopeptides and miniprotein subsets of the BMS25 data set.^93^ These subsets were designed to uncover potential size or charge biases present in NNPs and include random sequences of oligopeptides with different sizes and charge (4–12 residues, −2 to +2 charge) and miniproteins (166–487 atoms, −2 to +8 charge). The molecules in these subsets are significantly larger than the largest molecule in the training set (84 atoms).

Results for the oligopeptides are shown in Figure 3. Mean absolute errors (MAEs) for the gradients in the ML zone were constant between different molecule sizes but increased slightly with the total charge of the peptide. While median deviations of the gradient components are in the low single digit kJ·mol^–1^ Å^–1^ range, a few large outliers were observed. We hypothesize that these outliers may stem from small deviations of hard degrees of freedom. The MAE of the relative potential energy per atom decreases slightly with increasing system size, indicating that errors grow slower with increasing system size than the total energy, which we deem a desirable feature. The same analysis is reported for the miniprotein subset in Table 1. The results are comparable to those of the oligopeptides and again no clear correlation between system size and error magnitude was observed. In summary, no notable increase of error could be detected during the application of AMP-BMS/MM to increasingly large systems. This observation held for both charge and system size. Based on these results, we conclude that AMP-BMS can be expected to reliably predict gradients and energies at the target level of theory also for systems larger than those presented during training.

**Figure 3:**
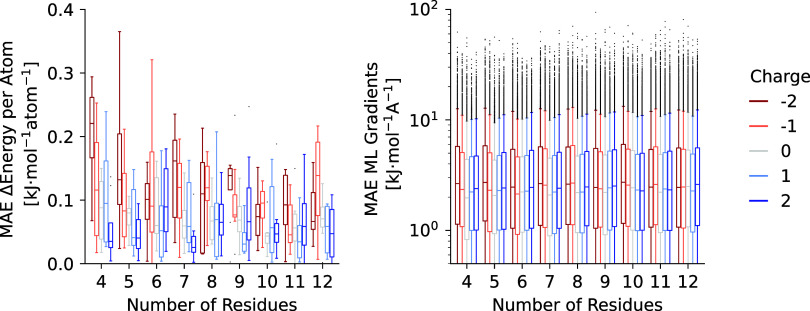
Mean absolute error (MAE) between AMP-BMS/MM and QM/MM for the relative electronic energy per atom between an optimized and off-equilibrium conformations (left) and gradients within the ML zone (right) for the oligopeptides subset in the BMS25 data set^93^ as a function of the number of residues. Boxes cover the range from the first to the third quartile. The center of the box indicates the median. Whiskers show the farthest point lying within 1.5× interquartile range and fliers indicate data points outside of that range. The color encodes the total charge of the oligopeptides. For each combination of charge and number of residues, eight peptides with unique sequences are included.

**Table 1:** Performance of AMP-BMS/MM for the Miniproteins in the BMS25 Dataset:^93^ PDB Identifier for Each Miniprotein, Number of Atoms in the ML Zone, Total Charge, Mean Absolute Error (MAE) with Respect to the Reference DFT Method (*ω*B97M-D4/ma-def2-TZVPP^95,96,100,106–110^ ) for the Total Potential Energy Per Atom (MAE ΔEnergy), the Gradients within the ML Zone (MAE ML Gradients), and the Gradients within the MM Zone (MAE MM Gradients)

System	# Atoms ML Zone	Total Charge	MAE ΔEnergy	MAE ML Gradients	MAE MM Gradients
(PDB ID)			[kJ·mol^–1^atom^–1^]	[kJ·mol^–1^ Å^–1^]	[kJ·mol^–1^ Å^–1^]
2RVD	166	–2	0.3	4.0	0.2
2JOF	284	0	0.1	4.2	0.3
1L2Y	304	1	0.1	4.5	0.3
8TFV	351	6	0.1	4.1	0.3
1M4F	361	4	0.2	4.6	0.4
1CQ0	413	2	0.1	4.6	0.4
1LFC	451	8	0.2	4.7	0.3
1KGM	486	1	0.2	5.0	0.4
1T5Q	487	–2	0.1	3.9	0.4

To assess the quality of the electrostatic embedding in the AMPv3-EE-BMS25 model, we benchmarked the induced dipole moments and the embedding energy decomposition against DFT reference calculations (see Sections S12 and S13 in the Supporting Information for computational details, methodology, and results). The model reproduces the direction and magnitude of induced dipoles with reasonable accuracy for short-range interactions (median angular deviation 14°), while long-range performance is as expected weaker due to the smaller induced moments involved. A systematic underestimation of induced dipole magnitude (MAE = 0.035 eÅ) is observed, consistent with the component-resolved embedding energy analysis, which reveals that the accurate total embedding energy at λ_pol_ = 1.0 arises from near-perfect cancellation between an overestimated electrostatic-interaction term *E*_12_ (mean signed error (MSE) = +24.6 kJ mol^–1^) and an underestimated polarization cost *E*_3_ (MSE = −24.0 kJ mol^–1^). Reducing λ_pol_ to 0.35 suppresses this cancellation and yields a model that effectively approximates the total embedding energy by *E*_12_ alone, with a systematic underestimation relative to the DFT reference. The fact that this systematic underestimation leads to improved agreement with experimental observables constitutes direct evidence that the DFT embedding energy itself is too high due to the overly large fixed charges of the nonpolarizable MM water model. Refining the description of polarization further will be part of future model developments. Note, however, that a model with a perfectly accurate polarization description will not necessarily reproduce experimental data quantitatively, as systematic errors of the underlying DFT method with respect to experiment will require empirical corrections to fully account for.

#### Benchmarks *In Vacuo*

The AMP-BMS model was trained on condensed-phase data (i.e., electrostatic-embedding QM/MM). To assess its performance in the absence of a solvent, the model was evaluated on different gas-phase benchmarks found in the literature. We hypothesized that the modular nature of the AMP architecture, which only considers an external field in the final step of message passing, makes generalization to vacuum environment straightforward. A good performance in the gas phase is, for example, central for the calculation of hydration free energy as used in the previous section.

First, we considered all amino acids and their protonated variants included in the BMS25 data set and benchmarked AMP-BMS against the reference DFT method (ωB97M-D4/ma-def2-TZVPP^95,96,100,106–110^ ). To obtain gas-phase amino acid conformers for the present benchmark, we sampled 50 frames from the condensed-phase MD trajectories of the amino acids in water used in the original BMS25 study, which had served as the basis for generating the water–amino acid dimers employed to assess electron-density leakage. All water molecules were removed from these configurations prior to evaluation. Encouragingly, AMP-BMS achieved consistently low errors for the amino acids, despite not being trained on gas-phase data. The MAE was computed per amino acid and then averaged across all amino acids. The average MAE of the relative conformer energy is 1.93 kJ·mol^–1^, while the average MAE of the individual force components is 2.07 kJ·mol^–1^ Å^–1^ (see Table S5.1 in the Supporting Information).

Next, we benchmarked AMP-BMS on literature gas-phase data sets assessing relative conformer energies and compared the performance with three additional methods: (i) BMS25 DFT method (ωB97M-D4/ma-def2-TZVPP^95,96,100,106–110^ ), (ii) MACE-OMol-0,^42,45,131^ which is a NNP trained on the extensive OMol25 data set,^132^ and (iii) the semiempirical method GFN2-xTB^133^ as baseline. The results are summarized in Table 2.

**Table 2:** Mean Absolute Error (MAE) in Relative Conformer Energies in the Gas Phase with Respect to Coupled Cluster Reference for Benchmark Datasets Using Four Methods: (i) AMP-BMS, (ii) DFT with *ω*B97M-D4/ma-def2-TZVPP^95,96,100,106–110^ (Short as *ω*B97M-D4), (iii) Semiempirical GFN2-xTB,^133^ and (iv) NNP MACE-OMol-0 (Trained on OMol25,^132^ *ω*B97M-V/def2-TZVPD Level of Theory)^a^

Data set	AMP-BMS	ωB97M-D4	GFN2-xTB	MACE-OMol-0
	MAE [kJ·mol^–1^]	MAE [kJ·mol^–1^]	MAE [kJ·mol^–1^]	MAE [kJ·mol^–1^]
ACONF^134^	1.1	0.6	0.8	0.4
Amino20x4 ^134^	1.6	0.9	4.0	1.0
BUT14DIOL^134^	1.5	0.1	5.2	0.3
MCONF^134^	1.1	0.9	7.2	1.7
PCONF21 ^134^	3.5	1.6	7.4	2.3
SCONF^134^	3.7	0.5	6.9	0.9
UPU23 ^134^	5.2	–^d^	12.2	2.2
GLUCOSE^142^	7.2	3.5	26.8	2.5
MALTOSE^142^	7.5	1.8	13.1	1.9
37CONF8 ^135^	4.2	–^e^	18.7^b^	1.4
WIGGLE150 ^146^	6.8	5.1	61.1^c^	3.7

a All values were computed by the authors unless stated otherwise.

b Data are taken from ref 135.

c Data is taken from ref 146.

d Data is not available in the literature sources, SCF convergence issues prevented computations at the target level of theory.

e Data is not available in the literature sources, and its generation was not considered in the present work.

AMP delivers consistently high accuracy results across the GMTKN55 ^134^ subsets (reference method: coupled cluster-based methods with complete basis set extrapolation techniques, for details see ref 134) and the 37CONF8 ^135^ data set (reference method: DLPNO-CCSD­(T)/cc-pVTZ^136–141^ ). For the GMTKN55 subsets, the MAE values are below the chemical accuracy threshold (4.184 kJ·mol^–1^), with the sole exception of UPU23, where the error slightly exceeds this value. The MAE for 37CONF8 is just at the threshold. For the data sets of glucose and α-maltose conformers^142^ (reference method: DLPNO-CCSD­(T)/CBS­(3,4)^136–139,143–145^ with def2-TZVPP and def2-QZVPP basis sets) and the Wiggle150 ^146^ data set of 150 highly strained conformations of adenosine, benzylpenicillin, and efavirenz (reference method: DLPNO-CCSD­(T)/CBS), AMP-BMS yields higher MAE values around 7 kJ·mol^–1^. For glucose and Wiggle150, also the BMS25 DFT reference method shows higher deviation from the coupled cluster reference, which explains the majority of the increased error.

Overall, the performance of AMP-BMS is only slightly worse than MACE-OMol-0 for many benchmark sets, although the latter NNP features 130 times more parameters (52 million trainable parameters in total) and was trained on the 100 million gas-phase calculations in the OMol25 data set. These are encouraging results as they show a straightforward way for improvement of AMP by including gas-phase data in the training set of AMP in the future. For comparison, the errors with the semiempirical method GFN2-xTB^133^ are considerably higher than the NNPs for all benchmark sets except ACONF.

### Evaluation of the Computational Complexity

The computational cost of the AMP-BMS model was evaluated for different biomolecular systems. Seemingly sublinear scaling (*k* = 0.92) was observed for our computational setup when applied to systems involving up to a hundred thousand ML atoms and more than ten million MM atoms on a single NVIDIA H200 GPU with 141 GB VRAM (Figure 4). Large systems used in this benchmark include the delta variant of the SARS-CoV-2 Spike protein (PDB: 7VHH^147^), anthrax toxin receptor (PDB: 3HVD^148^), and the complete satellite tobacco mosaic virus capsid (PDB: 4NIA^149^).

**Figure 4:**
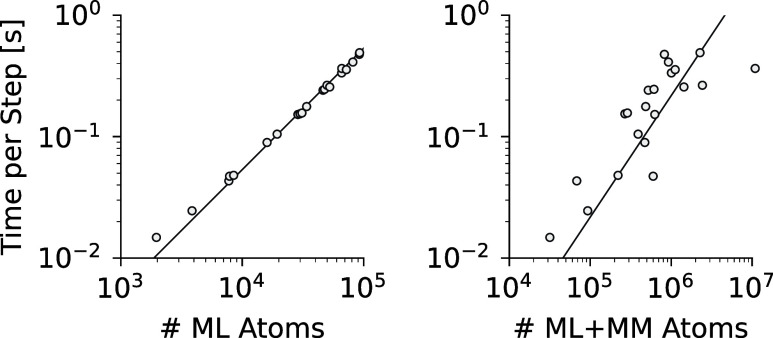
Computational complexity of AMP-BMS/MM on a single NVIDIA H200 GPU with 141 GB VRAM with respect to the number of ML atoms (left) and the total system size including MM atoms (right). Shown is the time per integration step as a function of the number of ML atoms and a linear fit. The number of MM atoms for these systems ranged between 3′000 and 10′000′000 particles.

The favorable scaling suggests that the massive parallelism and specialized hardware components modern accelerators offer is only slowly saturated with increasing system size. On CPU (AMD Ryzen 9 7950X, 196 GB system RAM) linearithmic O(Nlog⁡N) scaling (*k* = 1.15) was observed, which is in agreement with theoretical considerations (see Figure S6.1 in the Supporting Information). Based on the scaling obtained for the GPU (NVIDIA H200 GPU, 141 GB VRAM), a linear fit was applied to assess empirical scaling in practice.[Fn fna] Divided by the number of ML atoms, the simulation speed is 5.1 × 10^–6^ s/ML atom/step and 2.7 × 10^–8^ s/ML atom/step if MM atoms are included. This corresponds to approximately 8.5 ns/day for a system involving 1000 atoms with a 0.5 fs time step. Performing simulations requires approximately 1.4 GB VRAM per 1000 ML atoms (MM atoms have a marginal effect on VRAM). Note that inference speed on more affordable GPU hardware (e.g., RTX 3090) is of course slower than on a H200 GPU. This performance on GPU is comparable with the SO3LR^58^ (3.3 × 10^–6^ s/atom/step) architecture and surpasses other current architectures such as MACE-OFF­(S)^57^ and AIMNet2.^44^ Compared to models like FENNIX,^65^ the cost per step is roughly one magnitude larger if only ML atoms are considered. If both ML and MM atoms are considered, AMP-BMS/MM is approximately one magnitude faster than FENNIX.^59^ Note that in contrast to these NNPs, our setup also includes MM point charges to model the solvent, which add little computational overhead. This means that AMP-BMS/MM uses the majority of computational resources to model the ML zone while maintaining extended solvation shells necessary to avoid cutoff and surface effects.

### Application to Protein Dynamics in Solution

Previous MD simulations of proteins with NNPs have been rather limited in scope and typically feature only simulations over a few nanoseconds in length.^56,58,65^ Similarly to the validation of classical biomolecular force fields, we consider the simulation of proteins in water for extended periods of time an important validation quantity for practical applications of NNPs. For this reason, we performed AMP-BMS/MM MD simulations in triplicates (different random number seed for the initial velocities) of four proteins, which feature a diverse composition of secondary structure motifs:^150^ hen egg-white lysozyme (HEWL, PDB: 1AKI,[Bibr ref151] 1960 ML atoms), major cold shock protein (CspA, PDB: 1MJC,^152^ 1009 ML atoms), protein G (GP, PDB: 1PGB,^153^ 855 ML atoms), and chorismate mutase from *Mycobacterium tuberculosis* (CM, PDB: 2FP2,^154^ 2564 ML atoms). Results for these simulations are shown in Figure 5. For three systems (HEWL, GP, and CM), the backbone RMSD stabilized after approximately 20 ns to a value in the range of 1–1.5 Å, which is similar to RMSD values observed for classical force fields (see Figure S7.1 in the Supporting Information). For CspA, the three replicates show a relatively large variation, which occurs in the residues that form a flexible loop connecting the β-barrel sheets (Figure S15, residues 10–15). The classical force-field simulations show similar behavior (Figure S7.1). Structural features were also validated using the DSSP^155^ analysis (Figure S7.5). For HEWL, we also compared NOE proton–proton distances and ^1^H–^15^N order parameters (*S* ^2^) against experimental data (Section S7.3 in the Supporting Information). AMP-BMS/MM produced results that closely match the experimental measurements, with a total NOE proton–proton distance violation rate of 6% across all replicates and a MAE for *S* ^2^ of 0.045 averaged over all replicates. The results of AMP-BMS/MM using the charge scaling scheme are similar and are provided in Section S7 in the Supporting Information. Taken together, these results confirm that the low errors observed during the validation on QM reference data (see above) translate into stable MD simulations over tens of nanoseconds to reproduce structural features of proteins. In addition, during the publication of this work, we have combined AMP-BMS/MM with REST2-like^156^ enhanced sampling and demonstrated promising results for conformational space exploration, including the folding of peptides and mini-proteins without predefined reaction coordinates.^157^

**Figure 5:**
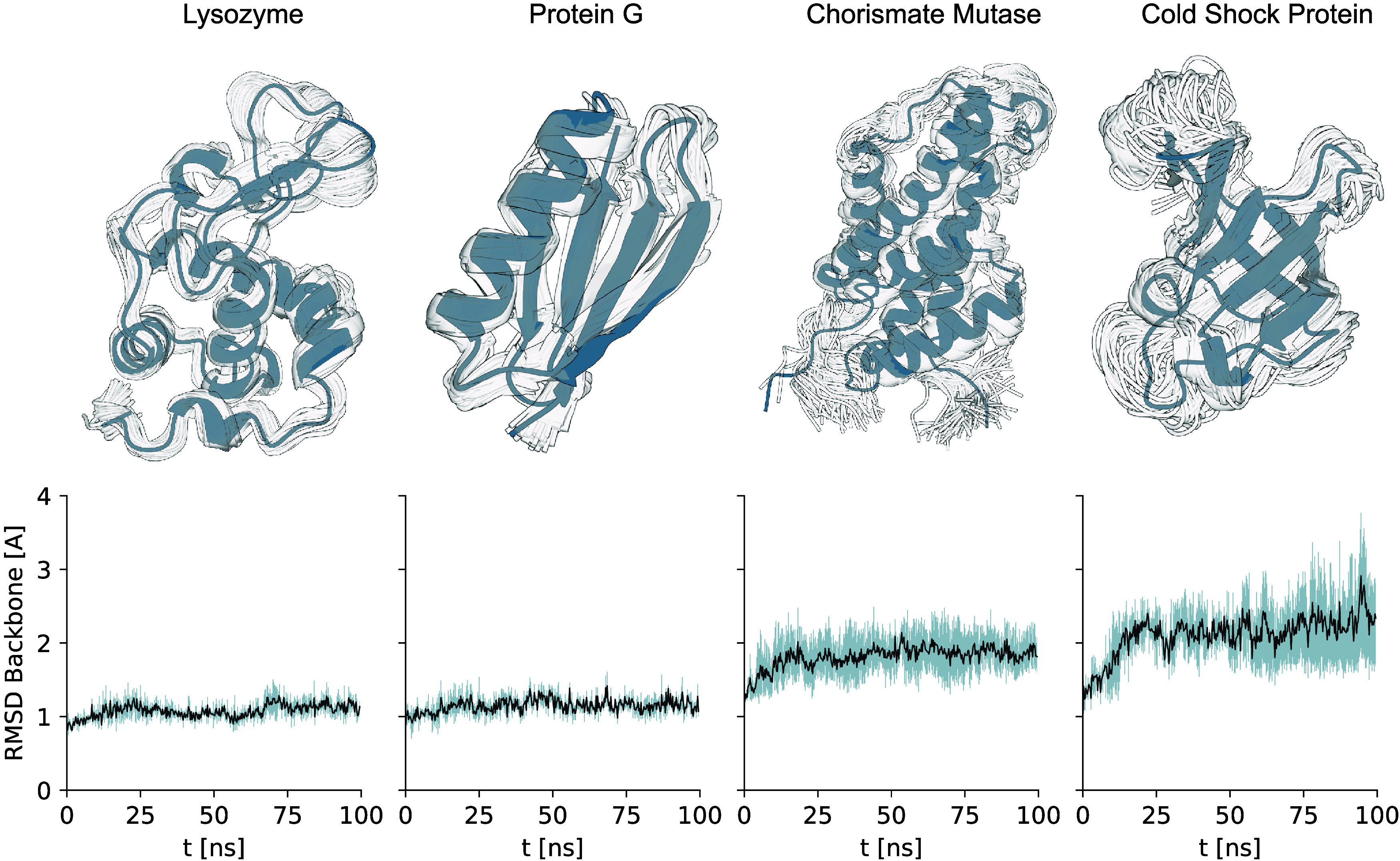
(Top): Snapshots from the trajectory (transparent) overlaid on top of the crystal structure (opaque). (Bottom): Time series of the root-mean-square deviation (RMSD) of the Cα backbone atoms with respect to the crystal structure for four proteins: Hen egg-white lysozyme (1AKI^151^), protein G (1PGB^153^), chorismate mutase (2FP2 ^154^), and major cold-shock protein (1MJC^152^). The average over three replicates is reported by the bold line, while the shaded region indicates the 10–90 percentile range. Results for each replicate are reported in Figure S7.2 in the Supporting Information. The first and last five residues were ignored for the RMSD calculation.

### Application to Melting Curves of Peptides and Miniproteins

At the edge between unfolded and folded structures, protein melting poses a particular challenge to molecular simulation methods. For this phenomenon, small perturbations of the balance between interactions can result in pronounced shifts of the stability of secondary structures, which is not easy to detect when focusing solely on stable protein folds. Previous investigations into protein melting uncovered limitations of classical force fields in reproducing melting curves and showed a tendency to overstabilize certain secondary structure motifs in peptides and proteins.^158–160^ Attempts to reparametrize force fields to improve the description of protein stability and melting resulted in some progress^161,162^ but also highlighted potentially fundamental limitations with classical fixed-charge force fields.

To investigate the ability of AMP-BMS/MM to describe proteins beyond stable folds, a set of four peptides and miniproteins was simulated in triplicates over a temperature range from 278 to 348 K: peptide (AAQAA)_3_, chignolin (PDB: 1UAO^163^), the GB1 hairpin (PDB: 1PGB^153^), and the villin headpiece subdomain (villin, PDB: 1VII^164^). These molecules were previously used to evaluate the force field CHARMM36IDPSFF.^162^ Melting curves and melting temperatures obtained with AMP-BMS/MM are reported in Figure 6 and Table 3, respectively.

**Figure 6:**
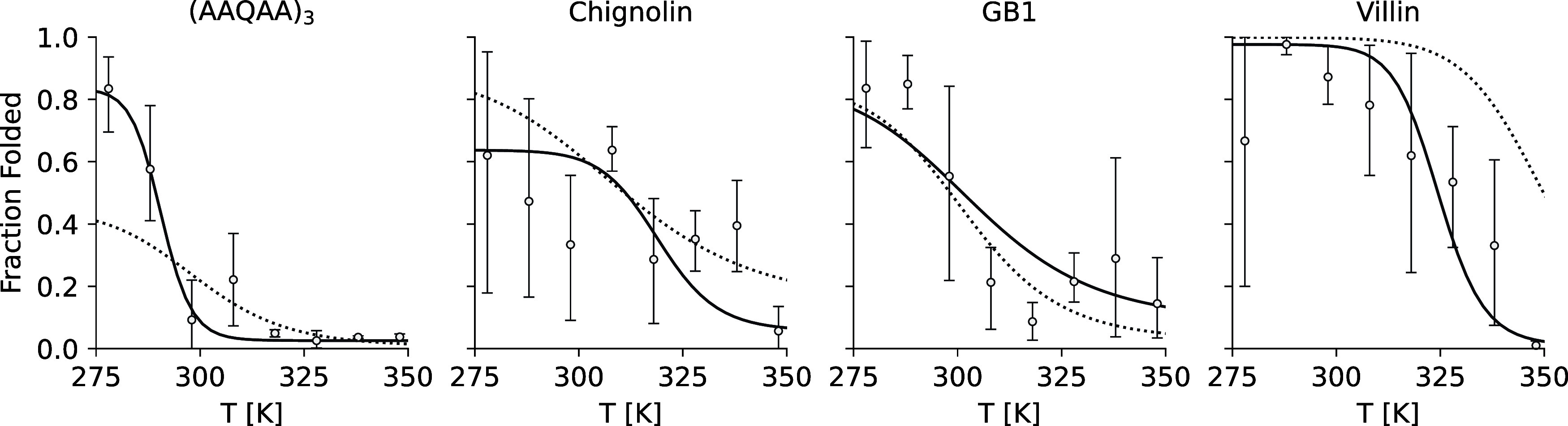
Melting curve for (AAQAA)_3_, chignolin, GB1 hairpin and the villin headpiece subdomain obtained with AMP-BMS/MM (black). Folded states were defined as the α-helix propensity in the case of (AAQAA)_3_ and the fraction of conformations below a RMSD threshold for the miniproteins (see Methods). Dots indicate averages over three replicates of 50 ns length after 50 ns of equilibration, with error bars indicating the 10–90 percentile range. Data were fit to a two-state model (eq 35) indicated by the black line. Melting temperatures obtained with the two-state model are reported in Table 3. For comparison, the experimental melting curve is shown as a dashed line (details provided in Methods). Results for each replicate are reported in Figure S8.4 in the Supporting Information.

**Table 3:** Computed and Experimental Melting Temperature Obtained with AMP-BMS/MM for the Four Systems: (AAQAA)_3_, Chignolin, GB1 Hairpin, and the Villin Headpiece Subdomain^a^

System	*T*_*m*_ [K]	*T*_*m*_ [K]
	(AMP-BMS/MM)	(exp.)
(AAQAA)_3_	291	277(4)^165^
Chignolin	319	314(3)^166^
GB1	303	302 ^167^
Villin	325	343 ^164^

a The corresponding melting curves are shown in Figure 6. Melting temperatures were calculated with a two-state model (eq 35). The uncertainties of the experimental measurements (if reported) are given in parentheses.

Due to the limited simulation time per temperature (3 × 100 ns of which the first 50 ns were discarded as equilibration), we started the simulations from the folded structures (unfolding is generally faster to observe than folding). As this simulation time is much shorter than in comparable studies with classical force fields,^161,162^ the results are likely not fully converged. This can be seen in the large variation among the triplicates for some data points. For example, the surprisingly high uncertainty for villin at 278 K was traced back to one replicate unfolding after around 10 ns and only slowly recovering its fold (see Figure S8.4 in the Supporting Information). Despite this limitation, clear trends already emerge: For all systems, good agreement between experimental and computed melting behavior is observed. In contrast, classical force fields struggle to reproduce these melting curves (see Figure 4 in ref 161 for (AAQAA)_3_ and villin with AMBER99SB-disp, Figure 8 in ref 162 for all four systems with CHARMM36IDPSFF, and Figure 7 in ref 169 for chignolin). Performing our own simulations with AMBER ff14SB force field^170^ (200 ns per temperature and replicate) showed similar issues, especially for GB1 and villin (Figure S8.1 in the Supporting Information). The comparatively high α-helix propensity of (AAQAA)_3_ at low temperatures with AMBER ff14SB force field^170^ compared to the results with the other force fields^161,162^ is likely due to the folded starting structure in connection with the comparatively short simulation time. A similar argument is applicable to AMP-BMS/MM (Figure 6). Results for AMBER ff14SB also add evidence that observed qualitative trends hold for this relatively short simulation time (100 ns).

Interestingly, the melting curves are one application where we observe a difference between the polarization and charge scaling schemes of AMP-BMS/MM, with less agreement with experiment using charge scaling (Figure S8.2 in the Supporting Information).

Overall, these results highlight one of the most notable strengths of NNPs trained on QM data: Without any specific training on proteins and ensemble properties, AMP-BMS/MM reproduces both the dynamics of folded proteins (see above) and protein melting characteristics, which suggests that the approach provides a physically meaningful representation of these systems and an accurate balance between solute–solute and solute–solvent interactions in the ML/MM scheme. In our opinion, melting curves help to establish a connection between a property on which the model was trained (atomic interactions) and an emerging observable (structure) and are therefore valuable validation targets for NNPs.

### Application to Enzymatic Reactions

The study of chemical reactions is an important and attractive application area of NNPs as they promise the same level of accuracy as the QM reference method but at highly reduced computational cost. We and others have already demonstrated that NNPs can be used to study chemical reactions in solution.^51,171–173^ However, these studies have focused on small system sizes and often require training data specific to the reactions investigated (for examples, see refs 51,174 ). We were interested in investigating whether AMP-BMS/MM can be used directly for the qualitative and quantitative modeling of free-energy profiles of enzymatic reactions. In established QM/MM MD set-ups for enzymes,^1,26,175,176^ the QM zone encompasses only the reactive site and the surrounding protein residues up to a chosen distance cutoff. This partitioning of the system requires the use of linking atoms or other approximations to handle the truncation of chemical bonds at the QM/MM boundary,^177–179^ which introduces additional cutoff artifacts. To reduce the cutoff artifacts, inclusion of thousands of atoms in the QM zone and an electrostatic embedding scheme have been shown to be necessary to obtain converged energies.^180^ Given the architecture and excellent scaling of AMP-BMS/MM, our approach offers an attractive solution to both of these limitations, since all atoms of the enzyme and substrate can be included in the ML zone and only solvent atoms remain in the MM zone, which are included with electrostatic embedding.

We investigated two enzymatically catalyzed reactions with AMP-BMS/MM. The first example is the isomerization of chorismate to prephenate (Figure 7A), which is catalyzed by chorismate mutase and constitutes one of the key steps in the synthesis of aromatic amino acids during the shikimate pathway.^182,183,181^ This reaction is one of the most widely studied enzymatic reactions with QM/MM because the substrate forms no covalent bonds with the enzyme during the reaction, which means that the full enzyme can be assigned to the MM zone and no handling of broken chemical bonds is necessary.^184–189^ Second, we investigated the rate-determining step of the enzymatic degradation of fluoroacetate by fluoroacetate dehalogenase (Figure 7B).^190^ Various steps of this enzymatic reaction have been studied by computational methods in the past.^191,192–195^ Biodegradation of halogenated materials is of high interest due to the long degradation times of fluorinated materials from material science as well as the agrochemical or pharmaceutical industries.^196^ To investigate the catalytic mechanism of this enzyme, it is necessary to include protein residues in the QM zone.

**Figure 7:**
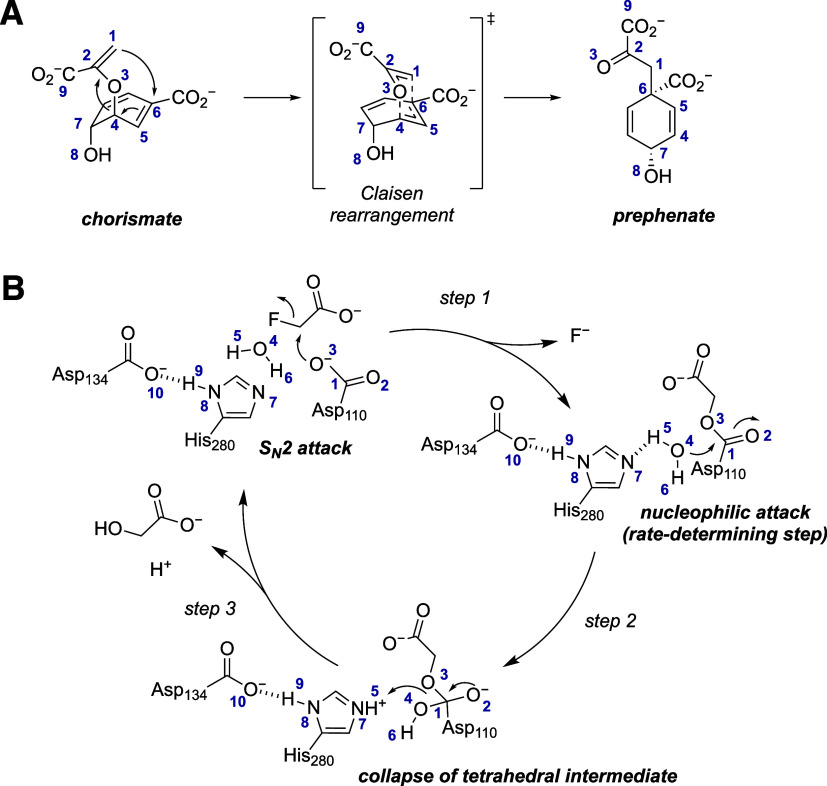
Proposed catalytic mechanism for chorismate mutase^182,183,197^ (**A**) and fluoroacetate dehalogenase^191,196^ (**B**).

#### Chorismate Mutase

The reaction of chorismate to prephenate is a formal Claisen rearrangement (Figure 7A). The reaction has been studied extensively in aqueous solution and enzyme environments using QM/MM set-ups (see e.g., refs 187,189 ) and ML/MM set-ups.^77–79^ However, these previous studies only include the substrate itself in the QM zone or the substrates and protein residues in close proximity to the reactive site to keep the computational effort feasible. Here, we computed the free-energy profile of the transformation of chorismate to prephenate in water (24 ML atoms and 28’000 MM particles) and catalyzed by the enzyme (5687 ML atoms and 85’000 MM particles) with AMP-BMS/MM using umbrella sampling.^198,199^ Consistent with previous studies (see ref 187 and references therein), the reaction coordinate ξ was defined as the difference in length between the breaking bond (O3–C4) and the newly formed bond (C1–C6).

For the reaction in aqueous solution, simulations were performed using three different models: (i) the default AMP-BMS model, (ii) an AMP-BMS model fine-tuned on ωB97M-D4/ma-def2-TZVPP reference data, and (iii) an AMP-BMS model fine-tuned on B3LYP-D4/6-31G­(d) reference data. This setup was designed to demonstrate that both fine-tuning and the choice of reference electronic structure method are critical for obtaining accurate barrier heights in agreement with experiment (see discussion below and the Methods section for fine-tuning details). For the chorismate mutase–catalyzed reaction, three independent replicates were performed using the same default AMP-BMS model to account for statistical variability arising from conformational fluctuations of the enzyme active site and protein backbone. Biased trajectories were reweighted to give the potential of mean force (PMF) (top panels in Figure 8).[Fn fnb] Overall, 323 ns of sampling time per system were achieved.

**Figure 8:**
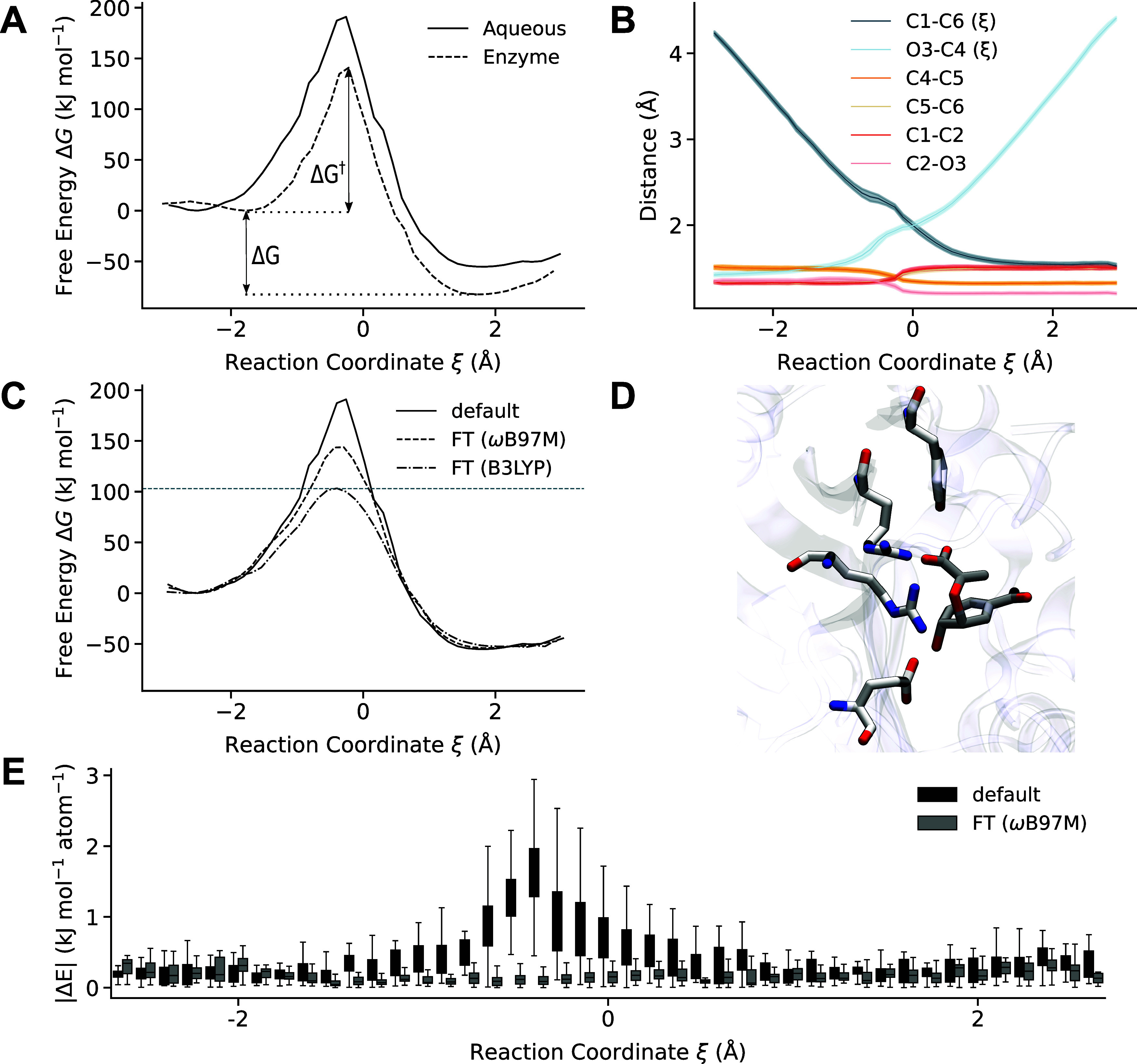
(**A**): Free-energy profiles (potentials of mean force, PMFs) for chorismate isomerization computed with AMP-BMS/MM in water (*aqueous*, solid) and in the active site of chorismate mutase (*enzyme*, dashed). (**B**): Median distances for selected atom pairs as a function of reaction coordinate ξ for the reaction in water computed with the default AMP-BMS model. The variation (interquartiles) across replicates is shown as shading. (**C**): Free-energy profiles for chorismate isomerization in aqueous solution obtained with AMP-BMS/MM without fine-tuning (*default*, solid), and after fine-tuning to ωB97M-D4/ma-def2-TZVPP (FT­(ωB97M), dashed) and B3LYP-D4/6-31G­(d) (FT­(B3LYP), dash-dotted). The experimental barrier height (103 kJ mol^–1^)^200^ is indicated as a blue horizontal line. (**D**): Snapshot of the chorismate molecule in the enzyme pocket from the US window corresponding to the transition state. (**E**): Absolute deviation in per-atom energy between DFT and AMP-BMS/MM as a function of reaction coordinate ξ, evaluated over representative configurations from each umbrella, which were used as the validation set for fine-tuning: default AMP-BMS model (black) and after fine-tuning to ωB97M-D4/ma-def2-TZVPP (gray).

Consistent with experimental data^200,201^ and previous studies,^187,202^ we observed an exergonic reaction (Δ*G* = −55.2 kJ·mol^–1^ in water and −95.7 kJ·mol^–1^ in the enzyme environment, see Table S9.2 in the Supporting Information). The computed free-energy difference in water is thus in agreement with experimental data (Δ*G* = −56.0 kJ·mol^–1^).^201^ We hypothesize that the deviation in Δ*G* observed for the enzyme environment (which should be identical to the aqueous environment) is because the reaction path starts and ends with the protein–substrate rather than the fully separated species. Although AMP-BMS was not trained on this reaction, the geometrical changes in the substrate along the reaction coordinate are as expected (Figure 8B). The distance of the forming C1–C6 bond shows a steep initial decrease, while the bond length of the breaking O3–C4 bond only slowly elongates until the transition state (TS) is reached. Structures of chorismate and prephenate optimized at the ωB97M-D4/ma-def2-TZVPP level of theory with SMD implicit water showed very similar bond lengths (Table S9.4 in the Supporting Information). These findings demonstrate that AMP-BMS/MM simulations result in sampling of physically relevant geometries.

A strong reduction of the reaction barrier height is seen by the enzyme (50.1 kJ·mol^–1^, Figure 8A), which matches experimental data and previous reports^187,189,200,202^ and accounts for the rate acceleration of approximately 10^6^. However, while the relative reduction in Δ*G* ^†^ from water to enzyme matches experiment, the absolute values deviate considerably from the experimentally determined barrier heights of 103 and 64.4 kJ·mol^–1^ in water and enzyme, respectively.^200^ This deviation is likely due to the limited information about TS in the BMS25 data set. To test this hypothesis, we generated a data set of reference QM/MM calculations along the reaction coordinate, which was split into a training (fine-tuning) and validation set. As hypothesized, the default AMP-BMS model exhibits substantially larger errors in the TS region compared to the reactant- and product-like regions for the validation set (Figure 8E), confirming a systematic deficiency (errors for forces, dipoles, and quadrupoles are provided in Figures S9.2 and S9.3 in the Supporting Information). Since AMP-BMS is a foundational model, its accuracy can be systematically improved for a specific target system through fine-tuning.^203–205^ First, we fine-tuned the model to the reference level of theory. As expected, this model exhibits uniformly low errors across the entire reaction coordinate (Figure 8E), confirming that the fine-tuning procedure successfully eliminates the systematic deviation in the TS region. In the resulting PMF of the reaction in water, the barrier height is reduced by 47.2 kJ·mol^–1^ upon fine-tuning (Figure 8C). However, despite this improvement, the barrier remains overestimated relative to experiment, which prompted us to look further. It has been shown that the barrier height of chorismate isomerization spans approximately 65 kJ·mol^–1^ depending on the choice of density functional.^206^ The most important factor governing this variation is the fraction of exact exchange, i.e., increasing the percentage of exact exchange leads to higher predicted barriers. This trend is closely related to the well-known self-interaction error (SIE) inherent to density functional approximations. Functionals with a higher fraction of exact exchange exhibit reduced SIE and consequently yield larger barrier heights.^207^ Conversely, functionals with a lower fraction of exact exchange may benefit from fortuitous error cancellation, leading to improved agreement with experiment despite their underlying approximations. Numerous QM/MM and ML/MM studies have reported barrier heights for chorismate isomerization in water using the B3LYP functional,^79,185,187,202,206^ which were in a very good agreement with experimental values, likely due to such error cancellation effects. Our reference functional, ωB97M-D4, on the other hand, is a range-separated functional and likely exhibits significantly reduced SIE, which in turn diminishes the extent of fortuitous error cancellation. To test this hypothesis, we fine-tuned the AMP-BMS model to the B3LYP-D4/6-31G­(d) level of theory. Indeed, the resulting activation barrier is further reduced and is within 1 kJ·mol^–1^ of the experimental value (Figure 8C). This confirms that some of the deviation originates from the reference functional itself rather than from deficiencies of the ML model. These findings emphasize that a ML model can only be as accurate as the chosen reference method. Numerical values for the activation barriers, reaction thermodynamics, and the positions of the TS along the reaction coordinate for all computed PMFs are summarized in Table S9.2 in the Supporting Information.

#### Fluoroacetate Dehalogenase

The proposed reaction mechanism of enzymatic defluorination involves at least three steps and a catalytic triad formed by Asp110 nucleophile, His280 base, and Asp134 carboxylate (Figure 7B).^191,196^ First, the carboxylate of Asp110 displaces the fluoride in an S_*N*_2 attack to form a covalent ester Asb110 intermediate, and in a second step, the carbonyl group of this intermediate is attacked by a water molecule under concurrent deprotonation. Finally, this tetrahedral intermediate collapses to release, after proton transfer, hydroxy acetate and the enzyme. The water molecule required for saponification is suggested to be deprotonated by His280, which in turn is polarized by Asp134. Structural biology and *ab initio* studies have identified the second step as rate-limiting.^191,196^ Accordingly, we focused on this step to compare computed barrier heights with the experimentally determined rate constants. Although the experimental counterpart is not available, we investigated the Asp134Ala mutation to gauge the ability of the model to predict barrier heights of mutant structures. Our expectation is that this mutation leads to a significant increase in the reaction barrier due to the reduced polarization of the water molecule involved in the second step. We anticipate that this small mutation poses a significant challenge for NNPs, which are often limited to short-range interactions due to their locality *ansatz*, and that AMP-BMS/MM could overcome these limitations due to the explicit incorporation of physics-motivated long-range interactions and modeling of polarization effects.

To obtain reaction profiles, we performed umbrella sampling^198,199^ of fluoroacetate dehalogenase (obtained from PDB: 5K3F, see Methods section) and its Asp134Ala mutant using AMP-BMS/MM (9257 and 9253 atoms in the ML zone, respectively, surrounded by >230’000 MM particles). The reaction coordinate ξ was defined as the antisymmetric combination of bond lengths of newly formed and breaking bonds, i.e., *r*_C_carbonyl_–O_water__ and *r*_O_water_–H_water__. This choice was justified by geometric considerations surrounding the nucleophilic attack of carbonyls^209^ and confirmed by previous QM/MM studies of the enzyme,^191^ which suggest anticorrelation of these two distances over the course of the reaction. For both systems, we performed three replicates with different starting velocities and 47 umbrella windows (2.5 ns per window, 353 ns overall sampling time per system).

The left panel of Figure 9 shows the PMFs of the enzyme-catalyzed reaction. For both enzymes, the reactant and product complexes constitute local minima at ξ = −2.3 Å and 0.3 Å, respectively. In each case, the reactant complex is lower in energy than the product complex (Δ*G* = 58.1 and 82.0 kJ·mol^–1^, respectively), and a single TS as defined by a maximum in free energy was identified. For the wild-type enzyme, the predicted barrier Δ*G* ^†^ was found to be 113 kJ·mol^–1^ at −0.4 Å, which was increased for the Asp134Ala mutant (143 kJ·mol^–1^). The relative increase in barrier height corresponds to a factor of approximately 10^5^ (estimated by the Eyring–Polanyi equation^210–212^ ), which is consistent with the relative decreases in catalytic activity of other enzyme-catalyzed saponification reactions that operate under a similar mechanism.^213–215^ For example, analogous substitutions in trypsin^213^ and subtilisin^214^ resulted in a deceleration of approximately 10^4^–10^5^. As Asp134 is located relatively far from the reacting water and carbonyl group (∼8 Å), the results suggest that AMP-BMS/MM is capable of describing such rather long-range effects. Similarly to our findings on chorismate isomerization, the absolute barrier heights are too high compared to the experimentally determined value of 78.4 kJ·mol^–1^ ^196^ and we anticipate that this can also be corrected by extending the BMS25 data set and/or fine-tuning the AMP-BMS model.

**Figure 9:**
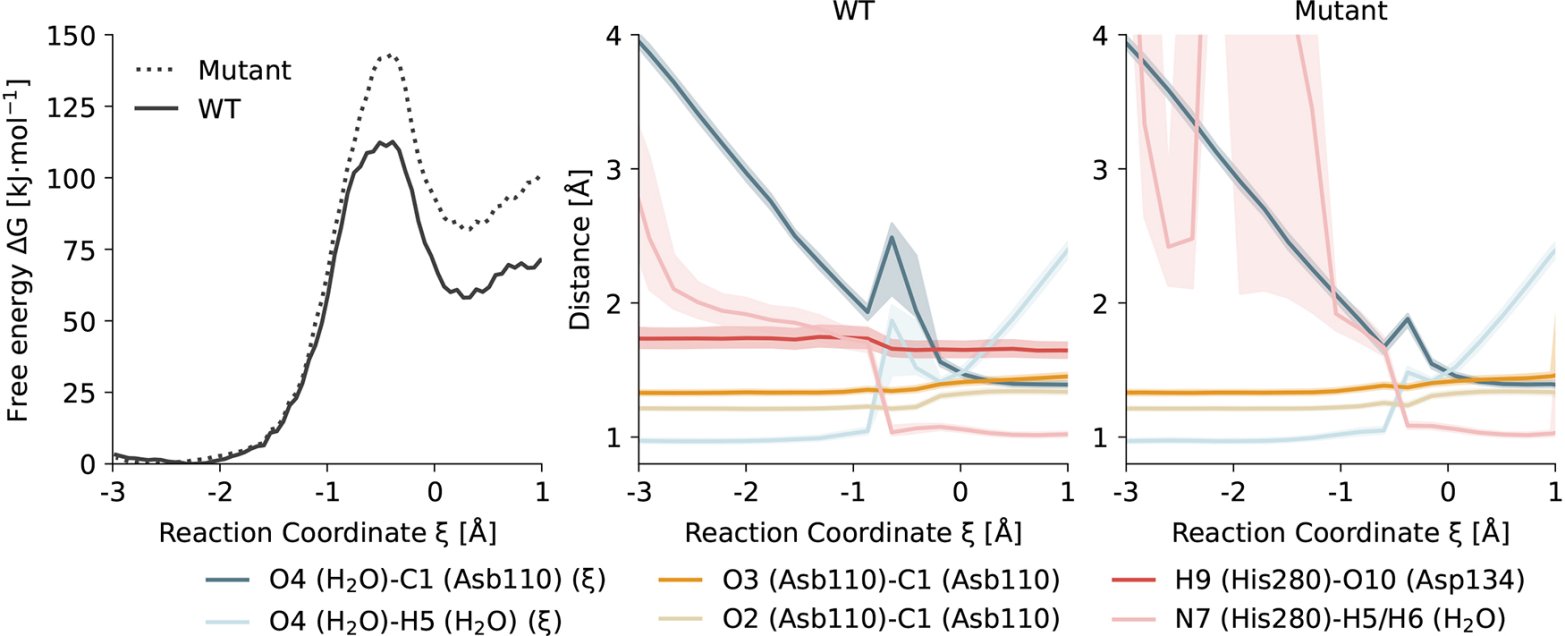
(Left): Free-energy profiles (potentials of mean force, PMFs) of the rate-determining step of fluoroacetate degradation calculated with AMP-BMS/MM via umbrella sampling catalyzed by the wild-type enzyme (solid, dark gray, 9257 ML atoms) or the Asp134Ala mutant (dotted, light gray, 9253 ML atoms) surrounded by 230’000 MM particles. (Central, right): Median distances of selected atom pairs as a function of reaction coordinate ξ for the wild-type enzyme (central panel) and the Asp134Ala mutant (right panel). The variation (interquartiles) across replicates is shown as shading.

Based on the simulations, we can compare the geometric characteristics of the reaction mechanism (Figure 7B) between the wild-type and mutant enzymes (right panels of Figure 9). For the wild-type enzyme, the initial part of the nucleophilic attack involves a steep reduction of the distance between the water oxygen atom O4 and carbonyl C1 (Asp110), while the distance between O4 and the proton H5 (His280) remains almost the same. The approach of water to the carbonyl is correlated with a reduction of the distance between the nitrogen atom N7 (His280) and either proton of the water molecule (H5/H6).[Fn fnc] Formation of a new carbon–oxygen bond is then preceded by deprotonation of water by His280. During this phase of the reaction, both the C1_Asp110_-O4_H_2_O_ and O4_H_2_O_-H5_His280_ distances increase to approximately 2.1 and 2.6 Å, respectively. After deprotonation, a new bond is formed between O4 and C1. These changes in atom distances and bond lengths are consistent with the expected changes in hybridization states. Overall, similar geometric changes are observed along the reaction coordinate for the Asp134Ala mutant. However, we note several important differences. First, the distance between the water proton (H5/H6) and N7 (His280) is longer during the initial phase of the reaction as the water is much more mobile. This may be a direct result of the mutation, which results in a lower degree of polarization of His280. While the maximum in free energy Δ*G* ^†^ occurs at the same value of the reaction coordinate (ξ = −0.4 Å) for the wild-type and mutant enzymes, deprotonation of water by His280 is delayed in the Asp134Ala mutant, which suggests that this step is a higher-energy process for the mutant that lacks the distant polarization of His280 by Asp134.

In some trajectories of both enzymes, we observed spontaneous collapse of the tetrahedral intermediate under formation of hydroxy acetate and the regenerated enzyme at reaction coordinates ξ greater than 1.0 Å (step 3 in Figure 7B). While the sampling of this step was insufficient to calculate meaningful PMFs, its spontaneous occurrence suggests that the barrier associated with this process is relatively low, which is in agreement with previous reports.^191,196^

Based on these findings, we conclude that the default AMP-BMS model can already be used in ML/MM simulations without fine-tuning to investigate relative trends in enzymatically catalyzed reactions, which is an important milestone in the development and application of NNPs. Future extensions of the BMS25 data set will improve the estimation of absolute barrier heights. In the meantime, fine-tuning (to the appropriate functional) can already improve agreement with experiment.

## Conclusion

The next generation of the AMP architecture was trained on our recently published BMS25 data set to perform electrostatic-embedding ML/MM simulations of biomolecules in solution. We demonstrated that the model can generalize to extended systems including oligopeptides and miniproteins when trained only on short peptides with three residues or fewer. This approach dramatically reduces the computational cost to generate QM/MM training data. Even though AMP-BMS was trained only on condensed-phase data, the model performs well in established QM benchmarks of relative conformer energies in the gas phase. Crucially, the AMP architecture scales favorably with the number of atoms in the ML zone, which enabled us to include entire proteins in the ML zone surrounded by MM solvent and simulate them with DFT accuracy. Simulations of 100 ns length showed that the proteins remained close to the folded crystal conformation. AMP-BMS/MM simulations of a series of systems showed overall good agreement with experimental melting curves, which suggests that this approach provides a better representation of the underlying physics than classical force fields. Variation across replicates was large though due to relatively short simulations time (100 ns). This can be improved by the application of sampling enhancement techniques.

The favorable scaling of AMP-BMS/MM presents a crucial advantage for the study of enzymatic reactions because the inclusion of the full enzyme in the ML zone removes any artifact due to a too small QM zone and due to cuts of covalent bonds across zones. We applied our approach to estimate the free-energy profiles of two enzymatic reactions: the isomerization of chorismate to prephenate and the rate-determining step in the enzymatically catalyzed degradation of fluoroacetate. For both systems, relative trends were in good agreement with experimental data, while absolute barrier heights were overestimated. We traced this discrepancy back to limited coverage of TS structures in the BMS25 data set rather than a limitation in the model architecture as well as the fundamental limitations of the underlying QM reference method. We have demonstrated that the accuracy can be significantly improved in a straightforward manner by fine-tuning to a given system and, if necessary, to the desired functional.

In this study, we demonstrated that AMP-BMS/MM is able to simulate folded proteins in solution over comparatively long time scales and to compute free-energy profiles of enzymatic reactions with the correct relative trends. Both constitute important milestones and steps forward in the development and application of NNPs for (bio)­molecular systems. With the ability to generate increasingly large data sets at a high level of theory, NNPs will further improve in accuracy and thus facilitate the study of biological and chemical systems in the future.

## Materials and Methods

### Model Parameters

Table 4 describes model parameters and the total number of trainable parameters. Since ML/MM does not treat all interactions equally, different cutoffs are used for different interactions. While some cutoffs can be freely chosen and control the trade-off between model accuracy and computational cost (i.e., *r*_SR_, *r*_ML/ML,LR_, and *r*_ML/MM,Pol_), other cutoffs are not part of the training (i.e., *r*_ML/MM,LR_ and *r*_MM/MM_). The latter two cutoffs are only relevant during MD simulations and do not influence the training since the ML/MM LJ interactions are only included during simulations. Both cutoffs are set to *r*_ML/MM,LR_ = *r*_MM/MM_ = 9 Å, as this cutoff value was used in the parametrization of the TIP4P-FB water model.^111^

**Table 4:** Hyperparameters of the AMPv3 Architecture Used in this Work

Message Passing Steps	2
Cutoff Graph/Short Range (*r*_SR_)	4 Å
Cutoff ML/ML Long Range (*r*_ML/ML,LR_)	14 Å
Cutoff ML/MM Polarization (*r*_ML/MM,Pol_)	8 Å
Cutoff ML/MM LJ and Electrostatics (*r*_ML/MM,LR_)	9 Å
Cutoff MM/MM Interaction (*r*_MM/MM_)	9 Å
Node Size	128
Edge Size	32
Bessel Functions ML/ML	8
Bessel Functions ML/MM	2
Multipole Channels	8
Multipole Order	2
Total Trainable Parameters	412’074

### Training and Implementation

The model was implemented in PyTorch/2.2.1.^216^ Model weights were initialized using He initialization.^217^ Neural-network parametrized functions ϕ were implemented using fully connected neural networks and the Swish nonlinearity.^218^ Model parameters were optimized using Adam^219^ with default parameters (β_1_ = 0.9, β_2_ = 0.999, ϵ = 10^–7^). All models were trained for 24 epochs using an exponentially decaying learning rate. Initial and final learning rates are reported in Table 5. Gradients were clipped using a max global norm of 1.^220^

**Table 5:** Hyperparameters used during training of AMP-BMS

α	0.99
β	100
γ	100
ζ	1
Initial Learning Rate	5·10^–4^
Final Learning Rate	10^–5^
Epochs	24

The model was trained by optimizing the following objective:

24 $$L=\left(1-\alpha \right)\cdot L_{\mathrm{Energy}}+\alpha \cdot L_{\mathrm{Gradients},\mathrm{ML}}+\beta \cdot L_{\mathrm{Gradients},\mathrm{MM}}+\gamma \cdot L_{\mathrm{Dipoles}}+\gamma \cdot L_{\mathrm{Quadrupoles}}+\zeta \cdot L_{C6}$$

composed of loss terms for the potential energy,

25 $$L_{\mathrm{Energy}}={\left(\Delta \mathrm{V̂}-\Delta V\right)}^{2}$$

the gradients within the ML zone,

26 $$L_{\mathrm{Gradients},\mathrm{ML}}=\frac{1}{3N_{\mathrm{ML}}}\sum_{i}^{N_{\mathrm{ML}}}\sum_{\nu =1}^{3}{\left(-\frac{\partial \mathrm{V̂}}{\partial R_{i,\nu }}-F_{i,\nu }\right)}^{2}$$

gradients within the MM zone,

27 $$L_{\mathrm{Gradients},\mathrm{MM}}=\frac{1}{3N_{\mathrm{MM}}}\sum_{i}^{N_{\mathrm{MM}}}\sum_{\nu =1}^{3}{\left(-\frac{\partial \mathrm{V̂}}{\partial R_{i,\nu }}-F_{i,\nu }\right)}^{2}$$

molecular dipoles,

28 $$L_{\mathrm{Dipoles}}=\frac{1}{3}\sum_{\nu =1}^{3}\left({\left({\mathrm{M̂}}_{\nu }^{1}-M_{\nu }^{1}\right)}^{2}+{\left({\mathrm{M̂}}_{\nu \backslash }^{1}-M_{\nu }^{1}\right)}^{2}\right)$$

molecular quadrupoles,

29 $$L_{\mathrm{Quadrupoles}}=\frac{1}{6}\sum_{\nu =1}^{3}\sum_{\mu =\nu }^{3}\left({\left({\mathrm{M̂}}_{\nu \mu }^{2}-M_{\nu \mu }^{2}\right)}^{2}+{\left({\mathrm{M̂}}_{\nu \mu \backslash }^{2}-M_{\nu \mu }^{2}\right)}^{2}\right)$$

and C6 coefficients,

30 $$L_{C6}=\frac{1}{N_{\mathrm{ML}}}\sum_{i}^{N_{\mathrm{ML}}}{\left({Ĉ}_{i}^{6}-C_{i}^{6}\right)}^{2}$$

with the potential energy *V*, force components *F*_*i*,ν_, atomic positions **R**, molecular multipoles **M** ^**k**^ for Cartesian dimensions ν and μ, and atomic *C*_6_^*i*^ coefficients. Molecular dipoles and quadrupoles were constructed as,

31 $${\mathrm{M̂}}_{\nu }^{1}=\sum_{i}^{N_{\mathrm{ML}}}\left(M_{i}^{0}\cdot R_{i}\right)+M_{i}^{1}$$

and

32 $${\mathrm{M̂}}_{\nu \mu }^{2}=\sum_{i}^{N_{\mathrm{ML}}}\left(M_{i}^{0}\cdot R_{i}^{2}\right)+M_{i}^{2}$$

after removing the center of mass. To shift importance to atomic monopoles, losses for molecular dipole and quadrupoles were computed both with and without atomic multipole terms, i.e.,

33 $${\mathrm{M̂}}_{\nu \backslash }^{1}=\sum_{i}^{N_{\mathrm{ML}}}M_{i}^{0}\cdot R_{i}$$

and

34 $${\mathrm{M̂}}_{\nu \mu \backslash }^{2}=\sum_{i}^{N_{\mathrm{ML}}}M_{i}^{0}\cdot R_{i}^{2}$$

where \ is introduced to indicate that the molecular multipole was computed without contributions of higher order atomic multipoles, i.e., using only atomic monopoles. Coefficients α, β, γ and ζ were introduced to weight the total loss and are given in Table 5. Instead of the total potential energy, the relative energy was used to compute the energy-loss term. The relative energy was computed for each batch as the difference in potential energy between the first and *n*th conformation of the batch. Each batch was constructed from up to 10 conformations of the same molecule.

The model was trained on the monopeptide, dipeptide, tripeptide, reactions, small molecules and dimers subsets of the BMS25 data set.^93^ The validation set was constructed using 1% of these subsets and a random split. The oligopeptide and miniprotein subsets of the BMS25 data set were used as hold-out test set. Only conformations for which the largest absolute component of the gradient within the ML zone was smaller than 2′000 kJ·mol^–1^ Å^–1^ were included. The model currently supports the following chemical elements: H, C, N, O, S, P, F, Cl, Br, and I. The BMS25 data set presently includes 246’464 structures from the Transition1x data set,^221^ which comprises unimolecular chemical reactions involving systems with up to seven heavy atoms and containing the elements H, C, N, and O. At this stage, the BMS25 data set does not include any metal ions, however, support for metal-containing systems is part of future work.

### Calculation of *In Vacuo* Benchmarks

The amino-acid configurations used for the *in vacuo* benchmark described in Section Benchmarks In Vacuo were obtained from stochastic dynamics (SD) trajectories of amino acids in TIP4P-FB water described in ref 93 (where these trajectories were used to sample the water-amino acid dimers for electron-density leakage studies). The solvent was stripped, and the trajectories were subsampled to yield 50 conformers per amino acid. Gas-phase calculations were performed using the same level of theory employed in the BMS25 data set (ωB97M-D4/ma-def2-TZVPP^95,96,100,106–110^ ) with ORCA/5.0.4.^20,222–226^ Electronic energies, forces, molecular dipoles, and molecular quadrupoles were computed. Electronic energies for all structures of data sets ACONF, Amino20x4, BUT14DIOL, MCONF, PCONF21, SCONF, GLUCOSE, MALTOSE, WIGGLE150 were computed at ωB97M-D4/ma-def2-TZVPP^95,96,100,106–110^ level of theory with ORCA/5.0.4 ^20,222–226^ and its default settings. Electronic energies for all structures of data sets ACONF, Amino20x4, BUT14DIOL, MCONF, PCONF21, SCONF, UPU23, GLUCOSE, MALTOSE were also calculated at the GFN2-xTB^133^ level of theory with ORCA/5.0.4 and its default settings. Finally, relative conformer energies for all structures of data sets ACONF, Amino20x4, BUT14DIOL, MCONF, PCONF21, SCONF, UPU23, GLUCOSE, MALTOSE, 37CONF8, WIGGLE150 were computed with MACE-OMol-0.^131^

### Simulation Details

If not noted otherwise, all simulations in this work were performed using SD. SD simulations were carried out with OpenMM/8.1.2 using the OpenMM PyTorch plugin version 1.4.^46,227^ Trajectories were sampled from a NPT ensemble using a Langevin middle integrator with Δ*t* = 0.5 fs and a friction coefficient of 1/ps at 300 K.^228^ A Monte Carlo barostat with a trial frequency of *n* = 100 was applied with a target pressure of 1 bar.^229^

Molecules were solvated in TIP4P-FB water^111^ with OpenMM using a padding distance equal to *r*_MM/MM_. No counterions were added to the simulation box. LJ parameters for particles within the ML zone used to describe the LJ contribution to the ML/MM interaction (see eq 23) were parametrized with AMBER ff14SB force field^170^ for proteins and OpenFF/2.2.0 ^122^ for ligands. The cutoff *r*_MM/MM_ was set to 9 Å. Bonds of the MM water molecules were kept rigid with the SETTLE algorithm.^231^ Long-range electrostatic interactions were treated with the shifted reaction-field algorithm.^94^ Center of mass translation was removed every step. During the enzymatic reaction simulations, coordinates and energies were saved at intervals of 2′000 steps, whereas in the protein dynamics and melting-curve simulations they were saved every 50′000 steps. In the rare cases where simulations collapsed over the course of 100 ns, they were restarted from a previous checkpoint.

### Evaluation of the Computational Complexity

Protein systems were set up as described above and simulated for 2′000 time steps on single CPU core (AMD Ryzen 9 7950X) or GPU (H200). Only the wall-clock time for the last 1′000 time steps was considered for benchmarking to account for warm-up. The scaling coefficient *k* was derived as 1.15 by linear regression of the logarithmic data of the scaling equation *T*(*N*) = *CN* ^*K*^, where *T* is the wall-clock time, *N* the number of ML particles, and *C* is a constant (Figure S6.1 in the Supporting Information). We note that seemingly sublinear scaling on GPU (*k* = 0.92) is most likely an artifact of massive parallelism and hardware accelerators and is not meaningful for determining the theoretical scaling law. Due to the nearly linear relationship of compute time and number of ML particles, we also determined empirical scaling coefficients discussed above (5.1 × 10^–6^ s/ML atom/step and 2.7 × 10^–8^ s/ML atom/step) by linear regression of unprocessed GPU data.

#### Prospective Simulations

Protein simulations were initialized from the experimental crystal structure. For each system, three replicates were simulated for 100 ns each with different random number seeds for the initial velocities. MDTraj (1.10.3)^232^ was used to calculate the atom-positional root-mean-square deviation (RMSD) with respect to the crystal structure and to carry out the DSSP analysis.^155^ The RMSD was calculated over all *C*_α_ backbone atoms excluding the first and the last five residues.

#### Melting Curves

MD simulations of chignolin (PDB: 1UAO^163^), GB1 hairpin (GB1, PDB: 1PGB^153^), and the villin headpiece subdomain (villin, PDB: 1VII^164^) were initialized from the experimentally determined structures. Simulations for (AAQAA)_3_ were initialized from an α-helix conformation generated with PyMol.^233^ Following the procedure in ref 162, folded states for villin, chignolin and GB1 were defined as conformations for which the atom-positional RMSD of the backbone with respect to the experimental structure was smaller than a cutoff (chignolin: 2.0 Å, GB1: 2.5 Å, villin: 3.0 Å). For (AAQAA)_3_, folded states were determined using the fraction of α-helix obtained from a DSSP analysis^155^ as implemented in MDTraj (1.10.3).^232^ For each system and condition, three replicates with different initial velocities were simulated for 100 ns each. For the analysis, the first 50 ns were discarded as equilibration.

Folded fractions were fitted to a two-state model,

35 $$f_{F}\left(T\right)=f_{U}+\frac{f_{F}-f_{U}}{1+\exp\left[-\frac{\Delta H_{m}\left(1-T/T_{M}\right)}{\mathrm{RT}}\right]}$$

to obtain the fraction the melting temperature *T*_m_ and the heat of melting Δ*H*_m_ given the simulation temperature *T. f*_F_ and *f*_U_ were introduced to correct for the fraction of folded states at the highest (*f*_U_) and lowest (*f*_F_) simulated temperature. The data points for the experimental melting curves shown in Figure 6 were extracted from the corresponding figures in the literature using WebPlotDigitizer:^234^ Data for chignolin were taken from ref 166, for the GB1 hairpin from ref 167, and for the villin headpiece subdomain and (AAQAA)_3_ peptides from ref 162.

#### Enzymatic Reactions

##### Set-up and Starting Structures

PDBFixer/1.9 ^46,227^ was used to remove all heterogens and add missing residues, heavy atoms, and protons for pH 7.0 for chorismate mutase (PDB: 2CHT^235^) and fluoroacetate dehalogenase (PDB: 5K3F^236^). For fluoroacetate dehalogenase, the first and last five amino acids were truncated, mutated Asn280 was replaced with natural His280, and one cocrystallized water molecule residing between His280 and Asb110 (Asb: Asp after nucleophilic attack to fluoroacetate) was retained during structure preparation. For the fluoroacetate dehalogenase ligand, the Asp134Ala mutation was performed using PDBFixer. Ligand structures were placed at the positions obtained from the crystal structures. Solvent molecules (TIP4P-FB^111^) were added with PDBFixer and a padding distance of 30 Å. LJ parameters for mutated Asb110 were generated with OpenFF/2.2.0.^122^ The starting structure for chorismate in water was taken from the crystal structure (PDB: 2CHT).

Starting structures for umbrella sampling (US) were generated using steered molecular dynamics (SMD).^237^ In brief, structures were subjected to geometry optimization followed by NPT equilibration for 10 ps, where the reaction coordinate was restrained with a force constant of 100 kJ·mol^–1^ Å^–2^ around a target distance of −2.7 Å (chorismate to prephenate reactions) or −3.0 Å (fluoroacetate dehalogenases). Velocities were generated from a Maxwell–Boltzmann distribution at 298 K. The target distance was then updated for 50 ps with a pulling velocity of 0.2 Å ps^–1^. Structures with values of the reaction coordinate close to US starting structures were retained to obtain 43 structures between −2.6 and 2.6 (chorismate) and 47 structures between −3.0 and 3.0 (dehalogenase). For the chorismate to prephenate reaction in solution, torsions defined by C4, O3, C2, C9 and O8, C7, C4, O3 were transiently restrained at 100 and −154°, respectively, with a force constant of 1.0 kJ·mol^–1^ to avoid elongation of these motifs during SMD. This restraint was removed in the subsequent US simulations.

##### Umbrella Sampling

US simulations of chorismate mutase were performed using 43 windows along the reaction coordinate. Consistent with previous studies (see ref 187 and references therein), the reaction coordinate ξ was defined as the difference between the breaking bond length (O3–C4) and the forming bond length (C1–C6). Minimum distances and force constants used in the US simulations are provided in Tables S9.1 and S9.5 in the Supporting Information. Production runs of 2.5 ns were started directly from the SMD-derived coordinates. Three replicates with different initial velocities were carried out to account for possible conformational fluctuations of the protein backbone and active site.

Each umbrella window for the reaction in water consisted of 0.5 ns of equilibration followed by 2.0 ns of production (single replicate). To prevent dissociation of the transition state (TS) into two fragments, or sampling of unphysical configurations in which the sum of the O3–C4 and C1–C6 bond lengths becomes excessively large, a one-sided harmonic restraint was applied to this distance sum. This restraint was applied only for umbrella windows corresponding to ξ ∈ [−0.7, −0.1], using a force constant of 100 kJ mol^–1^ Å^–2^ and enforcing a maximum allowed total bond length of 5.2 Å. Additionally, during the first 50 ps of equilibration, a one-sided harmonic restraint was applied to the angle defined by vectors along the C2–C9 bond and the C5–H bond (hydrogen covalently bonded to C5 atom). This restraint used a force constant of 0.03 kJ mol^–1^ degree^–2^ and enforced a minimum angle of 140°. It ensured that all structures remained in the desired chairlike conformation and prevented sampling of undesired boat-like TS geometries. This restraint was removed after the initial equilibration period.

##### Potential of Mean Force

For the reaction in water, potentials of mean force (PMFs) were computed using the multistate Bennett acceptance ratio (MBAR^238^) method as implemented in pymbar/4.0.3 program,^238^ with all biasing potentials explicitly included in the analysis. Prior to free-energy reconstruction, each umbrella window was equilibrated and statistically correlated samples were removed using time-series analysis to estimate equilibration time and statistical inefficiency, followed by subsampling to ensure decorrelated configurations.^239^ For the enzyme-catalyzed reaction, biased trajectories were reweighted using the weighted histogram analysis method (WHAM)^240^ as implemented in the WHAM/2.0.11 program.^241^ PMFs to estimate free-energy barriers were computed from the combined statistics of the three replicates. Errors were estimated with Monte Carlo bootstrapping (100 attempts)^242^ as implemented in WHAM.

In case of fluoroacetate dehalogenase, biased trajectories were also reweighted using WHAM.^240^ PMFs to estimate free-energy barriers were computed from the combined statistics of the three replicates. Errors were estimated with Monte Carlo bootstrapping (100 attempts)^242^ as implemented in WHAM.

Reaction barriers Δ*G* ^†^ were then estimated as the maximum free energy between the two local minima during each reaction. This procedure to estimate free-energy barriers has been applied in previous studies, see for example ref 189 and references therein.

#### Fine-tuning the AMP-BMS Model

Coordinates of the substrate in water were sampled from a preliminary US simulation of the chorismate reaction in water performed with the default AMP-BMS model. For umbrella windows close to reactant and product states (ξ_0_ ∈ [−2.6; −0.6] and [0.2; 2.6]), 150 samples were collected for each window. For umbrella windows representing pre-TS and post-TS structures (ξ_0_ ∈ [−0.5; −0.4] and [0.0; 0.1]), 500 samples were collected for each window. For TS-like windows (ξ_0_ ∈ [−0.3; −0.1] and [0.0; 0.1]), 1000 structures were collected for each window. The resulting fine-tuning set comprised 10’400 structures in total. Reference DFT calculations were performed using the same settings as for the BMS25 data set.^93^ The reference data were generated at two levels of theory: ωB97M-D4/ma-def2-TZVPP^95,96,100,106–110^ and B3LYP-D4/6-31G­(d).^95,96,243–248^ The train/validation split was performed randomly within each umbrella window using a 0.9/0.1 ratio. The structures used for fine-tuning at both levels of theory (for both the training and validation splits) were kept identical.

The training settings were kept identical for both fine-tuning campaigns and are the same as described above, including the loss function and its hyperparameters. Both fine-tuning campaigns used an initial learning rate of 2.5 × 10^–4^ and a learning rate decay factor of 5·10^–3^. Fine-tuning to the reference level of theory used 12 epochs, whereas fine-tuning for B3LYP-D4/6-31G­(d) used 14 epochs. The weights of the default AMP-BMS model were used as the starting weights in both fine-tuning campaigns.

## Supplementary Material



## Data Availability

The BMS25 data set used to train AMP-BMS can be found on the ETH Research Collection (10.3929/ethz-c-000788484). An OpenMM + PyTorch implementation of AMP (version 3) is made available on GitHub: https://github.com/rinikerlab/amp_bms.

## Footnotes

a While experiments on CPUs are consistent with theoretical linearithmic O(Nlog⁡N) scaling, we argue that the empirical scaling enabled by accelerators like GPUs is more meaningful in practice.

b We follow the common assumption that the activation free energy is equivalent to the PMF barrier height along a selected reaction coordinate. For a discussion, see e.g., ref 189 and references therein.

c N­(His280) – H (H_2_O) distances were calculated with respect to the proton H5/H6 that is closest to N7 to reflect the fact that water is not tightly bound and we observed rotations resulting in both protons alternating in close proximity to N7.

## Notes

Details on the calibration of the electrostatic embedding formalism, additional data on gas-phase benchmarks and computational complexity, additional figures and tables for protein dynamics, melting curves, and enzymatic reactions (PDF)
