# Supplementary material for: Multiscale Neural Network Potential with Anisotropic Message Passing for the Fast and Accurate Simulation of Protein Dynamics and Enzymatic Reactions
Source: J Am Chem Soc. 2026 Jul 1;148(27):28133–56. doi: 10.1021/jacs.6c00217 (PMC13383635; doi:10.1021/jacs.6c00217)
Supplement: Supplementary file 1 [file ja6c00217_si_001.pdf]

SUPPORTING INFORMATION
Multiscale Neural Network Potential with Anisotropic Message Passing for the Fast and Accurate Simulation of Protein Dynamics and Enzymatic Reactions

Moritz Thürlemann[a,†], Felix Pultar[a,†], Igor Gordiy[a,†], Enrico Ruijsenaars[a], Sereina Riniker[a*]

[a] *Department of Chemistry and Applied Biosciences, ETH Zürich, Vladimir-Prelog-Weg 2, 8093 Zürich, Switzerland. E-mail: sriniker@ethz.ch*

† *These authors contributed equally.*

Contents

S1 Calculation of Absolute Hydration Free Energies **S3**
S1.1 Computational Details S3
S1.2 Results S6

S2 Dimer Interaction Energies **S7**
S2.1 Computational Details S7
S2.2 Results S7

S3 Water Radial Distribution Function (RDF) **S11**
S3.1 Computational Details S11
S3.2 Results S11

S4 Tryptophan-Cage Simulations **S12**
S4.1 Computational Details S12
S4.2 Results S13

S5 Additional Table for Gas-Phase Benchmarks **S18**

S6 Additional Figure for Computational Complexity **S19**

S7 Additional Figures for the Study of Protein Dynamics **S20**
S7.1 Time Series of Cα-RMSD S20
S7.2 Time Series of Secondary Structure Motifs S23
S7.3 NMR Comparison for HEWL S26

S8 Additional Figures for the Study of Miniprotein Melting Curves **S28**
S8.1 Melting Curves S28

S8.2 Times Series of Cα-RMSD S29

S9 Additional Tables and Figures for the Study of Enzymatic Reactions **S32**

S9.1 Chorismate Mutase S32

S9.2 Fluoroacetate Dehalogenase S37

S10 Summary of Differences Between AMPv2 and AMPv3 **S39**

S11 Errors for Molecular Multipoles **S40**

S12 Polarization Quality Benchmark **S40**

S12.1 Computational Details S40

S12.2 Results S41

S13 Electrostatic Embedding Quality Benchmark **S42**

S13.1 Computational Details S42

S13.2 Theory S43

S13.3 Results S44

S1 Calculation of Absolute Hydration Free Energies

S1.1 Computational Details

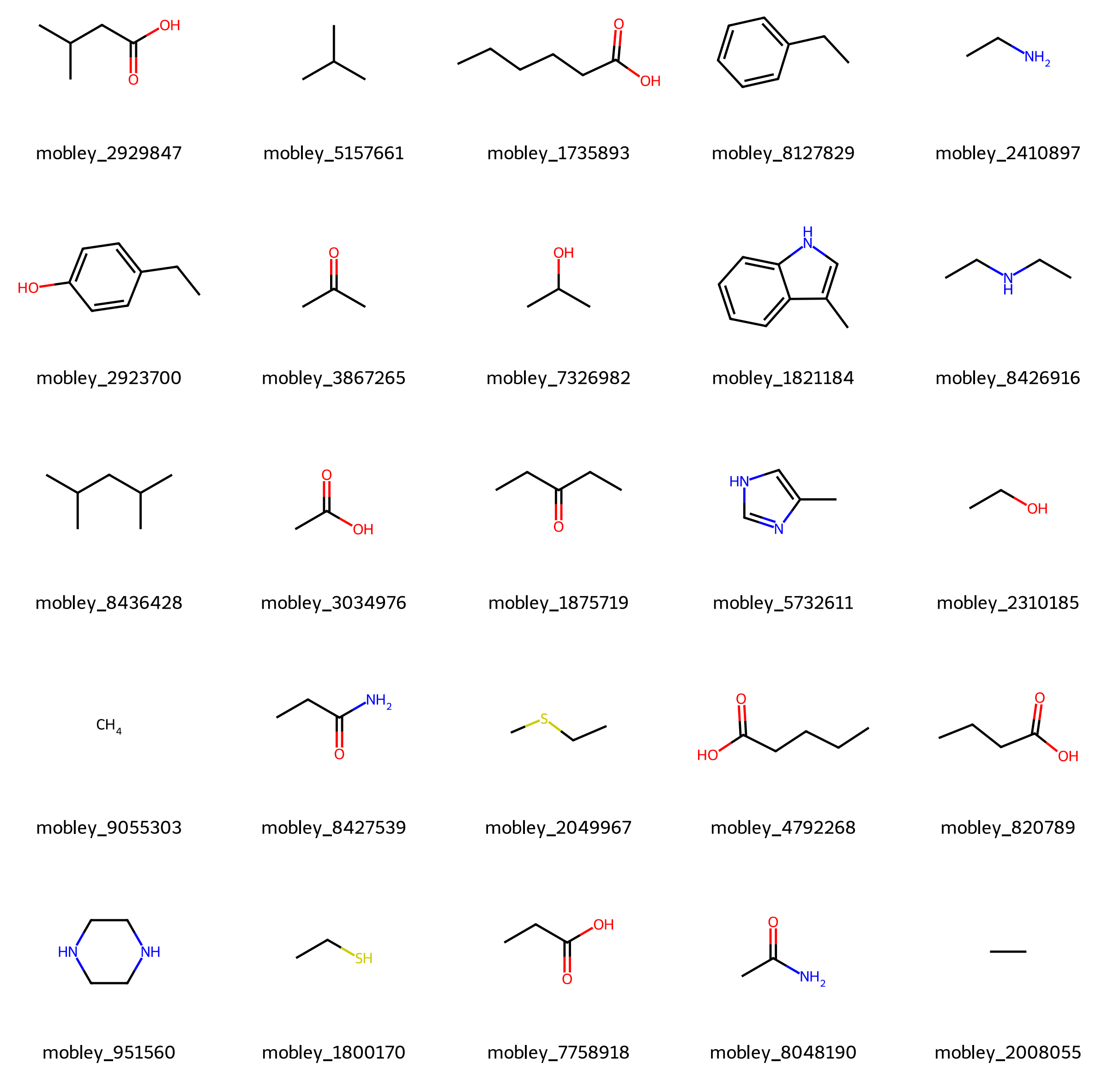

Figure S1.1: Organic molecules selected from the FreeSolv database [1] for the calculation of absolute hydration free energies.

25 neutral molecules (Figure S1.1) were selected from the FreeSolv database/version 0.52 [1] based on their chemical similarity to amino-acid side chains and the glycine molecule. To identify these molecules, the 20 natural amino-acid side chains were capped with a methyl group and converted to the SMILES [2] format. Morgan fingerprints [3] (radius = 3, size = 8192 bits) were computed for each capped residue and

the molecules in the FreeSolv database, and the Tanimoto similarity was calculated between the capped residues and the FreeSolv molecules. For each capped residue, its most similar neighbor in the FreeSolv database was selected together with the 10 most similar molecules to glycine. Duplicate molecules were removed, and those containing vinyl groups were excluded as irrelevant for protein environments, resulting in 25 molecules. All calculations and molecular similarity analyses were carried out using the `RDKit 2024.03.3` package [4].

To calculate the absolute hydration free energies (HFEs) across a range of scaling factors and two scaling schemes, we defined the following thermodynamic states using the three scaling parameters that control the scaling of the electrostatic (λ_{ch}), Lennard-Jones (LJ) (λ_{LJ}, and polarization interactions (λ_{pol}:

- **State 1**: $\lambda_{\text{ch}} = 1$, $\lambda_{\text{LJ}} = 1$, $\lambda_{\text{pol}} = 1$
  Represents the default scheme with no scaling of the ML/MM electrostatic interactions and the LJ interactions fully present.
- **State 2**: $\lambda_{\text{ch}} = 0$, $\lambda_{\text{LJ}} = 1$, $\lambda_{\text{pol}} = 0$
  Represents a system that is fully electrostatically decoupled, while the LJ ML/MM interactions are fully present.
- **State 3**: $\lambda_{\text{ch}} = 0$, $\lambda_{\text{LJ}} = 0$, $\lambda_{\text{pol}} = 0$
  Represents a fully decoupled system between the ML and MM zones, with both electrostatic and LJ ML/MM interactions scaled to 0.0.
- **State 4**: $\lambda_{\text{ch}} = 1$, $\lambda_{\text{LJ}} = 1$, $\lambda_{\text{pol}} = 0$
  Represents a system in which the polarization scaling factor is 0.0, while the electrostatics and LJ ML/MM interactions are fully present.

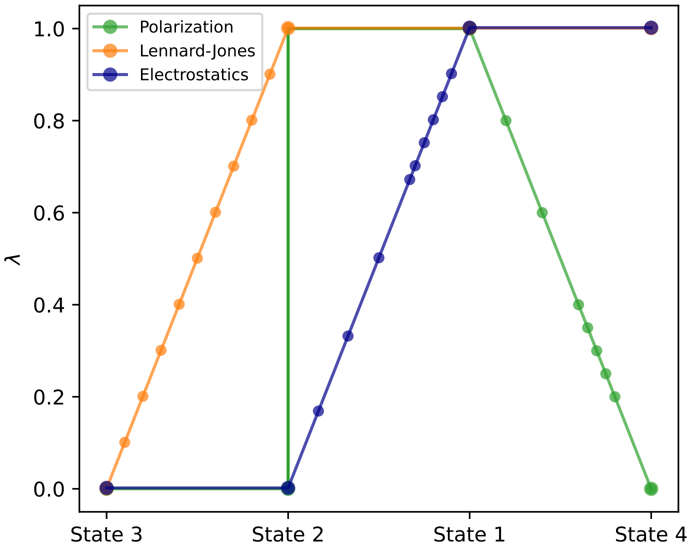

Figure S1.2: λ-scaling of the different contributions across the three alchemical transformations, with the simulated λ-windows shown as points. Starting from the fully decoupled state, the LJ interactions are first turned on, followed by the electrostatics to reach the physical end state 1. The polarization is fully switched on as soon as electrostatic interactions start contributing. To compute the HFEs at different polarization levels, the fully depolarized state is generated by scaling down the polarization interactions while keeping electrostatic interactions intact.

The HFEs for different λ_{ch} values of the charge scaling scheme were calculated using the thermodynamic path $State\ 3 \rightarrow State\ 2 \rightarrow State\ 1$, which corresponds to transferring the ML molecule from the gas phase to MM solution. The procedure is summarized as follows:

- **Lennard-Jones ML/MM coupling** ($State\ 3 \rightarrow State\ 2$): 11 λ-windows with λ_{LJ} = [1.0, 0.9, 0.8, 0.7, 0.6, 0.5, 0.4, 0.3, 0.2, 0.1, 0.0]
- **Electrostatic ML/MM coupling** ($State\ 2 \rightarrow State\ 1$): 11 λ-windows with λ_{ch} = [1.0, 0.9, 0.85, 0.8, 0.75, 0.7, 0.67, 0.5, 0.33, 0.167, 0.0]
- **Polarization ML/MM coupling** ($State\ 1 \rightarrow State\ 4$): To compute the HFEs for different λ_{pol} values of the polarization scaling scheme, an additional path calculation is required: 9 λ-windows with λ_{pol} = [1.0, 0.8, 0.6, 0.4, 0.35, 0.3, 0.25, 0.2, 0.0]

SD simulations for all λ-windows were performed with OpenMM/8.1.2 using the OpenMM PyTorch plugin version 1.4 [5, 6]. Trajectories were sampled from a NPT ensemble using a Langevin middle integrator with $\Delta t = 0.5$ fs and a friction coefficient of $1/\mathrm{ps}$ at 298.15 K [7]. A Monte Carlo barostat with a trial frequency of $n = 2000$ was applied with a target pressure of 1 bar [8]. The TIP4P-FB water model [9] was used with a cutoff of 0.9 nm, and a long-range correction for the LJ potential [10] was applied. Molecules were solvated using the Modeller tool in OpenMM 8.1.2. [6] with 2.0 nm padding. Bonds of the MM water molecules were kept rigid with the SETTLE algorithm [11]. Long-range electrostatic interactions were treated with the shifted reaction field algorithm [12]. Center of mass translation was removed every step. Coordinates and energies were written out every 200 steps. Crashed simulations were restarted from a previous checkpoint. Each system was equilibrated for 100 ps under NPT

conditions, followed by 2.1 ns of production MD per molecule and λ-window (except 4-ethylphenol, which required 4.1 ns per λ-window for convergence). ML zone molecules were parametrized using the OpenFF-2.2.0 force field [13]. Free-energy differences between states were computed using the multistate Bennett acceptance ratio (MBAR) estimator as implemented in `pymbar/3.1.1` [14–16]. The standard error of the mean absolute error (MAE) estimate was computed by bootstrapping. The results of the calculations are provided in Table S1.1.

S1.2 Results

Mobley ID	Polarization scaling ΔG [kJ·mol^{-1}]	Charge scaling ΔG [kJ·mol^{-1}]	Exp. ΔG [kJ·mol^{-1}]
mobley_2929847	-28.3	-25.8	-25.5
mobley_5157661	10.2	9.2	9.6
mobley_1735893	-25.8	-23.4	-26.0
mobley_8127829	-5.0	-8.1	-3.3
mobley_2410897	-19.8	-19.7	-18.8
mobley_2923700	-23.4	-25.8	-25.7
mobley_3867265	-14.8	-13.0	-15.9
mobley_7326982	-22.3	-22.3	-19.8
mobley_1821184	-23.3	-28.6	-24.6
mobley_8426916	-12.4	-17.1	-17.0
mobley_8436428	7.2	5.7	11.8
mobley_3034976	-22.9	-18.6	-28.0
mobley_1875719	-13.3	-13.0	-14.3
mobley_5732611	-38.7	-39.8	-43.0
mobley_2310185	-21.5	-20.5	-20.9
mobley_9055303	9.8	9.5	8.4
mobley_8427539	-38.6	-35.1	-39.3
mobley_2049967	1.6	-4.0	-6.3
mobley_4792268	-28.8	-26.2	-25.8
mobley_820789	-27.2	-24.3	-26.6
mobley_951560	-40.3	-47.4	-31.0
mobley_1800170	0.1	-3.2	-4.8
mobley_7758918	-27.8	-24.6	-27.0
mobley_8048190	-40.4	-36.6	-40.6
mobley_2008055	10.4	9.7	7.7

Table S1.1: Summary of absolute hydration free energies computed for 25 molecules taken from the FreeSolv database [1]. For each molecule, we report its Mobley ID, the experimental reference value, and the results obtained using both the polarization scaling ($\lambda_{\mathrm{pol}} = 0.35$) and charge scaling ($\lambda_{\mathrm{ch}} = 0.9$) approaches. All energies are expressed in kJ·mol^{-1}.

S2 Dimer Interaction Energies

S2.1 Computational Details

Configurations of water–amino acid dimers for the dimer interaction energy calculations were sampled from short stochastic dynamics trajectories of amino acids in TIP4P-FB water, as generated in the BMS25 dataset [17] (these trajectories were used to sample the dimer configurations to study electron-density spill-out effects). Details of the simulation protocol are provided in the Supporting Information of the BMS25 dataset publication. For the dimer interaction energy calculations, each dimer consisted of the solute monopeptide (ML level) and one of its three nearest water molecules (MM level). One dimer interaction energy calculation was performed per water molecule, resulting in 300 monopeptide–water dimer interaction energy calculations per monopeptide. The full QM dimer interaction energies were computed at SAPT2+/aug-cc-pVDZ [18–22] and ωB97M-V/def2-QZVPPD [23–25] levels of theory using Psi4/1.9.1 [26, 27] and ORCA/6.0.1 [28–33] software packages accordingly.

S2.2 Results

First, the quality of the ML/MM interactions was assessed by comparing dimer interaction energies computed with AMP-BMS/MM and symmetry-adapted perturbation theory (SAPT) [34–36]. SAPT is widely used to assess dimer interaction energies without basis set superposition error (BSSE). [37] There are various levels of SAPT that differ in their treatment of electron correlation, which is reflected in the cost-accuracy ratio of the method. Identified as silver standard by Parker *et al.* [38], SAPT2+/aug-cc-pVDZ was selected for this study for its advantageous balance between cost and accuracy as the reference method.

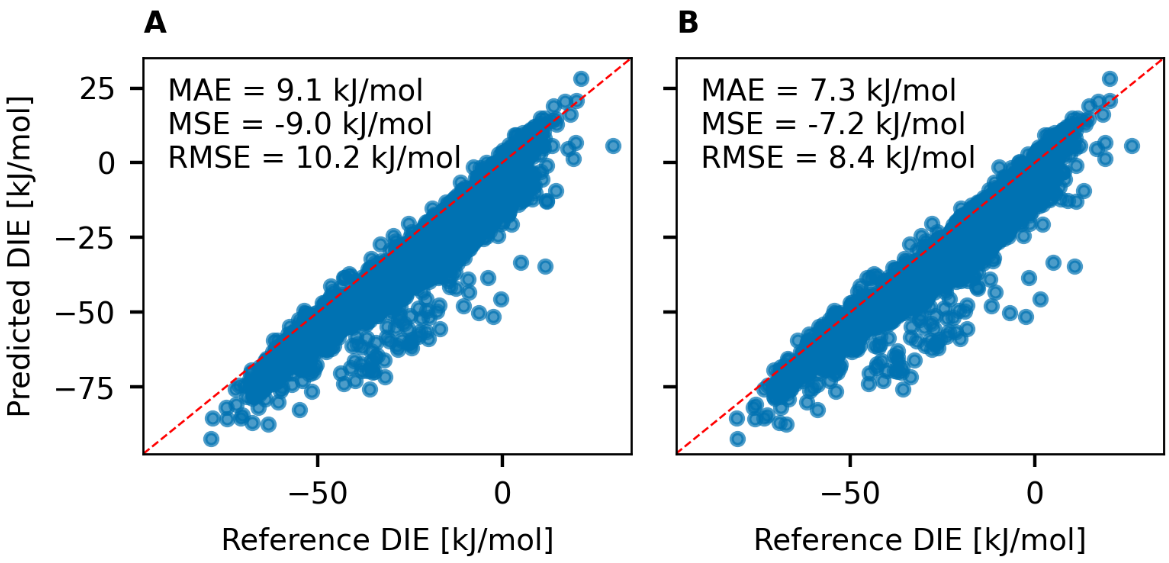

Figure S2.1: Correlation of dimer interaction energy (DIE) between a monopeptide and a TIP4P-FB water molecule for AMP-BMS/MM versus the reference calculation: (A) reference SAPT2+/aug-cc-pVDZ and (B) reference ωB97M-V/def2-QZVPPD. The red dashed line indicates $y = x$. The mean absolute error (MAE), mean signed error (MSE), and root mean squared error (RMSE) are reported in the subplots.

The correlation between dimer interaction energies (DIEs) computed with SAPT2+/aug-cc-pVDZ and AMP-BMS/MM is shown in Figure S2.1A. The ML/MM calculations systematically overestimate the interaction energies relative to the full QM treatment. Such overestimation can promote protein unfolding, as solute–solvent interactions are favored over solvent–solvent interactions. Figure S2.1B shows the same analysis for reference interaction energies computed with supermolecular approach using ωB97M-V/def2-QZVPPD), confirming that the AMP-BMS model overestimates the ML/MM interaction energies.

To assess the influence of λ_{pol} and λ_{ch} on the ML/MM DIEs, we performed parameter scans in 11 equidistant steps over the interval $[0, 1]$ for both scaling schemes. For polarization scaling, the MAE decreases monotonically with decreasing λ_{pol}, with the smallest value at $\lambda_{\text{pol}} = 0$ (Figure S2.3). The other metrics show similar trends. While the results clearly indicate that $\lambda_{\text{pol}} < 1.0$ should be used, setting $\lambda_{\text{pol}} = 0.0$ would not make sense because this eliminates all polarization effects from the MM zone, effectively reducing the model to mechanical embedding. Therefore, additional experiments were necessary to identify an optimal λ_{pol} value for polarization scaling. Charge scaling has a much more pronounced effect on the DIE (Figure S2.3). The best error metrics are obtained at $\lambda_{\text{ch}} \in [0.7 - 0.8]$, yielding an MAE of 3.7 kJ mol^{-1} and RMSE $\in [4.8; 5.8]$ kJ mol^{-1}. The correlation metric plateaus for $\lambda_{\text{ch}} > 0.6$. These results suggest that the optimal λ_{ch} value lies within the interval $[0.6, 1.0)$.

Figures S2.4 and S2.5 summarize the same analysis for the reference method ωB97M-V/def2-QZVPPD. The previously described trends for both scaling schemes are conserved.

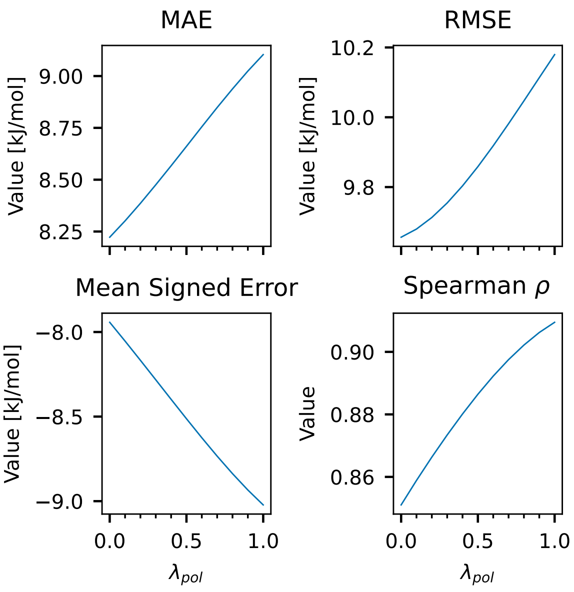

Figure S2.2: Error statistics (MAE, RMSE, MSE, and Spearman correlation coefficient) of the ML/MM dimer interaction energy (DIEs) between a monopeptide and a TIP4P-FB water molecule as a function of λ_{pol} using SAPT2+/aug-cc-pVDZ as reference.

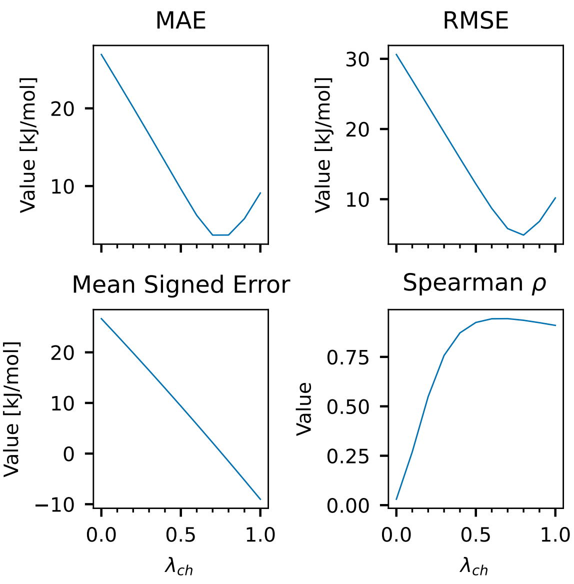

Figure S2.3: Error statistics (MAE, RMSE, MSE, and Spearman correlation coefficient) of the ML/MM dimer interaction energy (DIEs) between a monopeptide and a TIP4P-FB water molecule as a function of λ_{ch} using SAPT2+/aug-cc-pVDZ as reference.

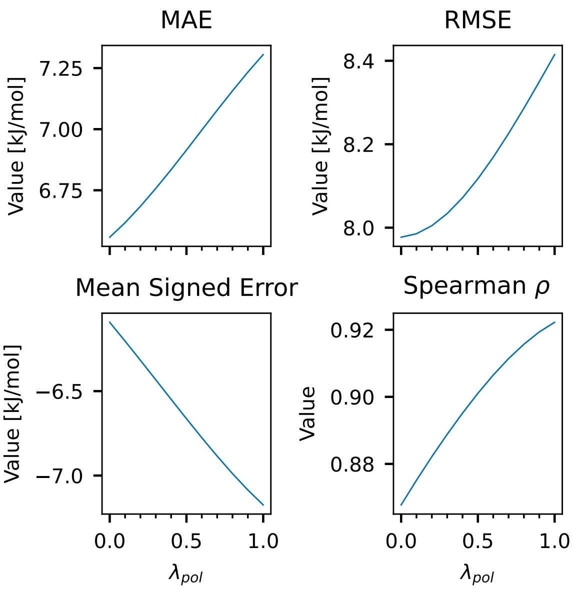

Figure S2.4: Error statistics (MAE, RMSE, MSE, and Spearman correlation coefficient) of the ML/MM dimer interaction energy (DIEs) between a monopeptide and a TIP4P-FB water molecule as a function of λ_{pol} using ωB97M-V/def2-QZVPPD as reference.

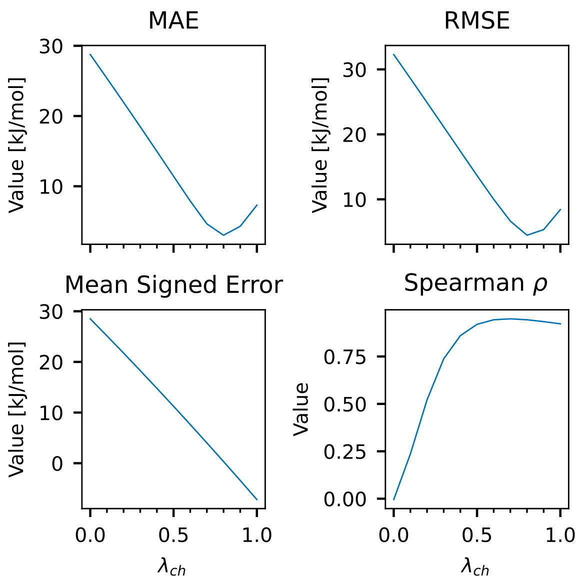

Figure S2.5: Error statistics (MAE, RMSE, MSE, and Spearman correlation coefficient) of the ML/MM dimer interaction energy (DIEs) between a monopeptide and a TIP4P-FB water molecule as a function of λ_{ch} using ωB97M-V/def2-QZVPPD as reference.

S3 Water Radial Distribution Function (RDF)

S3.1 Computational Details

SD simulations for ML water molecule in MM solution were performed with OpenMM/8.1.2 using the OpenMM PyTorch plugin version 1.4 [5, 6]. Trajectories were sampled from a NPT ensemble using a Langevin middle integrator with $\Delta t = 0.5$ fs and a friction coefficient of $1/\text{ps}$ at 298.15 K [7]. A Monte Carlo barostat with a trial frequency of $n = 2000$ was applied with a target pressure of 1 bar [8]. The TIP4P-FB water model [9] was used with a cutoff of 0.9 nm, and a long-range correction for the LJ potential [10] was applied. Water molecule in ML zone was solvated using the Modeller tool in OpenMM 8.1.2. [6] with 2.0 nm padding. Bonds of the MM water molecules were kept rigid with the SETTLE algorithm [11]. Long-range electrostatic interactions in MM zone were treated with the shifted reaction field algorithm [12]. Center of mass translation was removed every step. Coordinates and energies were written out every 200 steps. Crashed simulations were restarted from a previous checkpoint. The system was equilibrated for 100 ps under NPT conditions, followed by 900 ns of production SD. No counterions were added. ML zone water molecule was parametrized using the OpenFF-2.2.0 force field[13]. The reference experimental data were taken from the work of Sorenson *et al.* [39]

S3.2 Results

RDFs, $g(r)$, provide detailed insight into the local solvation structure and hydrogen-bonding patterns. The first peak in the oxygen-oxygen RDF of water, $g_{\text{OO}}(r)$, reflects the preferred hydrogen-bonding distance between water molecules, while subsequent oscillations indicate the extent of structural ordering in the solvent. By simulating one ML water molecule in MM water, we can compute the oxygen-oxygen RDF and deviations from experimental RDF will indicate deficiencies in the ML/MM interactions.

The results for the polarization scaling are shown in Figure 2 in the main text. Figure S3.1 presents the same analysis for the charge scaling scheme. In this case, $\lambda_{\text{ch}} = 0.9$ reproduces the experimental RDF best, while lower λ_{ch} values (< 0.9) resulting in diminished local solvation structure. This suggests that excessive scaling leads to underestimation of ML/MM interaction energies.

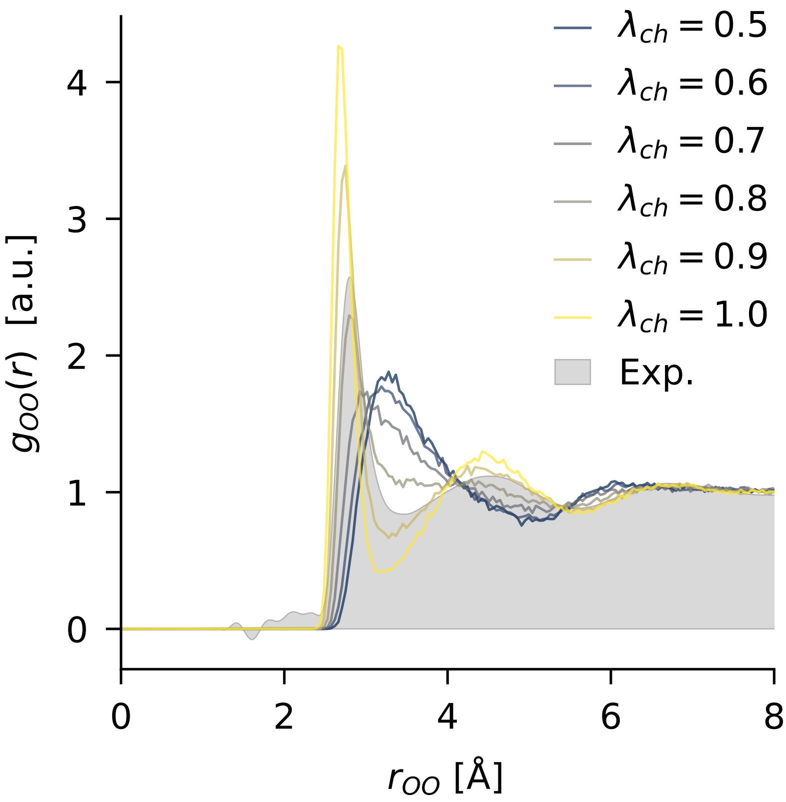

Figure S3.1: Radial distribution function (RDF) of between the oxygen atom of the central (ML) water molecule and the surrounding MM water molecules for different charge scaling coefficients $\lambda_{\text{ch}} \in [0.5, 1.0]$. Experimental values (gray shading) were taken from Sorenson *et al.* [39]

S4 Tryptophan-Cage Simulations

S4.1 Computational Details

Starting coordinates were taken from the Protein Data Bank (PDB) [40] (PDB identifier: 1L2Y [41]). Water molecules removal from crystal structure and protonation were performed using AmberTools22 [42]. Terminal caps were subsequently added using PyMOL/3.0.4 [43]. The structure was solvated using the Modeller tool in OpenMM 8.1.2. [6] with 2.0 nm padding. SD simulations were performed with OpenMM/8.1.2 using the OpenMM PyTorch plugin version 1.4 [5, 6]. Trajectories were sampled from a NPT ensemble using a Langevin middle integrator with $\Delta t = 0.5$ fs and a friction coefficient of $1/\text{ps}$ at 293.15 K [7]. A Monte Carlo barostat with a trial frequency of $n = 2000$ was applied with a target pressure of 1 bar [8]. The TIP4P-FB water model [9] was used with a cutoff of 0.9 nm, and a long-range correction for the LJ potential [10] was applied. Bonds of the MM water molecules were kept rigid with the SETTLE algorithm [11]. Long-range electrostatic interactions in MM zone were treated with the shifted reaction field algorithm [12]. Center of mass translation was removed every step. Coordinates and energies were written out every 100 steps. Crashed simulations were restarted from a previous checkpoint. The system was equilibrated for 2 ns under NPT conditions, followed by 8 ns of production SD.

No counterions were added. The experimental NOE distance bounds were taken from the PDB entry (1L2Y [41]), and the pairs of protons analyzed were adopted from Zhou *et al.* [44].

S4.2 Results

The Trp-cage miniprotein has been studied extensively both *in silico* [44–47] and experimentally [41], making it an excellent calibration system for AMP-BMS/MM. Overestimation of protein-water interactions (which corresponds here to ML/MM interactions) is expected to result in increased solvent-accessible surface area and eventual unfolding of the protein. Importantly, the size of Trp-cage allows for reference DFT calculations.

Figure S4.1 shows the time series of the C_α-RMSD with respect to the energy-minimized Trp-cage structure with different charge scaling coefficients. Trp-cage unfolds (RMSD $>$ 2 Å) within the first 2 ns when no scaling factor is applied ($\lambda_{\text{ch}} = 1.0$). In contrast, the RMSD remains below the unfolding threshold when $\lambda_{\text{ch}} < 1.0$. The same analysis is shown in Figure S4.2 for the polarization scaling scheme. Similar trends are observed. In this case, the protein remains folded when $\lambda_{\text{pol}} \leq 0.5$.

Next, we compared the inter-residue proton-proton distances with experimental NOE data taken from Ref. [41]. Figures S4.3 and S4.4 show the average distances for 28 pairs of protons, each group averaged over chemically equivalent protons. For the charge scaling scheme (Figure S4.3), $\lambda_{\text{ch}} \in [0.8, 0.9]$ yields the lowest percentage of NOE distance violations, ranging from 7.1% to 10.7%. Lower λ_{ch} values result in significantly higher violation rates. For the polarization scaling scheme (Figure S4.4), all $\lambda \in [0.1, 0.5]$ values resulted in low NOE distance violations, ranging from 7% to 13%.

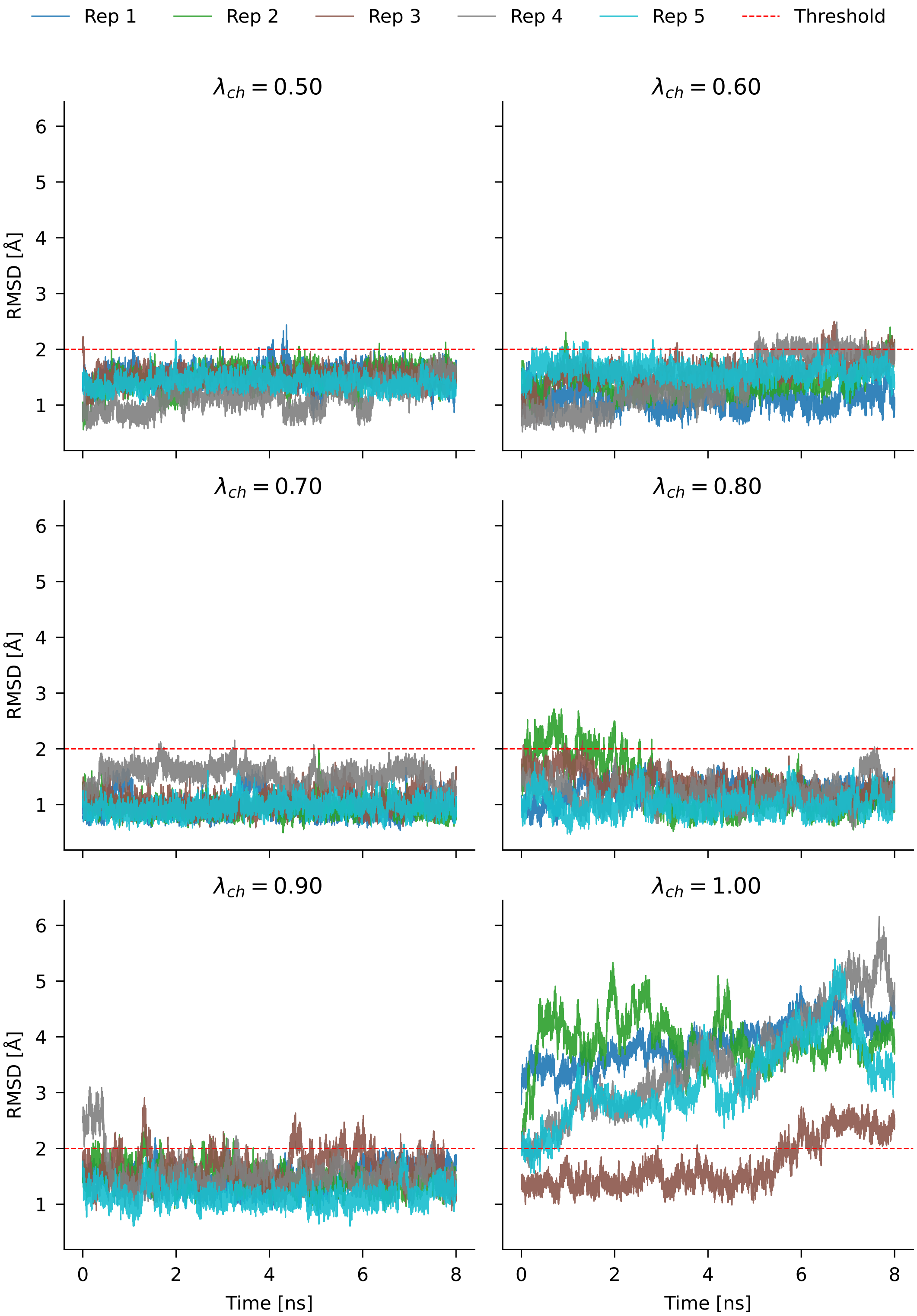

Figure S4.1: Time series of C_α-RMSD (five replicates) of Trp-cage in TIP4P-FB water for the charge scaling scheme, with $\lambda_{\text{ch}} \in [0.5, 1.0]$.

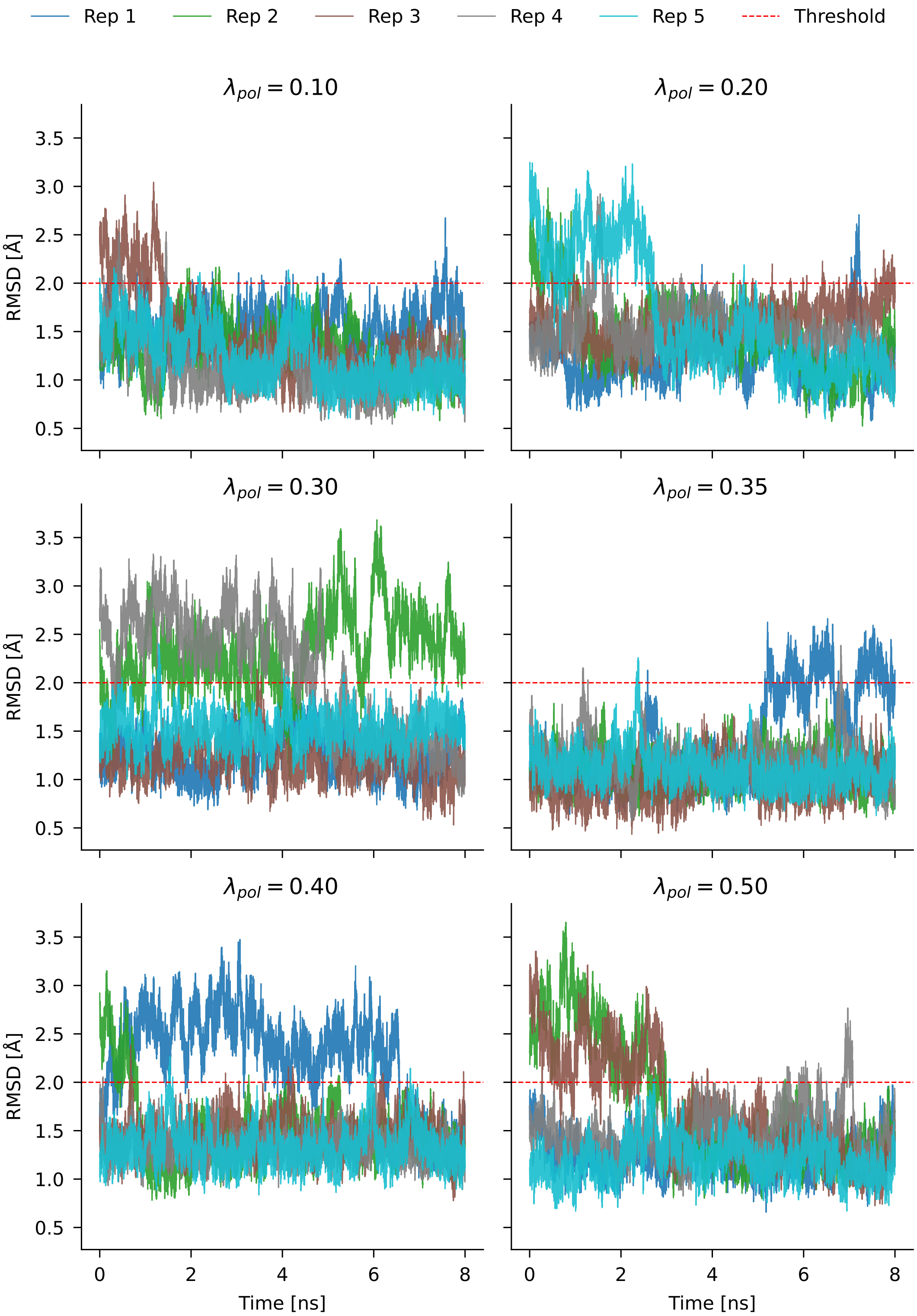

Figure S4.2: Time series of C_α-RMSD (five replicates) of Trp-cage in TIP4P-FB water for the polarization scaling scheme, with $\lambda_{\text{pol}} \in [0.1, 0.5]$.

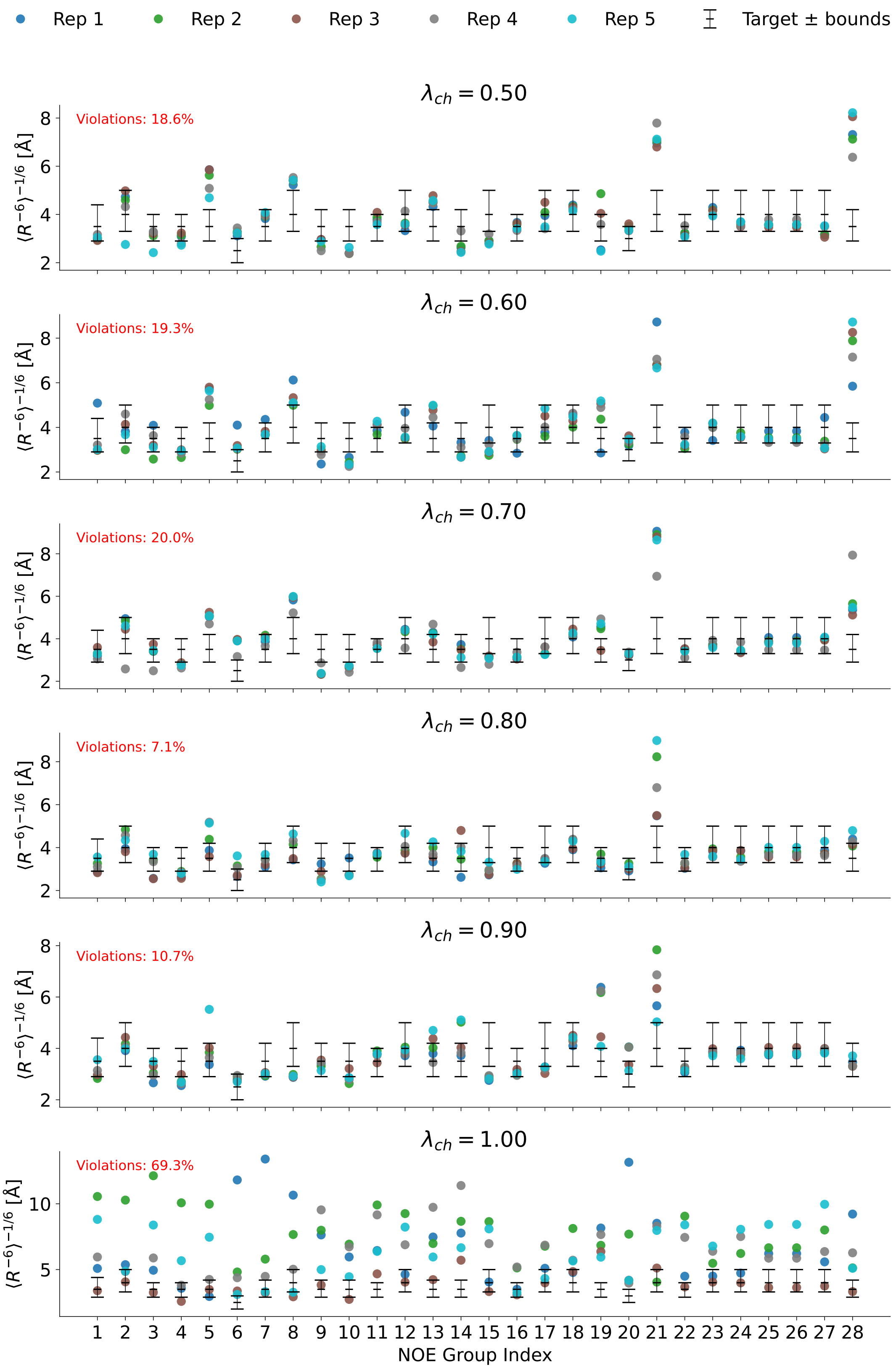

Figure S4.3: Average NOE distances for 28 pairs of protons in Trp-cage in TIP4P-FB water shown for five replicates. The charge scaling scheme was used with $\lambda_{ch} \in [0.5, 1.0]$.

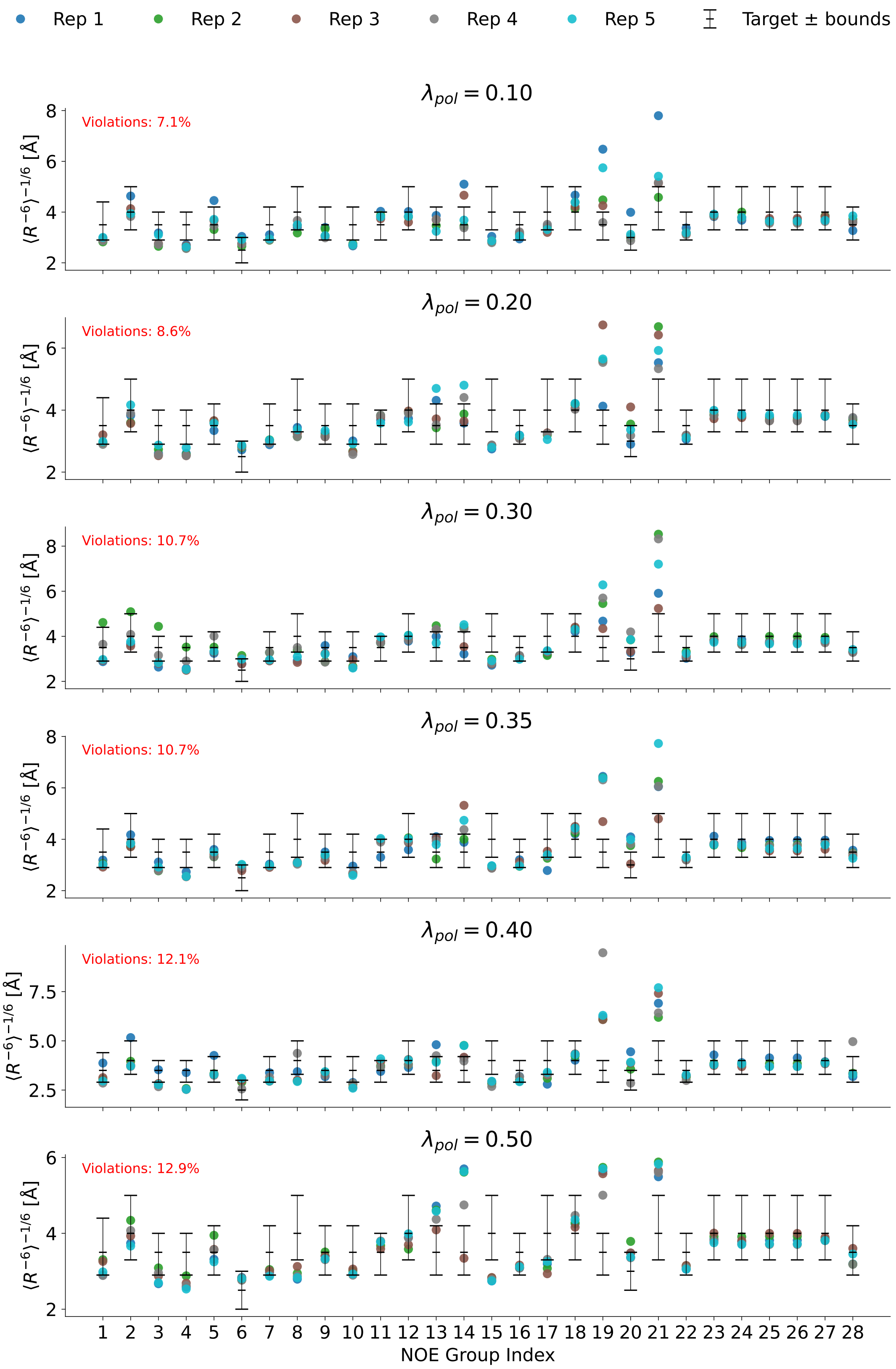

Figure S4.4: Average NOE distances for 28 pairs of protons in Trp-cage in TIP4P-FB water shown for five replicates. The polarization scaling scheme was used with $\lambda_{\mathrm{pol}} \in [0.1, 0.5]$.

S5 Additional Table for Gas-Phase Benchmarks

System	Atoms	Charge	Confs	MAE ΔEnergy [kJ·mol^{-1}]	MAE Gradients [kJ·mol^{-1}Å^{-1}]	MAE Dipole [e·Å]	MAE Quadrupole [e·Å^2]
ILE	31	0	50	1.71	1.53	0.03	0.06
HIP	30	1	50	1.71	3.49	0.09	0.14
CYS	23	0	50	2.42	2.09	0.03	0.09
THR	26	0	50	1.52	1.80	0.03	0.06
ASN	26	0	50	2.34	2.02	0.03	0.06
ALA	22	0	50	1.05	1.59	0.02	0.06
PHE	32	0	50	1.22	1.67	0.04	0.06
HID	29	0	50	1.88	2.05	0.03	0.09
SER	23	0	50	2.16	1.90	0.03	0.07
TRP	36	0	50	1.43	2.07	0.05	0.08
HIE	29	0	50	1.50	2.12	0.02	0.07
LEU	31	0	50	1.52	1.55	0.04	0.05
LYN	33	0	50	1.24	1.62	0.04	0.09
LYS	34	1	50	2.55	2.18	0.10	0.14
GLY	19	0	50	1.17	1.71	0.02	0.07
MET	29	0	50	2.65	1.81	0.04	0.08
GLN	29	0	50	2.97	1.80	0.03	0.06
ASP	24	-1	50	2.97	2.88	0.03	0.15
GLU	27	-1	50	1.64	2.72	0.04	0.23
Disulfide	44	0	50	2.71	1.95	0.05	0.15
GLH	28	0	50	1.95	1.88	0.03	0.07
PRO	26	0	50	1.59	1.85	0.04	0.07
ARG	36	1	50	2.05	2.74	0.10	0.21
VAL	28	0	50	0.78	1.57	0.03	0.06
TYR	33	0	50	1.42	1.78	0.04	0.07
ASH	25	0	50	3.08	2.02	0.03	0.07
CYX	22	-1	50	2.78	3.45	0.05	0.24
**Total average**	-	-	-	1.93	2.07	0.04	0.10

Table S5.1: Error statistics of AMP-BMS with respect to the reference DFT method (ωB97M-D4/ma-def2-TZVPP) for the monopeptides in the BMS25 dataset in the gas phase. MAEs of the total potential energy (MAE ΔEnergy), gradients (MAE Gradients), dipole moments (MAE Dipole), and quadrupole moments (MAE Quadrupole) are reported.

S6 Additional Figure for Computational Complexity

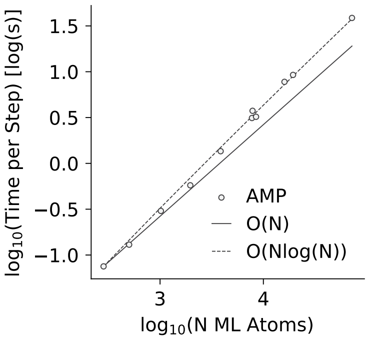

Figure S6.1: Log-log plot of time per inference step against number of ML atoms on CPU (AMD Ryzen 9 7950X, 196 GB system RAM). Scaling coefficient $k = 1.15$ according to $T(N) = CN^K$.

S7 Additional Figures for the Study of Protein Dynamics

S7.1 Time Series of Cα-RMSD

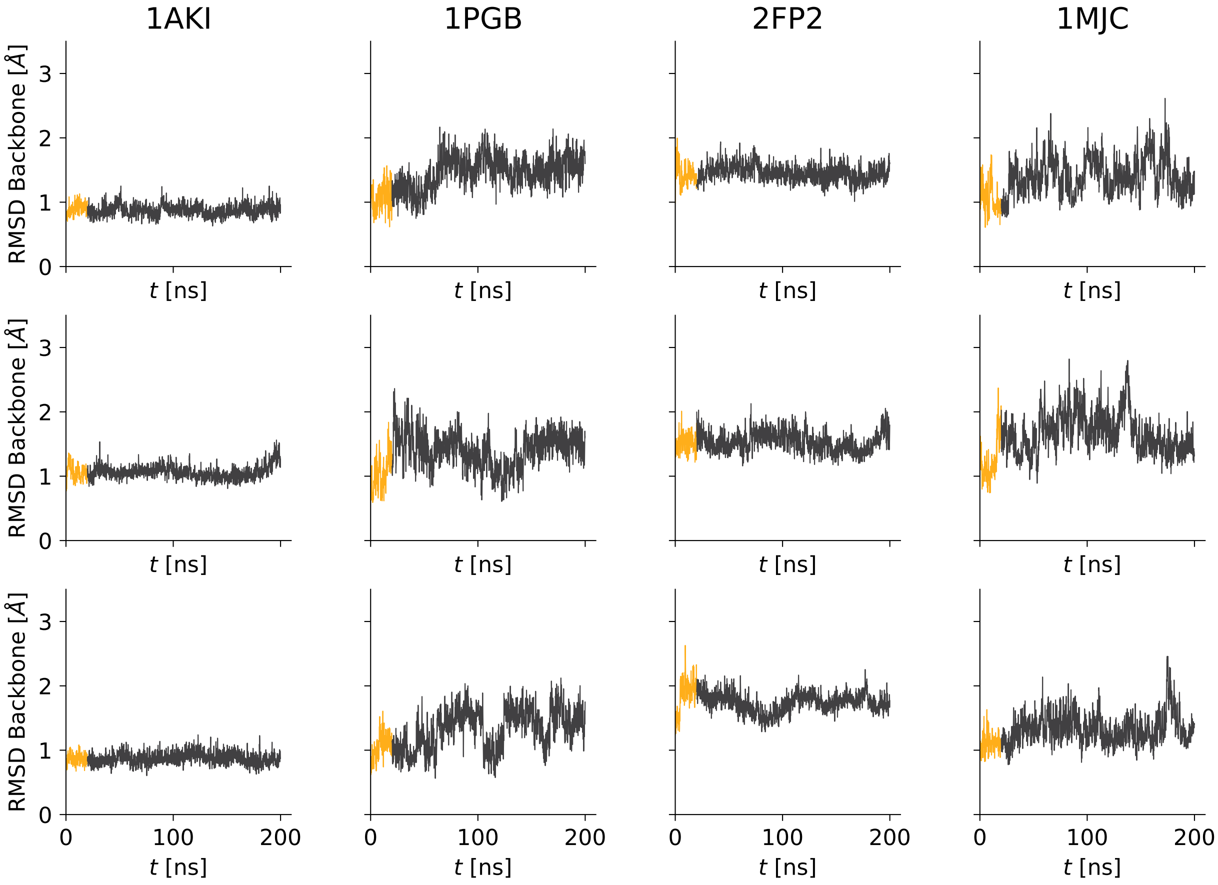

Figure S7.1: Times series of the Cα-RMSD with respect to the crystal structure for the four proteins simulated with the AMBER ff14SB force field [48]. Rows indicate replicates (different random number seed for the initial velocities). The first and last five residues were excluded from the RMSD calculation. The orange region marks the 20 ns equilibration phase.

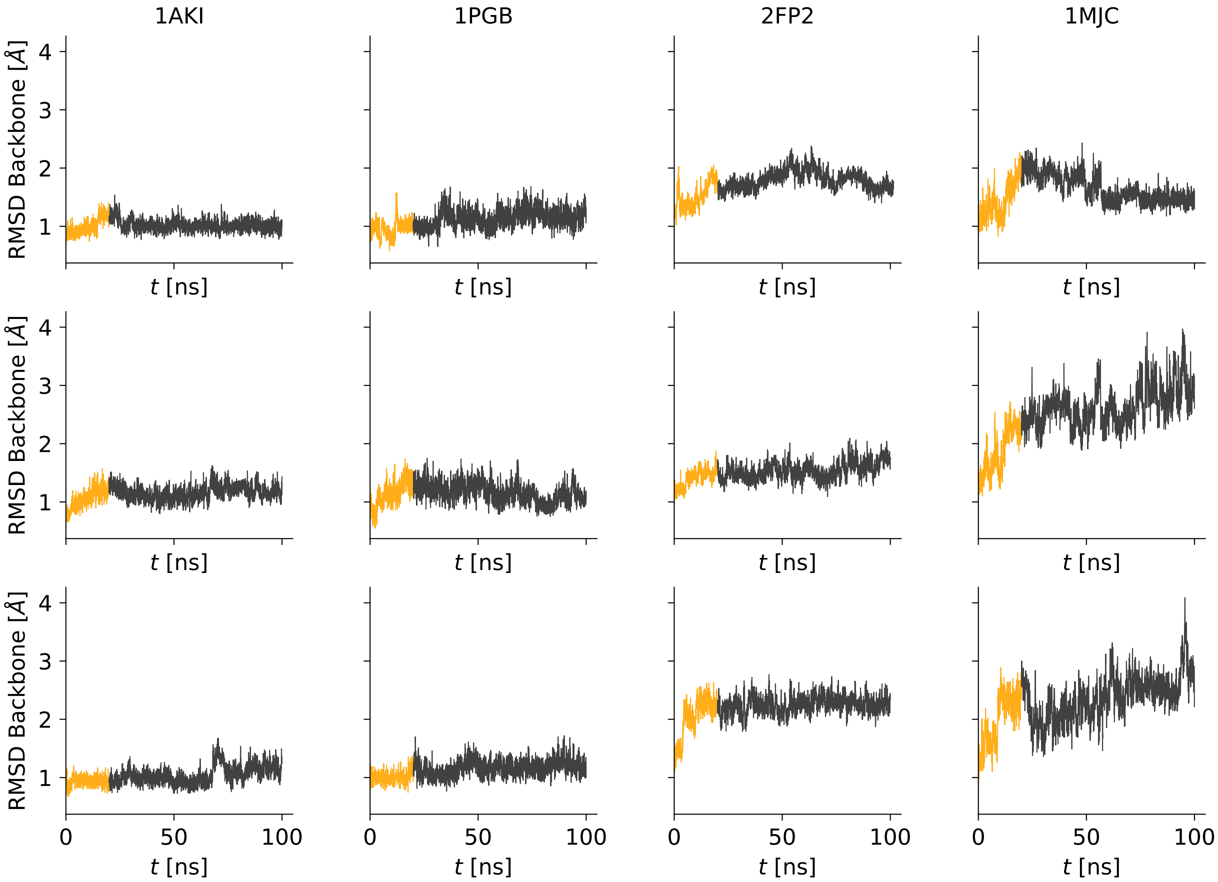

Figure S7.2: Times series of the Cα-RMSD with respect to the crystal structure for the four proteins simulated with AMP-BMS/MM using polarization scaling ($\lambda_{pol} = 0.35$). Rows indicate replicates (different random number seed for the initial velocities). The first and last five residues were excluded from the RMSD calculation. The orange region marks the 20 ns equilibration phase.

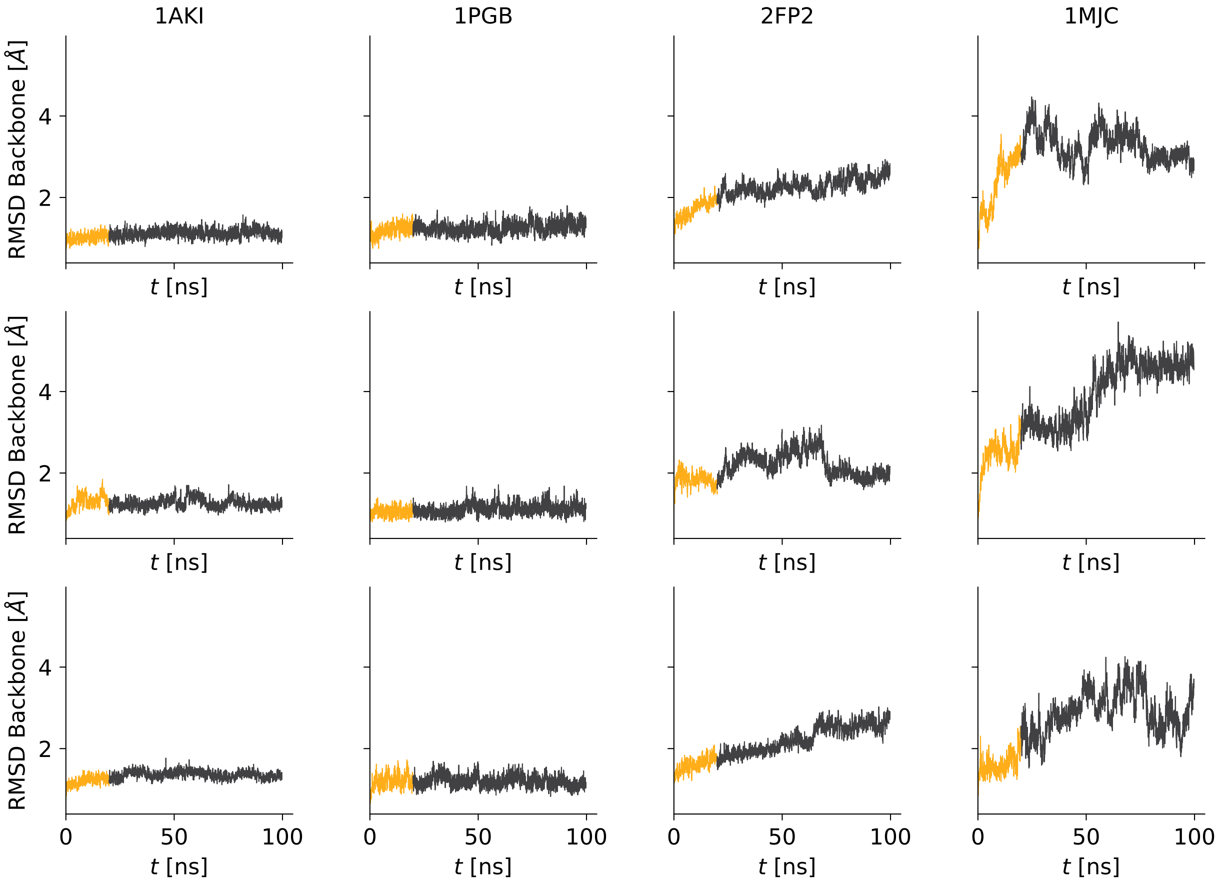

Figure S7.3: Times series of the Cα-RMSD with respect to the crystal structure for the four proteins simulated with AMP-BMS/MM using charge scaling (λ_{ch} = 0.9). Rows indicate replicates (different random number seed for the initial velocities). The first and last five residues were excluded from the RMSD calculation. The orange region marks the 20 ns equilibration phase.

S7.2 Time Series of Secondary Structure Motifs

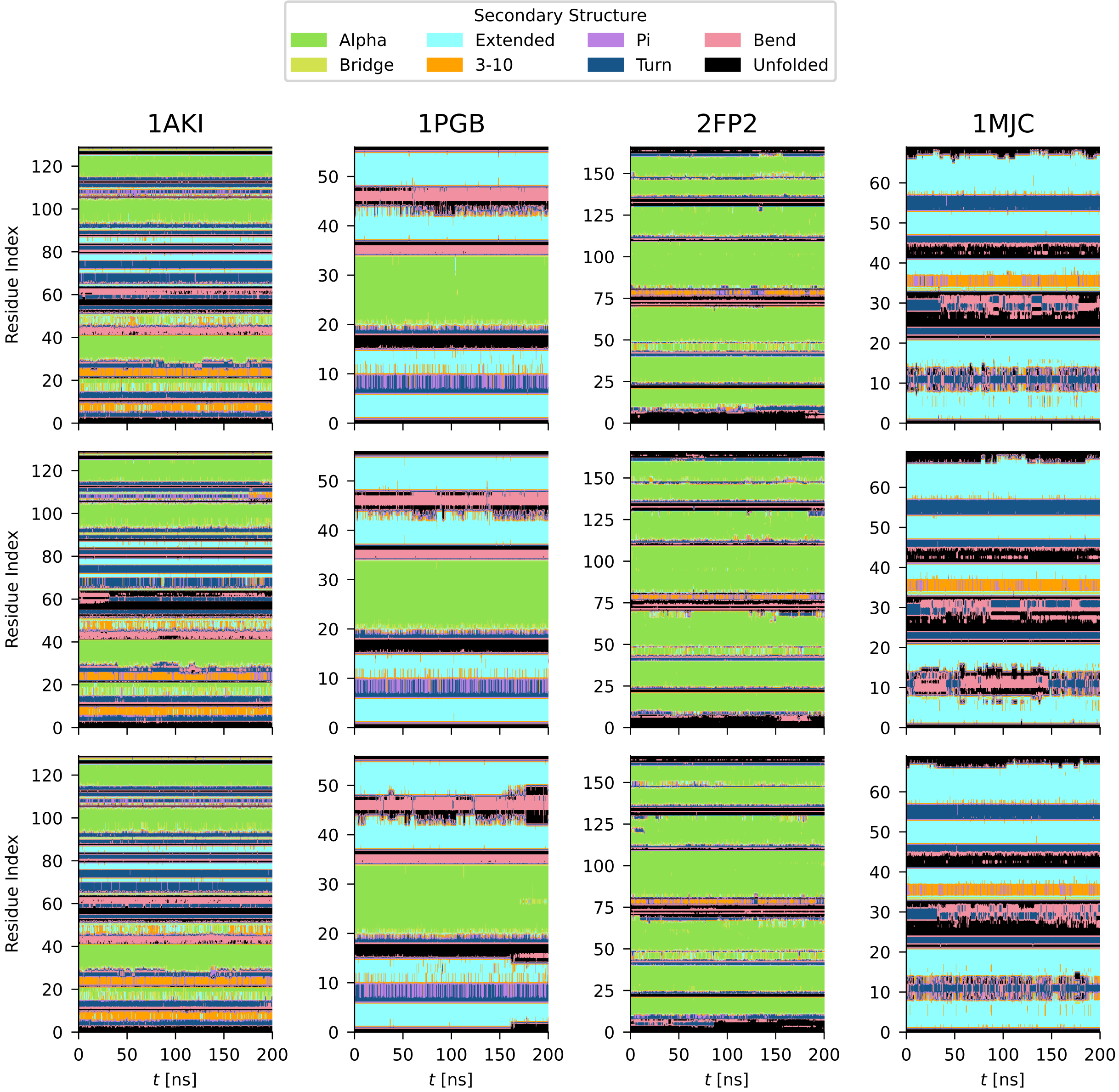

Figure S7.4: Time series of the secondary structure motifs of hen egg-white lysozyme (1AKI), major cold shock protein (1MJC), G-protein (1PGB), and chorismate mutase (2FP2) simulated with the AMBER ff14SB force field [48]. Rows indicate replicates (different random number seed for the initial velocities). The colors represent the secondary structure motifs: α-helix (green), β-bridge (light yellow), extended strand participating in β-ladder (cyan), 3_{10}-helix (orange), π-helix (purple), turns (dark blue), bends (pink), and unfolded regions (black).

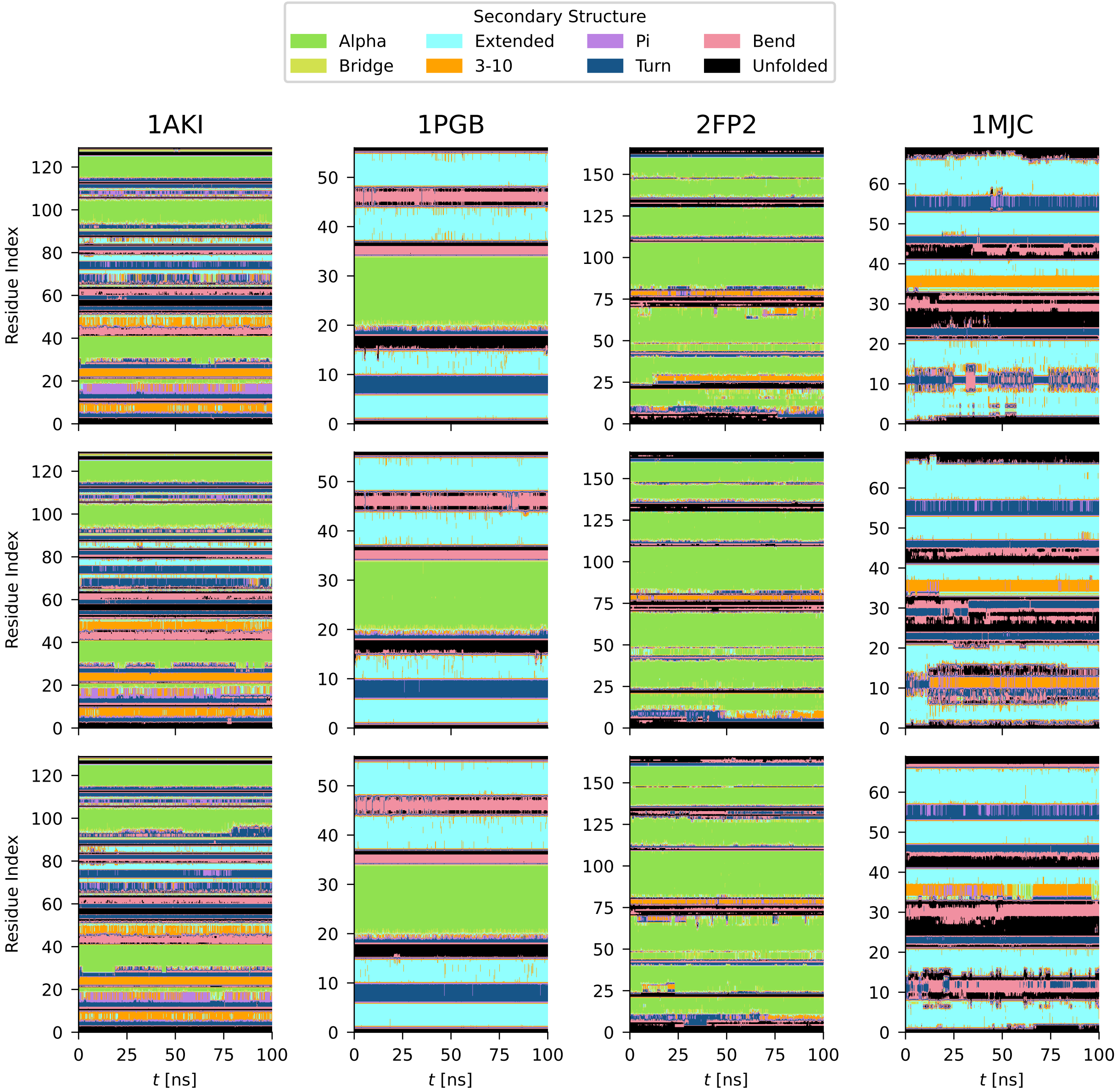

Figure S7.5: Time series of the secondary structure motifs of hen egg-white lysozyme (1AKI), major cold shock protein (1MJC), G-protein (1PGB), and chorismate mutase (2FP2) simulated with AMP-BMS/MM with polarization scaling ($\lambda_{\text{pol}} = 0.35$). Rows indicate replicates (different random number seed for the initial velocities). Same color code as in Figure S7.4.

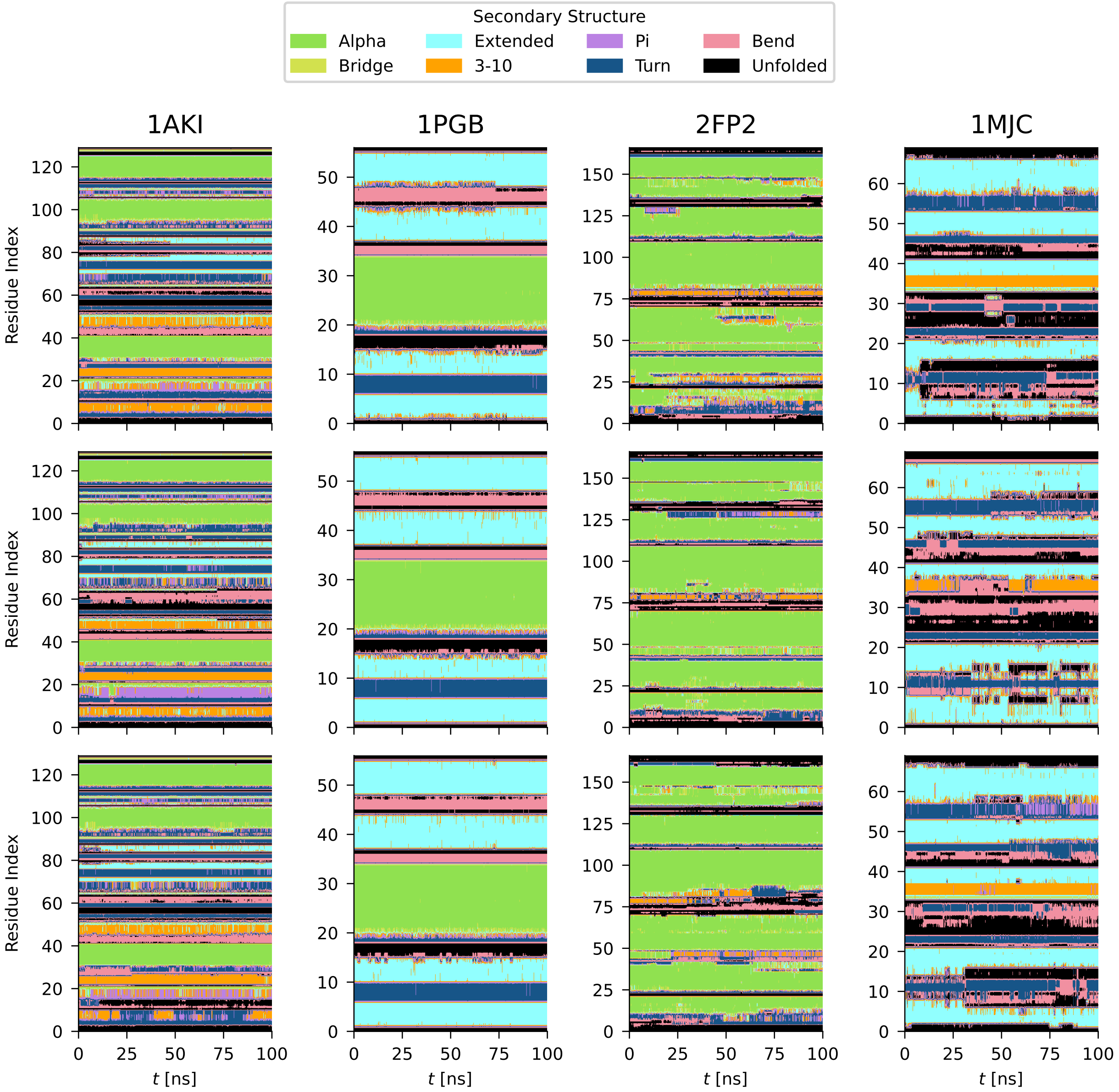

Figure S7.6: Time series of the secondary structure motifs of hen egg-white lysozyme (1AKI), major cold shock protein (1MJC), G-protein (1PGB), and chorismate mutase (2FP2) simulated with AMP-BMS/MM with charge scaling ($\lambda_{ch} = 0.9$). Rows indicate replicates (different random number seed for the initial velocities). Same color code as in Figure S7.4.

S7.3 NMR Comparison for HEWL

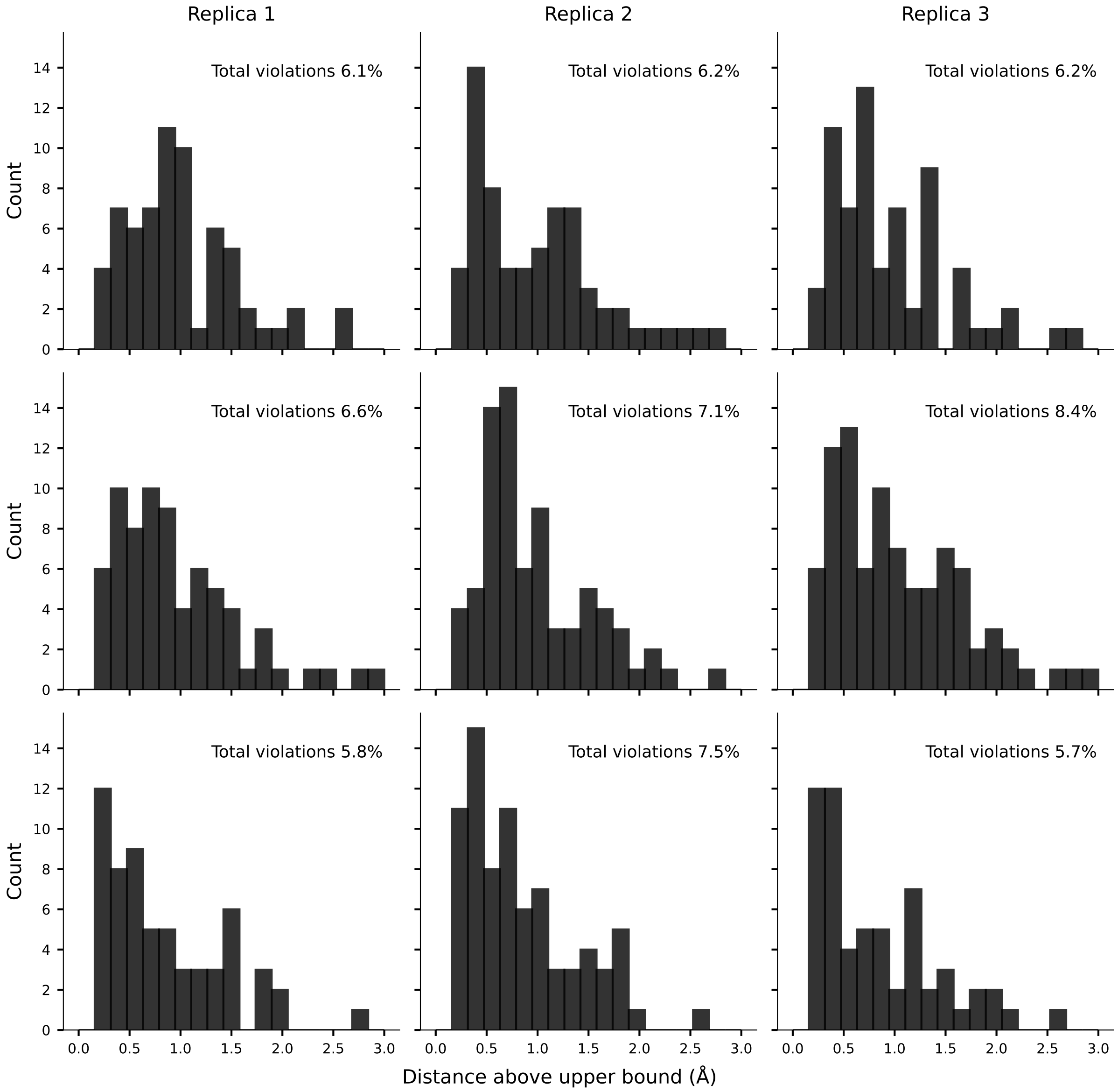

Figure S7.7: Distributions of NOE distance violations for hen egg-white lysozyme (HEWL) against experiment [49] for: AMP-BMS/MM with polarization scaling (top, $\lambda_{\mathrm{pol}} = 0.35$), AMP-BMS/MM with charge scaling (middle, $\lambda_{\mathrm{ch}} = 0.9$), and classical AMBER ff14SB force field [48] (bottom). Columns correspond to the replicates. A distance violation is defined as the excess of the ensemble-averaged interproton distance over the experimental upper bound. The percentage of violated restraints relative to the total number of NOE restraints is indicated in each subplot.

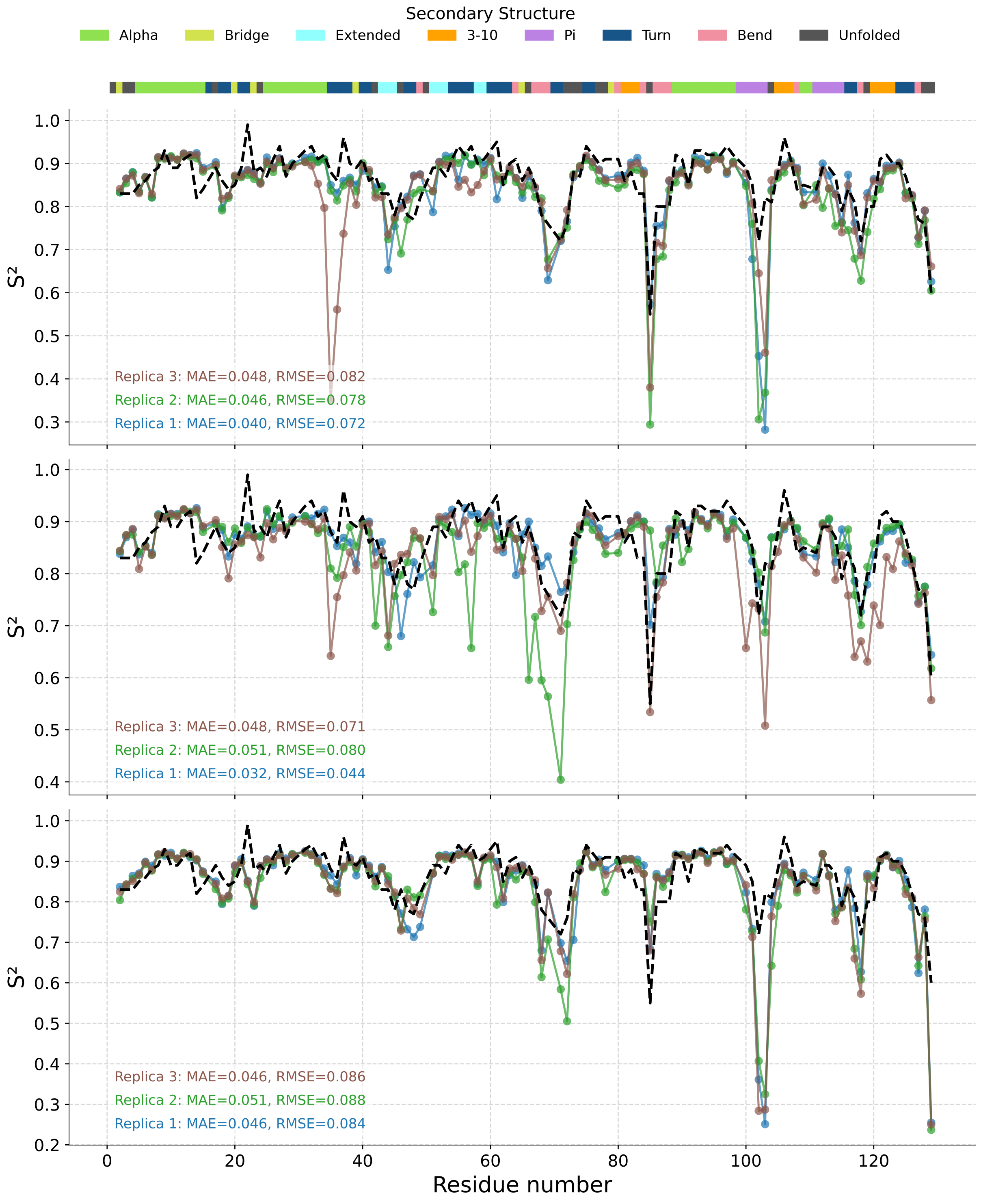

Figure S7.8: Comparison of backbone ^{1}H-^{15}N order parameters (S^2) of hen egg-white lysozyme (HEWL) from experiment [50] (dashed black line) and simulation: AMP-BMS/MM with polarization scaling (top, $\lambda_{\text{pol}} = 0.35$), AMP-BMS/MM with charge scaling (middle, $\lambda_{\text{ch}} = 0.9$), and classical AMBER ff14SB force field [48] (bottom). Colored curves represent the replicates. Values were calculated over the entire production runs (80 ns for AMP-BMS/MM; 180 ns for classical force field). Colored bars above each plot indicate the secondary structure motifs (same color code as in Figure S7.4).

S8 Additional Figures for the Study of Miniprotein Melting Curves

S8.1 Melting Curves

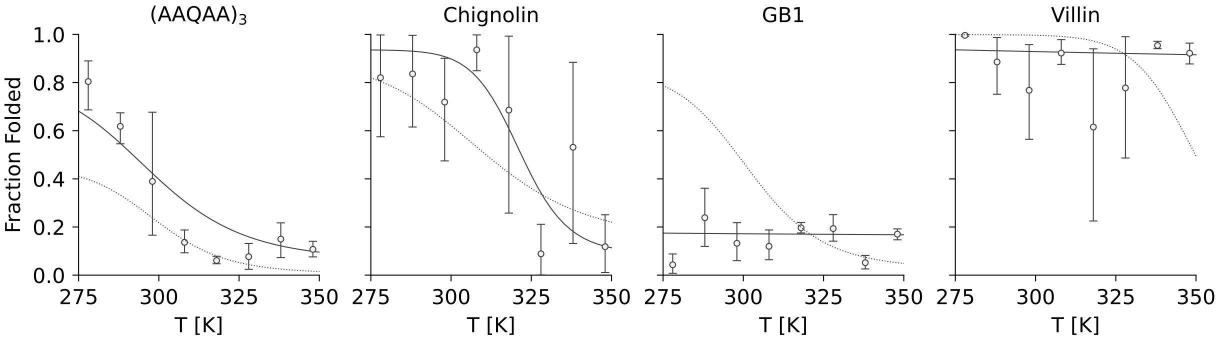

Figure S8.1: Melting curves for $(AAQAA)_3$, chignolin, GB1 hairpin and the villin headpiece subdomain obtained with the classical AMBER ff14SB force field [48] (black). Folded states were defined as the α-helix propensity in the case of $(AAQAA3)_3$ and the fraction of conformations below a RMSD threshold (see Methods). Dots indicate averages over three replicates of 50 ns length after 50 ns of equilibration, with error bars indicating the 10-90 percentile range. Data were fit to a two-state model (Eq. 34 in the main text) indicated by the black line. For comparison, the experimental melting curve is shown by the dashed line (details provided in the Methods section in the main text).

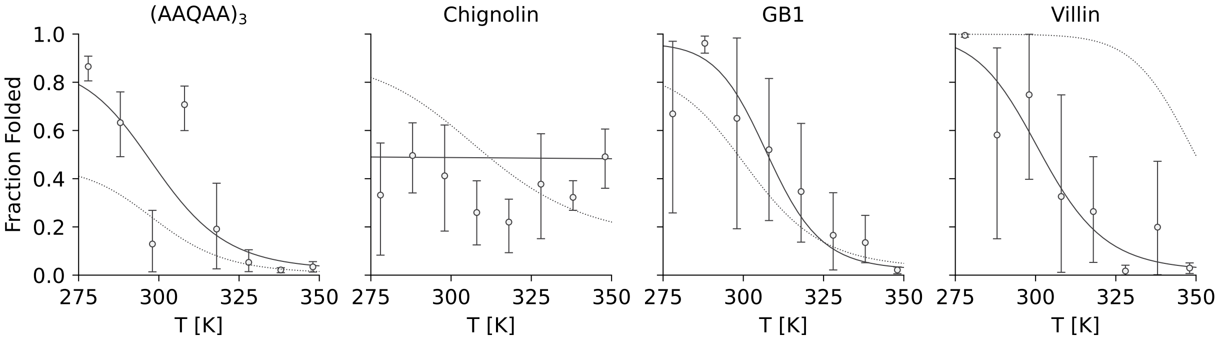

Figure S8.2: Melting curves for $(AAQAA)_3$, chignolin, GB1 hairpin and the villin headpiece subdomain obtained using AMP-BMS/MM with charge scaling (black, $\lambda_{\text{ch}} = 0.9$). Folded states were defined as the α-helix propensity in the case of $(AAQAA3)_3$ and the fraction of conformations below a RMSD threshold (see Methods). Dots indicate averages over three replicates of 50 ns length after 50 ns of equilibration, with error bars indicating the 10-90 percentile range. Data were fit to a two-state model (Eq. 34 in the main text) indicated by the black line. For comparison, the experimental melting curve is shown by the dashed line (details provided in the Methods section in the main text).

S8.2 Times Series of Cα-RMSD

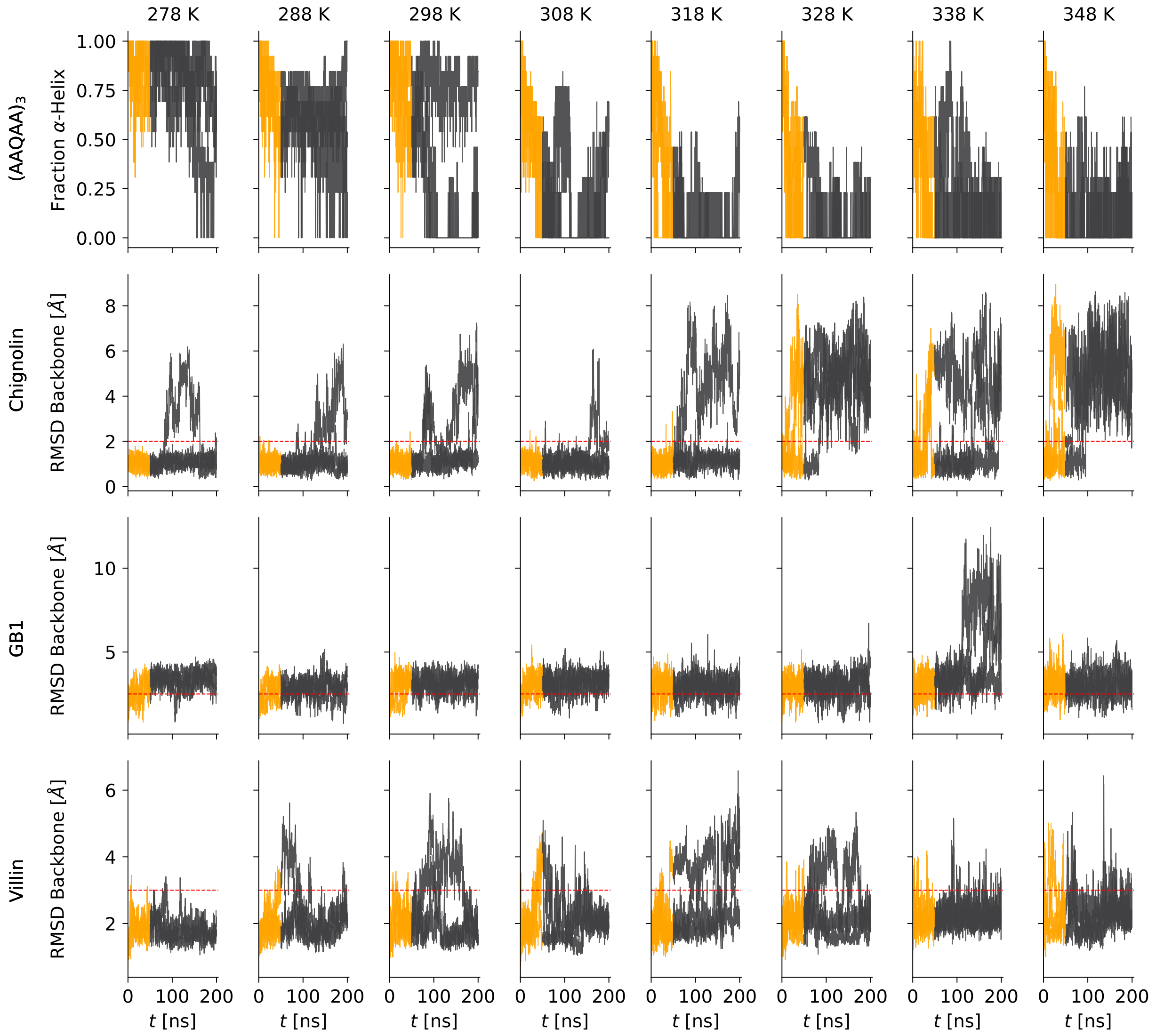

Figure S8.3: Times series of the Cα-RMSD with respect to the experimental structure for three miniproteins (chignolin, GB1, and villin headpiece subdomain) and the α-helix fraction of $(AAQAA)_3$ simulated with the classical AMBER ff14SB force field [48]. Each row corresponds to a protein, and each column corresponds to a temperature within the range $[278, 348]$ K. Three replicates are overlaid in each subplot. The orange shaded region indicates the 50 ns equilibration phase. The red dashed line signifies the cutoff used to determine the folded state.

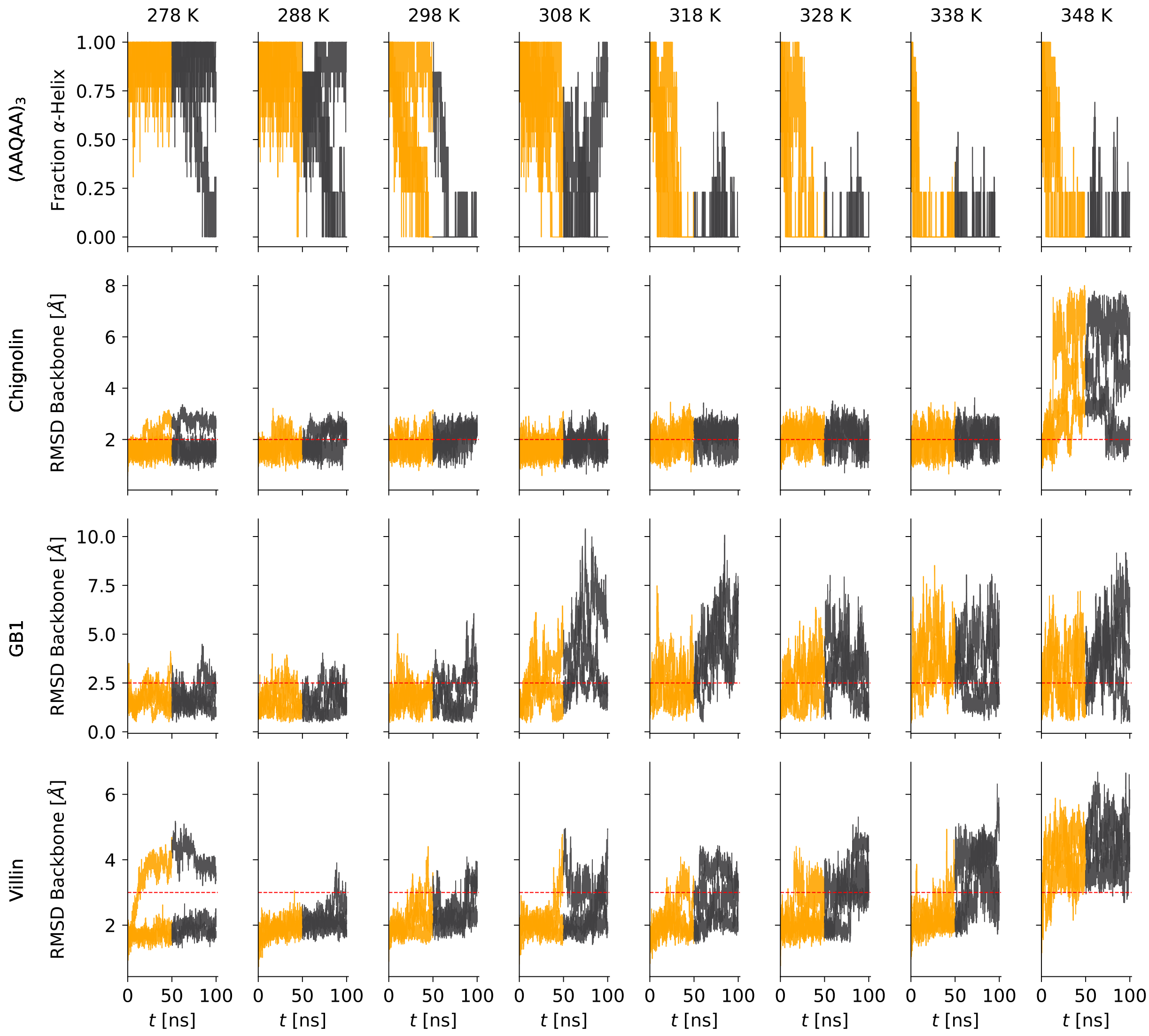

Figure S8.4: Times series of the Cα-RMSD with respect to the experimental structure for three miniproteins (chignolin, GB1, and villin headpiece subdomain) and the α-helix fraction of (AAQAA)$_3$ simulated with AMP-BMS/MM with polarization scaling ($\lambda_{\text{pol}} = 0.35$). Each row corresponds to a protein, and each column corresponds to a temperature within the range $[278, 348]$ K. Three replicates are overlaid in each subplot. The orange shaded region indicates the 50 ns equilibration phase. The red dashed line signifies the cutoff used to determine the folded state.

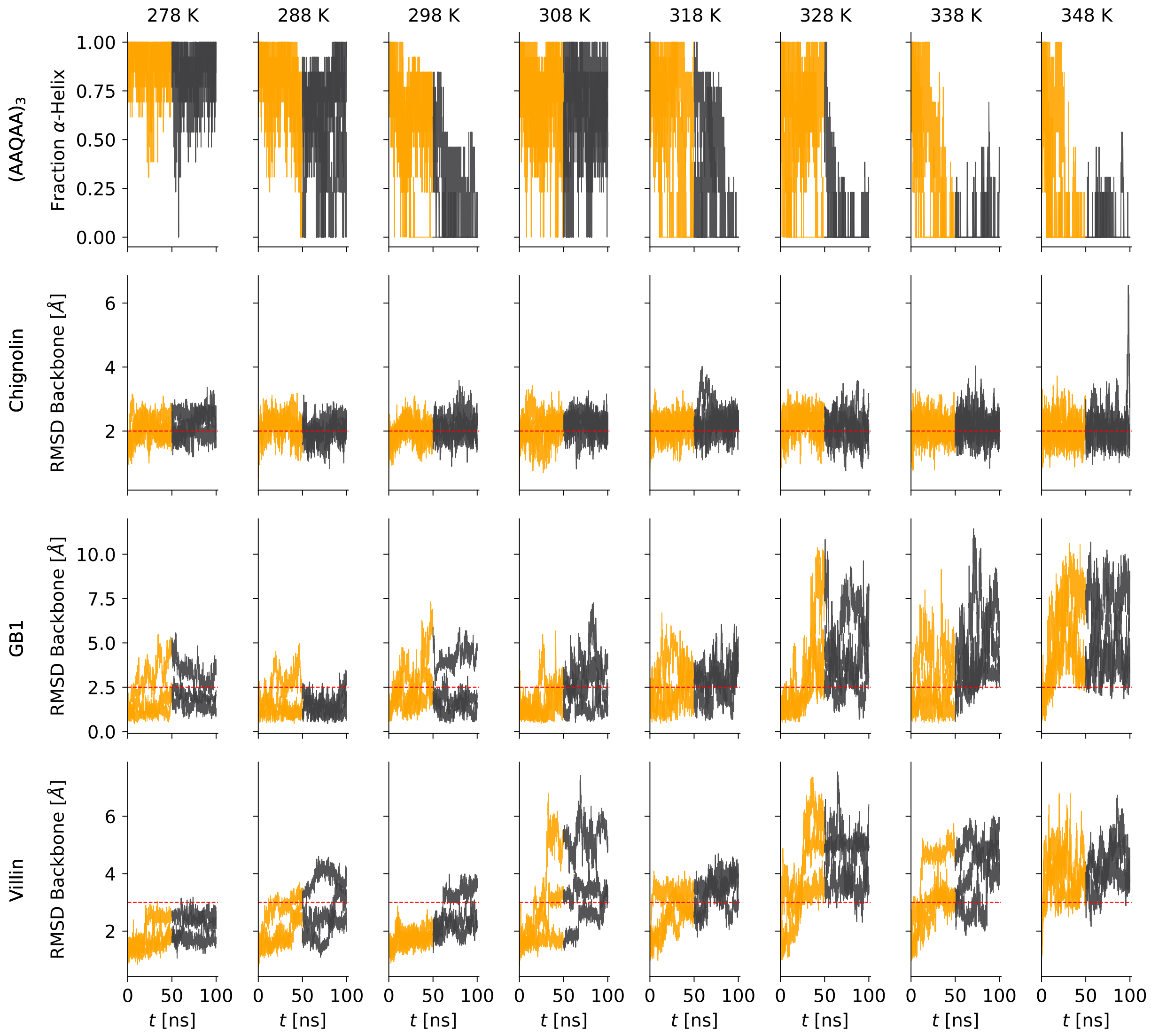

Figure S8.5: Times series of the Cα-RMSD with respect to the experimental structure for three miniproteins (chignolin, GB1, and villin headpiece subdomain) and the α-helix fraction of (AAQAA)$_3$ simulated with AMP-BMS/MM with charge scaling ($\lambda_{\text{ch}} = 0.9$). Each row corresponds to a protein, and each column corresponds to a temperature within the range $[278, 348]$ K. Three replicates are overlaid in each subplot. The orange shaded region indicates the 50 ns equilibration phase. The red dashed line signifies the cutoff used to determine the folded state.

S9 Additional Tables and Figures for the Study of Enzymatic Reactions

S9.1 Chorismate Mutase

Table S9.1: Umbrella definition for chorismate mutase free-energy calculations.

ξ_0 [Å]	k [kJ·mol^{-1}Å^{-2}]
-2.6	180
-2.4	180
-2.2	180
-2.0	180
-1.8	180
-1.6	180
-1.5	1800
-1.4	1800
-1.3	1800
-1.2	1800
-1.1	1800
-1.0	1800
-0.9	1800
-0.8	1800
-0.7	1800
-0.6	1800
-0.5	1800
-0.4	1800
-0.3	1800
-0.2	1800
-0.1	1800
0.0	1800
0.1	1800
0.2	1800
0.3	1800
0.4	1800
0.5	1800
0.6	1800
0.7	1800
0.8	1800
0.9	1800
1.0	1800
1.1	1800
1.2	1800
1.3	1800
1.4	1800
1.5	1800
1.6	180

Continued on next page

ξ_0 [Å]	k [kJ·mol^{-1}Å^{-2}]
1.8	180
2.0	180
2.2	180
2.4	180
2.6	180

System	ΔG [kJ mol^{-1}]	$\Delta G^{\dagger}$ [kJ mol^{-1}]	TS position [Å]
chorismate (aq.) (AMPv3-BMS25)	-55.2	191	-0.26
chorismate (aq.) (AMPv3-BMS25; FT ωB97M-D4) (aq.)	-54.0	144	-0.29
chorismate (aq.) (AMPv3-BMS25; FT B3LYP-D4) (aq.)	-53.2	103	-0.40
2CHT (AMPv3-BMS25)	-95.7	143	-0.17

Table S9.2: Free-energy difference, barrier height, and transition state (TS) position on the reaction coordinate ξ computed with default AMPv3-BMS25, fine-tuned AMPv3-BMS25 on ωB97M-D4/ma-def2-TZVPP level of theory, and fine-tuned AMPv3-BMS25 on B3LYP-D4/6-31G(d) level of theory for the isomerization of chorismate in aqueous (aq.) and enzyme (2CHT) environments (only with default model). Polarization scaling with $\lambda_{\text{pol}} = 0.35$ was used.

Literature	System	ΔG [kJ mol^{-1}]	$\Delta G^{\dagger}$ [kJ mol^{-1}]
Martí et al. [51]	chorismate (aq.)	-	159
Martí et al. [51]	2CHT	-	123
Crespo et al. [52]	chorismate (aq.)	-92.0	57.7
Crespo et al. [52]	2CHT	-128	18.0
Claeyssens et al. [53]	chorismate (aq.)	-69.9	72.8
Claeyssens et al. [53]	2CHT	-76.1	47.3
Kast et al. (exp.) [54, 55]	chorismate (aq.)	-56.0	103
Kast et al. (exp.) [54, 55]	2CHT	-56.0	64.4

Table S9.3: Comparison of free-energy differences and barrier heights for the isomerization of chorismate in aqueous and enzyme environments for previous studies.

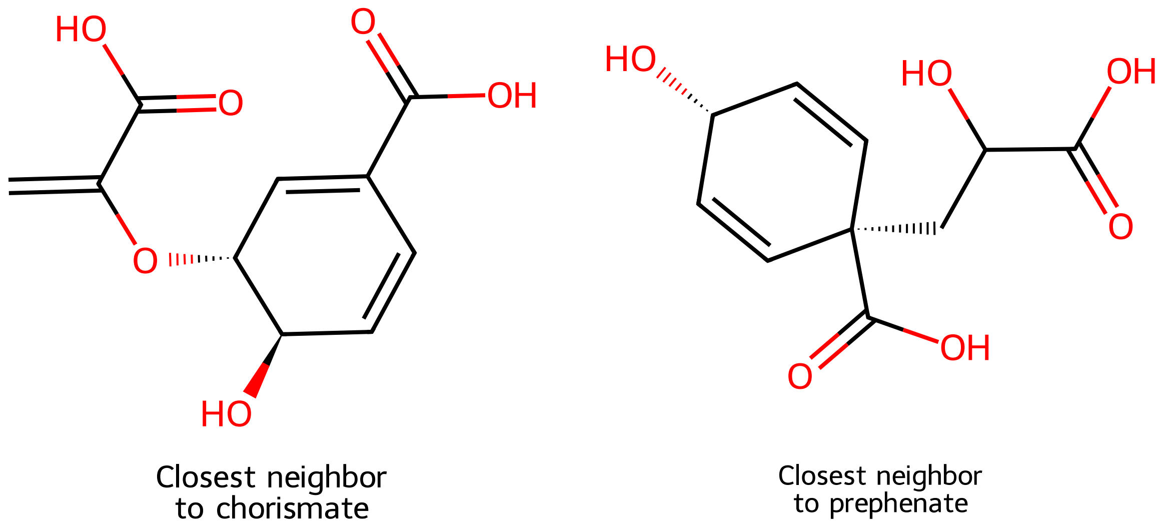

Figure S9.1: Most similar molecules to chorismate and prephenate in the BMS25 dataset [17] as measured by Tanimoto similarity of Morgan fingerprints (radius = 3, 8192 bits).

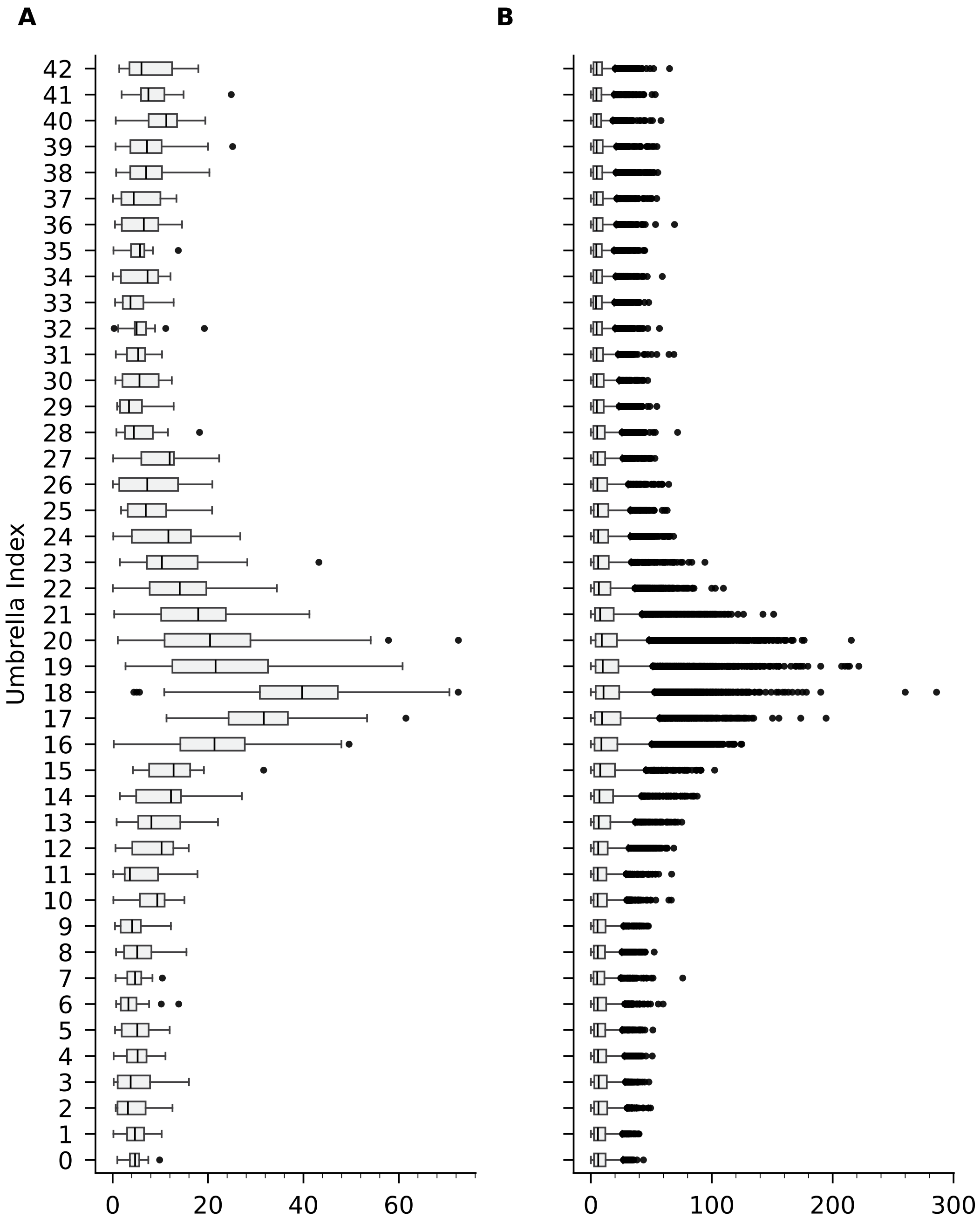

Figure S9.2: Distributions of absolute errors in energy (A) and force (B) for chorismate conformers from the validation subset of the fine-tuning dataset, resolved per umbrella window along the reaction coordinate. The underlying structures were sampled from a preliminary umbrella sampling simulation of the chorismate reaction in aqueous solution and span reactant-, transition state–, and product-like regions with non-uniform sampling density (see Methods section for details). Each box represents the distribution of absolute prediction errors within a single umbrella window, with outliers shown as individual points.

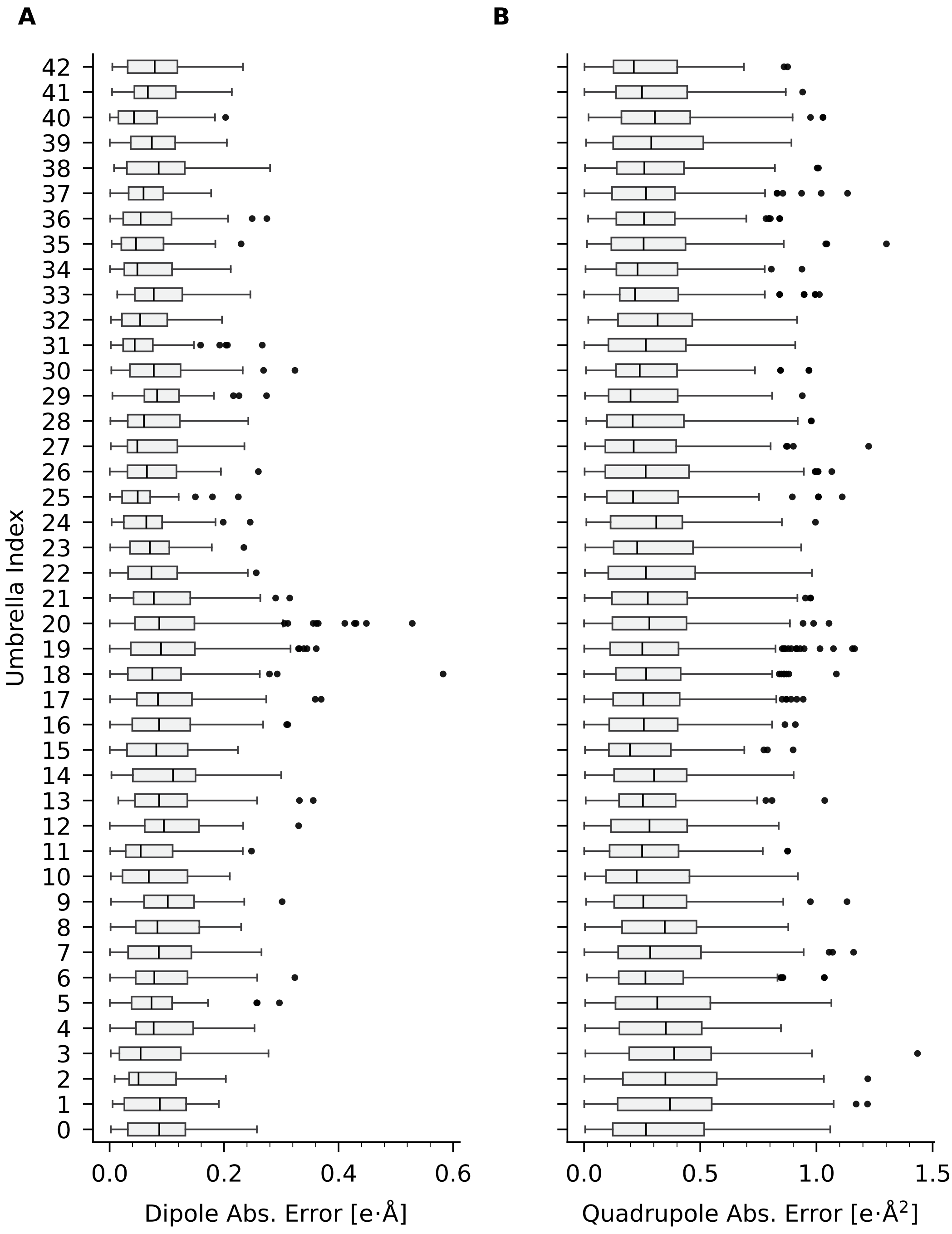

Figure S9.3: Distributions of absolute errors in dipole moment components (A) and quadrupole moment components (B) for chorismate conformers from the validation subset of the fine-tuning dataset, resolved per umbrella window along the reaction coordinate. The underlying structures were sampled from a preliminary umbrella sampling simulation of the chorismate reaction in aqueous solution and span reactant-, transition state–, and product-like regions with non-uniform sampling density (see Methods section for details). Each box represents the distribution of absolute prediction errors within a single umbrella window, with outliers shown as individual points.

Distance	Chorismate (MD) [Å]	Chorismate (QM) [Å]	Prephenate (MD) [Å]	Prephenate (QM) [Å]
C1-C6	3.92 (0.06)	4.21	1.55 (0.04)	1.53
O3-C4	1.43 (0.04)	1.43	4.13 (0.06)	-
C4-C5	1.50 (0.04)	1.50	1.32 (0.03)	1.32
C5-C6	1.33 (0.03)	1.33	1.50 (0.04)	1.50
C1-C2	1.33 (0.03)	1.32	1.51 (0.04)	1.50
C2-O3	1.35 (0.04)	1.38	1.21 (0.03)	1.21

Table S9.4: Selected distances of chorismate and prephenate in aqueous solution. MD refers to median distances in the AMP-BMS/MM simulations at the reactant and product stage on the reaction coordinate with interquartile range in parenthesis. QM refers to distances of DFT optimized reactant and product structures (ωB97M-D4/ma-def2-TZVPP with SMD solvation model [56].

S9.2 Fluoroacetate Dehalogenase

Table S9.5: Umbrella definition for fluoroacetate dehalogenase free-energy calculations.

ξ_0 [Å]	k [kJ·mol^{-1}Å^{-2}]
-3.0	150
-2.8	150
-2.6	150
-2.4	150
-2.2	150
-2.0	150
-1.8	150
-1.6	150
-1.5	1500
-1.4	1500
-1.3	1500
-1.2	1500
-1.1	1500
-1.0	1500
-0.9	1500
-0.8	1500
-0.7	1500
-0.6	1500
-0.5	1500
-0.4	1500
-0.3	1500
-0.2	1500
-0.1	1500
0.0	1500
0.1	1500
0.2	1500
0.3	1500

Continued on next page

ξ_0 [Å]	k [kJ·mol^{-1}Å^{-2}]
0.4	1500
0.5	1500
0.6	1500
0.7	1500
0.8	1500
0.9	1500
1.0	1500
1.1	1500
1.2	1500
1.3	1500
1.4	1500
1.5	1500
1.6	150
1.8	150
2.0	150
2.2	150
2.4	150
2.6	150
2.8	150
3.0	150

System	ΔG [kJ mol^{-1}]	$\Delta G^{\dagger}$ [kJ mol^{-1}]	TS position [Å]
WT	58.1	113	-0.39
mutant	82.0	143	-0.39

Table S9.6: Free-energy difference, barrier height, and transition state (TS) position on the reaction coordinate ξ computed with AMP-BMS/MM with polarization scaling ($\lambda_{\text{pol}} = 0.35$) for the rate-determining step of fluoroacetate degradation.

System	ΔG [kJ mol^{-1}]	$\Delta G^{\dagger}$ [kJ mol^{-1}]	TS position [Å]
WT	51.3	111	-0.39
mutant	81.2	140	-0.39

Table S9.7: Free-energy difference, barrier height, and transition state (TS) position on the reaction coordinate ξ computed with AMP-BMS/MM with charge scaling ($\lambda_{\text{ch}} = 0.9$) for the rate-determining step of fluoroacetate degradation.

Literature	System	$\Delta G^{\dagger}$ [kJ mol^{-1}]
This work	WT	113
This work	mutant	143
Kamachi et al. [57]	WT	94.1
Kamachi et al. [57]	mutant	-
Yue et al. [58]	WT	84.1
Yue et al. [58]	mutant	-
Chan et al. (exp.) [59]	WT	78.4
Chan et al. (exp.) [59]	mutant	-

Table S9.8: Comparison of free-energy differences and barrier heights for the rate-determining step of fluoroacetate degradation for this work and previous studies.

S10 Summary of Differences Between AMPv2 and AMPv3

Feature	AMPv2 [60]	AMPv3 (this work)
ML-ML Dispersion (D4)	✗	✓
ZBL Short range	✗	✓
ML-MM Calibration	✗	✓
Anisotropic ML-MM Polarization	✓	✗
ML-ML Electrostatics	Coulomb	Shifted reaction field
MD engine	GROMOS	OpenMM
Message Passing Steps	2/3	2
Cutoff Graph / Short Range (r_{SR})	5 Å	4 Å
Cutoff ML/ML Long Range ($r_{\mathrm{ML/ML,\,LR}}$)	14 Å	14 Å
Cutoff ML/MM Polarization ($r_{\mathrm{ML/MM,\,Pol}}$)	9 Å/10 Å	8 Å
Cutoff ML/MM LJ and Electrostatics ($r_{\mathrm{ML/MM,\,LR}}$)	14 Å	9 Å
Cutoff MM/MM Interaction ($r_{\mathrm{MM/MM}}$)	14 Å	9 Å
Node Size	128	128
Edge Size	32	32
Bessel Functions ML/ML	8/20	8
Bessel Functions ML/MM	8	2
Multipole Channels	32/64	8
Multipole Order	2	2
Model parameters	600’000 / 2’700’000	400’000

Table S10.1: Differences between previous work (AMPv2) [60] and the model (AMPv3) used in this work. For AMPv2, both values for the 600k and 2.7M models are reported if they differ.

S11 Errors for Molecular Multipoles

System (PDB ID)	# Atoms ML Zone	Total Charge	MAE Dipole [e Å]	MAE Quadrupole [e Å^2]
2RVD	166	-2	0.30 (0.0018)	0.89 (0.005)
2JOF	284	0	0.42 (0.0014)	2.15 (0.008)
1L2Y	304	1	0.74 (0.0024)	3.41 (0.011)
8TFV	351	6	0.60 (0.0017)	6.75 (0.019)
1M4F	361	4	0.71 (0.0020)	7.42 (0.021)
1CQ0	413	2	1.60 (0.0039)	6.26 (0.015)
1LFC	451	8	0.78 (0.0017)	8.95 (0.020)
1KGM	486	1	0.83 (0.0017)	5.75 (0.012)
1T5Q	487	-2	1.53 (0.0031)	3.91 (0.008)

Table S11.1: Performance of AMP-BMS/MM for the prediction of molecular multipoles for the miniproteins in the BMS25 dataset [17]: PDB identifier for each miniprotein, number of atoms in the ML zone, total charge, mean absolute error (MAE) with respect to the reference DFT method (ωB97M-D4/ma-def2-TZVPP) for the molecular dipole and the molecular quadrupole. Numbers in bracket show the MAE normalized by atom count. Errors for quadrupoles are calculated for the unique traceless components.

S12 Polarization Quality Benchmark

To assess how accurately the model reproduces the polarization of the ML region induced by MM point charges, the induced dipole moments in the ML region were analyzed. The reference induced dipole moment is defined as

$$\vec{\mu}_{\text{ind}}^{ref} = \vec{\mu}_{\text{embedded}}^{QM} - \vec{\mu}_{\text{gas}}^{QM} \tag{1}$$

where $\vec{\mu}\text{embedded}$ is the dipole moment of the QM region embedded in the electric field of the MM environment, and $\vec{\mu}\text{gas}$ is the dipole moment of the same QM region in vacuum. Similarly the ML-predicted induced dipole moment is defined as

$$\vec{\mu}_{\text{ind}}^{pred} = \vec{\mu}_{\text{embedded}}^{AMP} - \vec{\mu}_{\text{gas}}^{AMP} \tag{2}$$

To ensure that the results are easily interpretable, a neutral small molecule that is well represented in the training set was selected as the test system. In this way, any observed error in induction can be attributed primarily to the model itself rather than to deficiencies in the training data of the global model. Accordingly, an alanine amino acid capped with N-methyl and acetyl groups was chosen.

S12.1 Computational Details

The capped alanine molecules were solvated using the Modeller tool in OpenMM 8.1.2 [6] with 2.0 nm padding. Molecular dynamics simulations were performed using OpenMM 8.1.2 [5, 6]. Trajectories were sampled from the NPT ensemble using a Langevin middle integrator with a time step of $\Delta t = 2.0$ fs and a friction coefficient of $1/\text{ps}$ at 298.15 K [7]. A Monte Carlo barostat with a trial frequency of $n = 25$ was employed to maintain a target pressure of 1 bar [8]. The TIP4P-FB water model [9] was used with a cutoff of 1.0 nm. Bonds in the MM water molecules were constrained using the SETTLE algorithm [11] and hydrogen-containing bonds of alanine were constrained with SHAKE algorithm[61].

Long-range electrostatic interactions were computed using the Particle Mesh Ewald method [62]. Center-of-mass translation was removed at every step. Coordinates and energies were written every 0.5 ns. No counterions were added.

This procedure resulted in 200 snapshots. For each snapshot, three reference DFT calculations were performed: (i) a gas-phase calculation of the alanine system, in which all MM region charges from the solvent were ignored; (ii) a QM/MM electrostatic embedding calculation in which MM charges were included and the nearest QM–MM distance was within the short-range regime (defined such that the minimum QM–MM distance is within 5 Å); and (iii) a QM/MM electrostatic embedding calculation in which the MM charges were again included but restricted to a long-range regime (defined such that the minimum QM–MM distance lies between 5 and 8 Å). To better assess the directionality of the induced dipole moment and to avoid electric field cancellation near the center of the MM sphere, where the alanine molecule is located, only MM charges residing in the $(+,+,+)$ octant relative to the alanine center of mass were retained. This restriction ensures a non-isotropic solvent distribution around the QM region and allows for a more stringent test of the model's polarization response. In total, 600 QM/MM reference calculations were performed. All calculations were performed using the same level of theory employed in the BMS25 dataset (ωB97M-D4/ma-def2-TZVPP [23, 63–69]) with ORCA/5.0.4[28–32, 70].

S12.2 Results

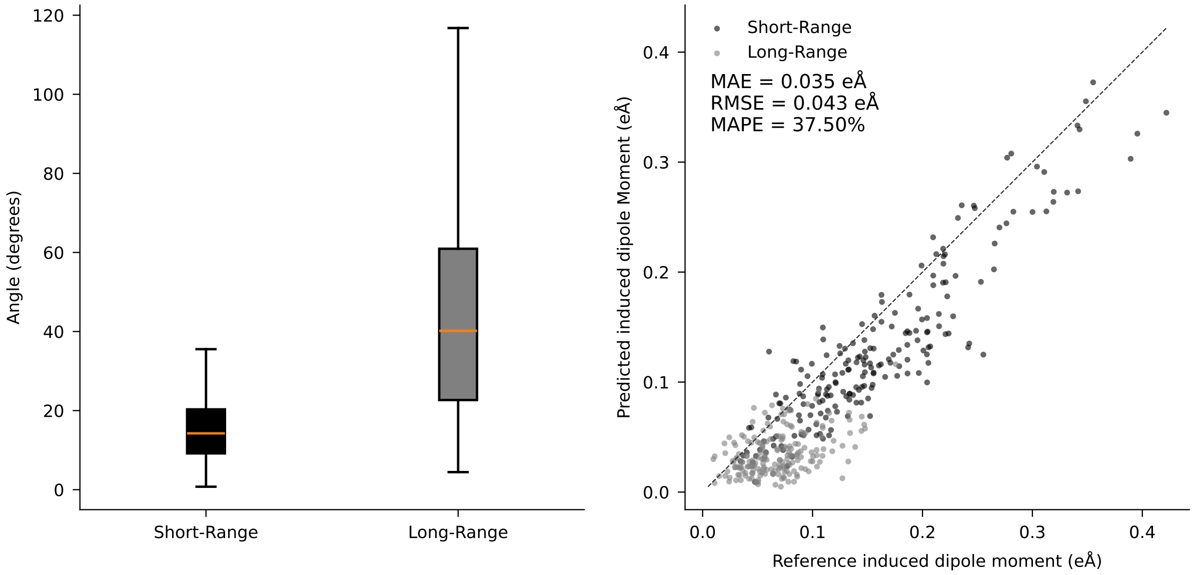

Figure S12.1: Performance of the AMPv3-BMS25 model in predicting induced dipole moments for short-range and long-range electric fields generated by MM zone point charges. (Left) Boxplots of the angular deviation (in degrees) between reference and predicted induced dipole moment vectors for short-range and long-range cases, illustrating the directional agreement. (Right) Correlation between reference and predicted magnitudes of the induced dipole moment (in eÅ) for short-range and long-range cases. The dashed line indicates perfect agreement. Error metrics for the combined dataset are reported: mean absolute error (MAE), root-mean-square error (RMSE), and mean absolute percentage error (MAPE).

The induced dipole moments predicted by the AMPv3-BMS25 model are in good overall agreement with the reference data, demonstrating that the model captures both the magnitude and direction of polarization effects with reasonable accuracy. Accurately reproducing both aspects is essential, as the physical validity of induction depends not only on the strength of the response but also on its orientation relative to the local electric field.

The left panel shows that short-range induction is significantly easier to model than long-range induction. The median angular deviation between predicted and reference dipole vectors is 14° for short-range, compared to 40° for long-range. This difference can be attributed to the weaker electric fields in the long-range regime, which result in smaller induced dipole magnitudes and, consequently, increased sensitivity of the orientation to small errors. Nevertheless, the model preserves the essential directionality of induction, indicating that it captures the underlying physics of polarization. In particular, this suggests that the relative atomic polarizabilities are predicted reasonably well, even though they were not explicitly included in the training objective.

The right panel shows that the predicted magnitudes of the induced dipole moments correlate well with reference values for short-range interactions, while the agreement deteriorates for long-range cases. This again reflects the difficulty of accurately predicting small induced moments. A systematic underestimation of the dipole magnitudes is observed, with an overall MAE of 0.035 eÅ and mean signed error of 0.031 eÅ. While this limitation will be addressed in future model developments, it is not critical in practice due to the empirical ML/MM calibration performed on experimental HFEs.

S13 Electrostatic Embedding Quality Benchmark

In Section S12, the quality of the environment-induced dipoles predicted by the AMP model was assessed. While this provides a good test of the induced electronic response, induced dipoles are not directly interpretable in energetic terms and therefore do not, on their own, establish whether the model reproduces the energetic effect of the MM environment on the ML region and on the ML/MM coupling. We adopt a diagnostic that is well defined and identically constructed in both the reference QM and the ML descriptions, namely the energetic stabilization of the QM or ML region, respectively, upon introduction of the surrounding MM point-charge field,

$$\Delta E \;=\; E^* - E, \tag{3}$$

where E^* denotes the energy of the region evaluated in the presence of the MM point charges and E denotes the energy of the same region (same coordinates) in vacuum. By further decomposing ΔE into an electrostatic-interaction and a polarization contribution (*vide infra*), we provide a deeper interpretation of λ_{pol}.

S13.1 Computational Details

All computations were carried out on 242 randomly selected data points taken from the SPICE subset [71] of the BMS25 dataset [17], using a single conformer per molecule. Each data point comprises a QM region together with its surrounding MM point charges. The systems were deliberately selected from the BMS25 dataset so that they lie within the applicability domain of the model. Consequently, the discrepancies reported below reflect intrinsic limitations of the model formulation rather than out-of-distribution data. For each data point, three single-point calculations were performed at the ωB97M-D4/ma-def2-TZVPP level of theory using the ORCA/6.0.1 [28–33] software package:

1. *Gas-phase reference.* A single-point calculation of the isolated QM region in the gas phase, i.e., with the MM point charges excluded. This provides the unpolarized electronic density and the corresponding gas-phase electronic energy.

2. *QM/MM electrostatic embedding.* A single-point calculation in which the MM point charges are included in the Hamiltonian, so that the QM density is polarized by the electrostatic field of the environment.

3. *Frozen embedded density in the gas phase.* A single-point calculation of the QM region in the gas phase (MM point charges removed) in which the converged QM/MM wavefunction from calculation 2 is supplied as the initial guess and no wavefunction relaxation is carried out (i.e., no self-consistent field iterations, the density is kept frozen). This evaluates the energy of the environment-polarized density in the absence of the external field.

S13.2 Theory

We denote by ρ_0 the gas-phase electron density obtained for the isolated QM region (calculation 1) and by ρ^* the density obtained in the presence of the MM point-charge field (calculation 2). The three reference calculations of Section S13.1 thus yield $E_{\text{gas}}[\rho_0]$ (calc. 1), $E_{\text{QM/MM}}[\rho^*]$ (calc. 2), and $E_{\text{gas}}[\rho^*]$ (calc. 3), the last being the field-polarized density evaluated with the MM charges switched off, obtained non-self-consistently from the converged QM/MM wavefunction.

The QM/MM *embedding energy* is defined as,

$$E_{\text{emb}}^{\text{QM}} = E_{\text{QM/MM}}[\rho^*] - E_{\text{gas}}[\rho_0], \tag{4}$$

which is precisely ΔE of Eq. 3 evaluated for the QM region. It may be partitioned into three physically distinct contributions,

$$E_{\text{emb}}^{\text{QM}} = E_1 + E_2 + E_3 \equiv E_{12} + E_3, \tag{5}$$

where

- E_1 is the electrostatic interaction of the unperturbed gas-phase charge distribution (electron density ρ_0 *and* nuclei) with the MM field – the *permanent* electrostatic interaction;
- E_2 is the electrostatic interaction of the induced density $\Delta\rho = \rho^* - \rho_0$ with the MM field – the *induced* electrostatic interaction;
- E_3 is the energetic cost of distorting the wavefunction from its gas-phase optimum to ρ^* – the *polarization energy*.

As the present three-calculation protocol does not allow for the separation of E_1 and E_2, we treat them together as the electrostatic-interaction energy of the fully polarized density, $E_{12} \equiv E_1 + E_2$.

The polarization energy is obtained directly from calculations 1 and 3,

$$E_3 = E_{\text{gas}}[\rho^*] - E_{\text{gas}}[\rho_0] \geq 0, \tag{6}$$

and the electrostatic-interaction energy is then recovered as the remainder of the embedding energy,

$$E_{12} = E_{\text{emb}}^{\text{QM}} - E_3 = E_{\text{QM/MM}}[\rho^*] - E_{\text{gas}}[\rho^*], \tag{7}$$

such that the decomposition is additive by construction. The polarization energy E_3 is non-negative because, by the variational principle, ρ_0 minimizes the gas-phase energy functional. Any other density, including the field-polarized ρ^*, therefore yields a higher gas-phase energy. E_3 thus represents the intrinsically destabilizing cost of polarization.

The identical decomposition can be constructed for the AMPv3-EE-BMS25 model. The combined permanent-plus-induced electrostatic interaction is encoded entirely in the ML/MM electrostatic coupling term, so that

$$E_{12}^{\mathrm{ML}} = V^{*}_{\mathrm{ES,ML/MM}}. \tag{8}$$

where the superscript $*$ denotes evaluation with the MM point charges *active* (field on) and its absence denotes the MM point charges *set to zero* (field off), in direct analogy with the starred notation of Eq. 3. The polarization energy E_3 is obtained as the change in the intramolecular ML energy upon activation of the MM field, i.e., the difference between the field-on and field-off values of node potential term V_{AMP} and the intramolecular electrostatics term $V_{\mathrm{ES,ML/ML}}$,

$$E_3^{\mathrm{ML}} = \left(V^{*}_{\mathrm{AMP}} + V^{*}_{\mathrm{ES,ML/ML}}\right) - \left(V_{\mathrm{AMP}} + V_{\mathrm{ES,ML/ML}}\right), \tag{9}$$

The ML embedding energy is then $E_{\mathrm{emb}}^{\mathrm{ML}} = E_{12}^{\mathrm{ML}} + E_3^{\mathrm{ML}}$. The accuracy of the model is assessed by comparing E_{12}^{ML} and E_3^{ML} against their QM reference counterparts E_{12} and E_3. For the subsequent analysis, we denote ML embedding energy evaluated with $\lambda = \lambda_{\mathrm{pol}}$ as $E_{\mathrm{emb}}^{\mathrm{ML}}(\lambda)$.

S13.3 Results

AMP-BMS MD simulations performed at $\lambda_{\mathrm{pol}} = 0.35$ are considerably more accurate than those at $\lambda_{\mathrm{pol}} = 1.0$, yielding improved absolute hydration free energies, protein stability, and water radial distribution functions with respect to experimental data. To rationalize this behavior, Figure S13.1 compares the predicted total embedding energy $E_{\mathrm{emb}}^{\mathrm{ML}}$ and its components E_{12}^{ML} and E_3^{ML} against their QM counterparts for both scaling factors. Importantly, neither of these two quantities was included in the model's training objective.

Both models reproduce the total embedding energy with high accuracy ($R^2 = 0.99$, MSE $= 0.6$ kJ mol^{-1} at $\lambda_{\mathrm{pol}} = 1.0$, and $R^2 = 0.98$, MSE $= 11.3$ kJ mol^{-1} at 0.35). The component analysis reveals, however, that the excellent total agreement of the $\lambda_{\mathrm{pol}} = 1.0$ model is the result of error cancellation: the systematic overestimation of E_{12} (mean signed error (MSE) $= +24.6$ kJ mol^{-1}) is nearly perfectly offset by the systematic underestimation of E_3 (MSE $= -24.0$ kJ mol^{-1}). These results are in good agreement with previously reported average 37.5% underprediction of induced dipole moments (Section S12). Despite this bias, E_{12} retains a good correlation ($R^2 = 0.97$), which is expected since E_{12} is dominated by the permanent electrostatic interaction E_1. By contrast, E_3 shows poor correlation with the QM reference ($R^2 = 0.14$, $\rho = 0.32$), confirming that the current model does not accurately describe the polarization cost, which is expected since it does not predict it as a separate quantity.

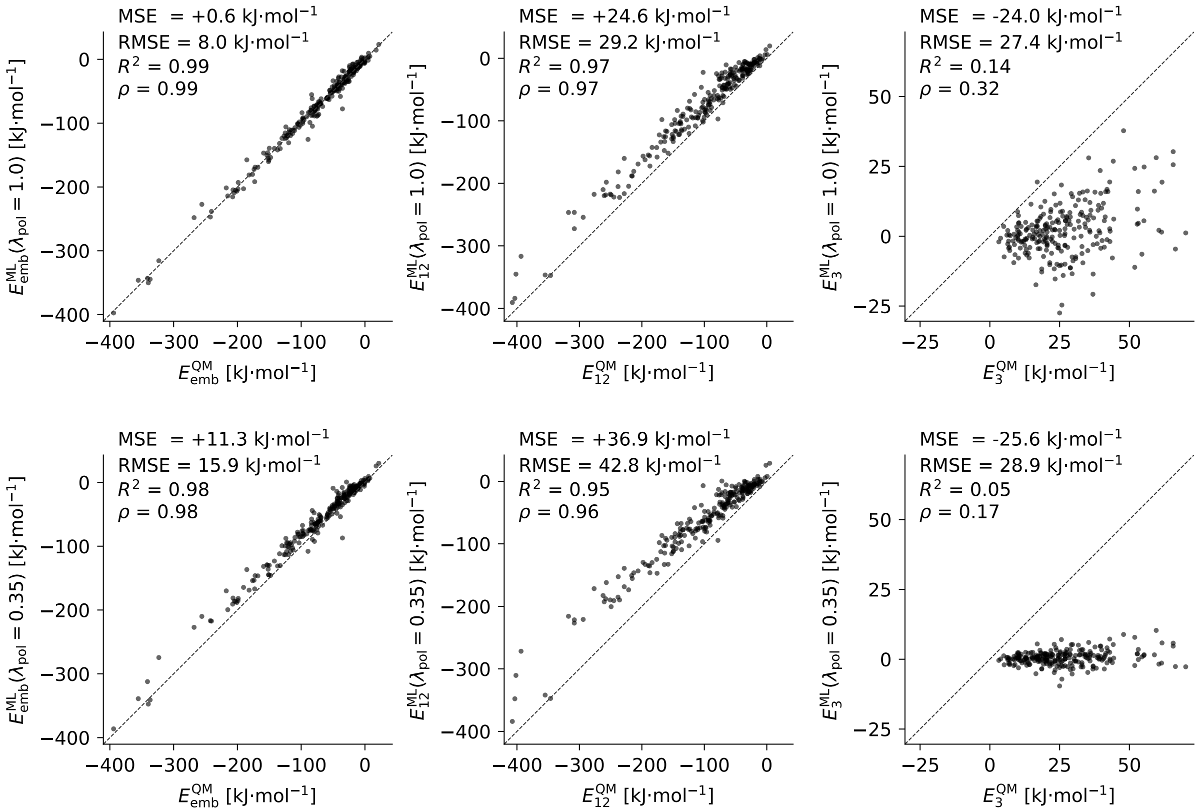

Figure S13.1: Component-resolved correlation of the predicted embedding energy for $\lambda_{\text{pol}} = 1.0$ (top row) and $\lambda_{\text{pol}} = 0.35$ (bottom row). Left to right: total embedding energy E_{emb}, electrostatic-interaction energy E_{12}, and polarization energy E_3. The dashed line indicates $y = x$. Metrics: mean signed error (MSE), root-mean-squared error (RMSE), R^2 (coefficient of determination), and ρ (Spearman rank correlation).

The effect of reducing λ_{pol} from 1.0 to 0.35 is asymmetric between the two components. The MSE of E_3 changes only marginally whereas the MSE of E_{12} increases considerably more (from $+24.6$ to $+36.9$ kJ mol^{-1}), reflecting the linear suppression of E_2 with λ. As a result, the $\lambda_{\text{pol}} = 0.35$ model effectively reduces to $E_{\text{emb}}^{\text{ML}} \approx E_{12}^{\text{ML}}$ – a systematic underestimation of the total interaction with no physically meaningful E_3 contribution, while retaining good correlation across studied systems for the total embedding energy. The systematic underestimation of E_{emb} relative to the DFT reference yielding substantially better results in the MD simulations constitutes direct evidence that the DFT embedding energy itself is too high due to the incompatibility of the QM Hamiltonian and the classical force field (i.e., too large fixed charges of non-polarizable MM water model). This aligns well with previously obtained results in this study. The empirical scaling corrects the net interaction energy with respect to experimental observables, but at the cost of physical interpretability of the individual components. This trade-off provides clear guidance for future model development.

From a conceptual standpoint, the default AMP-BMS model reproduces the total QM/MM interaction energy well, placing it closest in spirit to an electrostatic embedding scheme. The introduction of polarization scaling, while necessary to recover agreement with experimental observables, shifts the effective description toward a regime where the environmental polarization response is deliberately attenuated,

reminiscent of a mechanical embedding philosophy. This does not reflect a fundamental deficiency of the model, but rather an inherent consequence of compensating for the overpolarization introduced by fixed MM point charges in the absence of an explicit polarization model. Restoring the correct physical balance between embedding energy components without sacrificing experimental accuracy remains an open challenge.

References

[1] Mobley, D. L.; Guthrie, J. P. FreeSolv: a database of experimental and calculated hydration free energies, with input files. *J. Comput. Aided Mol. Des.* **2014**, *28*, 711–720.

[2] Weininger, D. SMILES, a chemical language and information system. 1. Introduction to methodology and encoding rules. *J. Chem. Inf. Comput. Sci.* **1988**, *28*, 31–36.

[3] Rogers, D.; Hahn, M. Extended-connectivity fingerprints. *J. Chem. Inf. Model.* **2010**, *50*, 742–754.

[4] Landrum, G. et al. rdkit/rdkit: 2024_03_3 (Q1 2024) Release. 2024.

[5] Eastman, P.; Swails, J.; Chodera, J. D.; McGibbon, R. T.; Zhao, Y.; Beauchamp, K. A.; Wang, L.-P.; Simmonett, A. C.; Harrigan, M. P.; Stern, C. D.; Wiewiora, R. P.; Brooks, B. R.; Pande, V. S. OpenMM 7: Rapid Development of High Performance Algorithms for Molecular Dynamics. *PLoS Comput. Biol.* **2017**, *13*, e1005659.

[6] Eastman, P. et al. OpenMM 8: Molecular Dynamics Simulation with Machine Learning Potentials. *J. Phys. Chem. B* **2024**, *128*, 109–116.

[7] Zhang, Z.; Liu, X.; Yan, K.; Tuckerman, M. E.; Liu, J. Unified Efficient Thermostat Scheme for the Canonical Ensemble with Holonomic or Isokinetic Constraints via Molecular Dynamics. *J. Phys. Chem. A* **2019**, *123*, 6056–6079.

[8] Chow, K.-H.; Ferguson, D. M. Isothermal-Isobaric Molecular Dynamics Simulations with Monte Carlo Volume Sampling. *Comput. Phys. Commun.* **1995**, *91*, 283–289.

[9] Wang, L.-P.; Martinez, T. J.; Pande, V. S. Building Force Fields: An Automatic, Systematic, and Reproducible Approach. *J. Phys. Chem. Lett.* **2014**, *5*, 1885–1891.

[10] Shirts, M. R.; Mobley, D. L.; Chodera, J. D.; Pande, V. S. Accurate and efficient corrections for missing dispersion interactions in molecular simulations. *J. Phys. Chem. B* **2007**, *111*, 13052–13063.

[11] Miyamoto, S.; Kollman, P. A. Settle: An analytical version of the SHAKE and RATTLE algorithm for rigid water models. *J. Comput. Chem.* **1992**, *13*, 952–962.

[12] Kubincová, A.; Riniker, S.; Hünenberger, P. H. Reaction-field electrostatics in molecular dynamics simulations: development of a conservative scheme compatible with an atomic cutoff. *Phys. Chem. Chem. Phys.* **2020**, *22*, 26419–26437.

[13] Boothroyd, S. et al. Development and Benchmarking of Open Force Field 2.0.0: The Sage Small Molecule Force Field. *J. Chem. Theory Comput.* **2023**, *19*, 3251–3275.

[14] Shirts, M. R.; Chodera, J. D. Statistically optimal analysis of samples from multiple equilibrium states. *J. Chem. Phys.* **2008**, *129*, 124105.

[15] Chodera, J. D.; Swope, W. C.; Pitera, J. W.; Seok, C.; Dill, K. A. Use of the weighted histogram analysis method for the analysis of simulated and parallel tempering simulations. *J. Chem. Theory Comput.* **2007**, *3*, 26–41.

[16] Chodera, J. D. A simple method for automated equilibration detection in molecular simulations. *J. Chem. Theory Comput.* **2016**, *12*, 1799–1805.

[17] Thürlemann, M.; Pultar, F.; Gordiy, I.; Riniker, S. Biomolecular Simulation (BMS) Dataset to Train Multiscale Neural Network Potentials. *ChemRxiv* **2025**, DOI: 10.26434/chemrxiv–2025–xzp6k.

[18] Hohenstein, E. G.; Sherrill, C. D. Density fitting of intramonomer correlation effects in symmetry-adapted perturbation theory. *J. Chem. Phys.* **2010**, *133*, 014101.

[19] Dunning Jr, T. H. Gaussian basis sets for use in correlated molecular calculations. I. The atoms boron through neon and hydrogen. *J. Chem. Phys.* **1989**, *90*, 1007–1023.

[20] Kendall, R. A.; Dunning Jr, T. H.; Harrison, R. J. Electron affinities of the first-row atoms revisited. Systematic basis sets and wave functions. *J. Chem. Phys.* **1992**, *96*, 6796–6806.

[21] Woon, D. E.; Dunning Jr, T. H. Gaussian basis sets for use in correlated molecular calculations. III. The atoms aluminum through argon. *J. Chem. Phys.* **1993**, *98*, 1358–1371.

[22] Pritchard, B. P.; Altarawy, D.; Didier, B.; Gibson, T. D.; Windus, T. L. New basis set exchange: An open, up-to-date resource for the molecular sciences community. *J. Chem. Inf. Model.* **2019**, *59*, 4814–4820.

[23] Mardirossian, N.; Head-Gordon, M. ωB97M-V: A combinatorially optimized, range-separated hybrid, meta-GGA density functional with VV10 nonlocal correlation. *J. Chem. Phys.* **2016**, *144*, 214110.

[24] Rappoport, D.; Furche, F. Property-optimized Gaussian basis sets for molecular response calculations. *J. Chem. Phys.* **2010**, *133*, 134105.

[25] Weigend, F.; Furche, F.; Ahlrichs, R. Gaussian basis sets of quadruple zeta valence quality for atoms H–Kr. *J. Chem. Phys.* **2003**, *119*, 12753–12762.

[26] Smith, D. G.; Burns, L. A.; Simmonett, A. C.; Parrish, R. M.; Schieber, M. C.; Galvelis, R.; Kraus, P.; Kruse, H.; Di Remigio, R.; Alenaizan, A., et al. Psi4 1.4: Open-source software for high-throughput quantum chemistry. *J. Chem. Phys.* **2020**, *152*, 184108.

[27] Lehtola, S.; Steigemann, C.; Oliveira, M. J.; Marques, M. A. Recent developments in libxc—A comprehensive library of functionals for density functional theory. *SoftwareX* **2018**, *7*, 1–5.

[28] Neese, F. Software Update: The ORCA Program System—Version 5.0. *Wiley Interdiscip. Rev.: Comput. Mol. Sci.* **2022**, *12*, e1606.

[29] Neese, F. An improvement of the resolution of the identity approximation for the formation of the Coulomb matrix. *J. Comput. Chem.* **2003**, *24*, 1740–1747.

[30] Neese, F.; Wennmohs, F.; Hansen, A.; Becker, U. Efficient, approximate and parallel Hartree–Fock and hybrid DFT calculations. A 'chain-of-spheres' algorithm for the Hartree–Fock exchange. *Chem. Phys.* **2009**, *356*, 98–109.

[31] Helmich-Paris, B.; de Souza, B.; Neese, F.; Izsák, R. An improved chain of spheres for exchange algorithm. *J. Chem. Phys.* **2021**, *155*.

[32] Neese, F. The SHARK integral generation and digestion system. *J. Comput. Chem.* **2023**, *44*, 381–396.

[33] Neese, F. Software update: The ORCA program system—version 6.0. *Wiley Interdiscip. Rev.: Comput. Mol. Sci.* **2025**, *15*, e70019.

[34] Eisenschitz, R.; London, F. About the relationship of the van der Waals forces to the covalent bonding forces. *Z. Phys.* **1930**, *60*, 491–527.

[35] Szalewicz, K.; Jeziorski, B. Symmetry-adapted double-perturbation analysis of intramolecular correlation effects in weak intermolecular interactions: the He-He interaction. *Mol. Phys.* **1979**, *38*, 191–208.

[36] Rybak, S.; Jeziorski, B.; Szalewicz, K. Many-body symmetry-adapted perturbation theory of intermolecular interactions. H2O and HF dimers. *J. Chem. Phys.* **1991**, *95*, 6576–6601.

[37] Patkowski, K. Recent developments in symmetry-adapted perturbation theory. *Wiley Interdiscip. Rev.: Comput. Mol. Sci.* **2020**, *10*, e1452.

[38] Parker, T. M.; Burns, L. A.; Parrish, R. M.; Ryno, A. G.; Sherrill, C. D. Levels of symmetry adapted perturbation theory (SAPT). I. Efficiency and performance for interaction energies. *J. Chem. Phys.* **2014**, *140*, 094106.

[39] Sorenson, J. M.; Hura, G.; Glaeser, R. M.; Head-Gordon, T. What can x-ray scattering tell us about the radial distribution functions of water? *J. Chem. Phys.* **2000**, *113*, 9149–9161.

[40] Berman, H. M.; Westbrook, J.; Feng, Z.; Gilliland, G.; Bhat, T. N.; Weissig, H.; Shindyalov, I. N.; Bourne, P. E. The Protein Data Bank. *Nucleic Acids Res.* **2000**, *28*, 235–242.

[41] Neidigh, J. W.; Fesinmeyer, R. M.; Andersen, N. H. Designing a 20-residue protein. *Nat. Struct. Mol. Biol.* **2002**, *9*, 425–430.

[42] Case, D. A.; Aktulga, H. M.; Belfon, K.; Cerutti, D. S.; Cisneros, G. A.; Cruzeiro, V. W. D.; Forouzesh, N.; Giese, T. J.; Gotz, A. W.; Gohlke, H., et al. AmberTools. *J. Chem. Inf. Model.* **2023**, *63*, 6183–6191.

[43] Schrödinger, LLC, The PyMOL Molecular Graphics System, Version 3.0.4. 2015.

[44] Zhou, R. Trp-cage: folding free energy landscape in explicit water. *Proc. Natl. Acad. Sci. U.S.A.* **2003**, *100*, 13280–13285.

[45] Hu, Z.; Tang, Y.; Wang, H.; Zhang, X.; Lei, M. Dynamics and cooperativity of Trp-cage folding. *Arch. Biochem. Biophys.* **2008**, *475*, 140–147.

[46] Marinelli, F.; Pietrucci, F.; Laio, A.; Piana, S. A kinetic model of trp-cage folding from multiple biased molecular dynamics simulations. *PLoS Comput. Biol.* **2009**, *5*, e1000452.

[47] Chowdhury, S.; Lee, M. C.; Duan, Y. Characterizing the rate-limiting step of Trp-cage folding by all-atom molecular dynamics simulations. *J. Phys. Chem. B* **2004**, *108*, 13855–13865.

[48] Maier, J. A.; Martinez, C.; Kasavajhala, K.; Wickstrom, L.; Hauser, K. E.; Simmerling, C. ff14SB: Improving the Accuracy of Protein Side Chain and Backbone Parameters from ff99SB. *J. Chem. Theory Comput.* **2015**, *11*, 3696–3713.

[49] Schwalbe, H.; Grimshaw, S. B.; Spencer, A.; Buck, M.; Boyd, J.; Dobson, C. M.; Redfield, C.; Smith, L. J. A refined solution structure of hen lysozyme determined using residual dipolar coupling data. *Protein Sci.* **2001**, *10*, 677–688.

[50] Buck, M.; Boyd, J.; Redfield, C.; Mackenzie, D. A.; Jeenes, D. J.; Archer, D. B.; Dobson, C. M. Structural determinants of protein dynamics: analysis of 15N NMR relaxation measurements for main-chain and side-chain nuclei of hen egg white lysozyme. *Biochemistry* **1995**, *34*, 4041–4055.

[51] Martí, S.; Andrés, J.; Moliner, V.; Silla, E.; Tuñón, I.; Bertrán, J.; Field, M. J. A Hybrid Potential Reaction Path and Free Energy Study of the Chorismate Mutase Reaction. *J. Am. Chem. Soc.* **2001**, *123*, 1709–1712.

[52] Crespo, A.; Scherlis, D. A.; Martí, M. A.; Ordejón, P.; Roitberg, A. E.; Estrin, D. A. A DFT-Based QM-MM Approach Designed for the Treatment of Large Molecular Systems: Application to Chorismate Mutase. *J. Phys. Chem. B* **2003**, *107*, 13728–13736.

[53] Claeyssens, F.; Ranaghan, K. E.; Lawan, N.; Macrae, S. J.; Manby, F. R.; Harvey, J. N.; Mulholland, A. J. Analysis of Chorismate Mutase Catalysis by QM/MM Modelling of Enzyme-Catalysed and Uncatalysed Reactions. *Org. Biomol. Chem.* **2011**, *9*, 1578.

[54] Kast, P.; Asif-Ullah, M.; Hilvert, D. Is Chorismate Mutase a Prototypic Entropy Trap? - Activation Parameters for the Bacillus Subtilis Enzyme. *Tet. Lett.* **1996**, *37*, 2691–2694.

[55] Kast, P.; Tewari, Y. B.; Wiest, O.; Hilvert, D.; Houk, K. N.; Goldberg, R. N. Thermodynamics of the Conversion of Chorismate to Prephenate: Experimental Results and Theoretical Predictions. *J. Phys. Chen. B* **1997**, *101*, 10976–10982.

[56] Marenich, A. V.; Cramer, C. J.; Truhlar, D. G. Universal solvation model based on solute electron density and on a continuum model of the solvent defined by the bulk dielectric constant and atomic surface tensions. *J. Phys. Chem. B* **2009**, *113*, 6378–6396.

[57] Kamachi, T.; Nakayama, T.; Shitamichi, O.; Jitsumori, K.; Kurihara, T.; Esaki, N.; Yoshizawa, K. The catalytic mechanism of fluoroacetate dehalogenase: a computational exploration of biological dehalogenation. *Chem. Eur. J.* **2009**, *15*, 7394–7403.

[58] Yue, Y.; Fan, J.; Xin, G.; Huang, Q.; Wang, J.-b.; Li, Y.; Zhang, Q.; Wang, W. Comprehensive Understanding of Fluoroacetate Dehalogenase-Catalyzed Degradation of Fluorocarboxylic Acids: A QM/MM Approach. *Environ. Sci. Technol.* **2021**, *55*, 9817–9825.

[59] Chan, P. W. Y.; Yakunin, A. F.; Edwards, E. A.; Pai, E. F. Mapping the Reaction Coordinates of Enzymatic Defluorination. *J. Am. Chem. Soc.* **2011**, *133*, 7461–7468.

[60] Pultar, F.; Thürlemann, M.; Gordiy, I.; Doloszeski, E.; Riniker, S. Neural Network Potential with Multiresolution Approach Enables Accurate Prediction of Reaction Free Energies in Solution. *J. Am. Chem. Soc.* **2025**, *147*, 6835–6856.

[61] Ryckaert, J.-P.; Ciccotti, G.; Berendsen, H. J. Numerical Integration of the Cartesian Equations of Motion of a System with Constraints: Molecular Dynamics of n-Alkanes. *J. Comput. Phys.* **1977**, *23*, 327–341.

[62] Darden, T.; York, D.; Pedersen, L. Particle Mesh Ewald: An N·log(N) Method for Ewald Sums in Large Systems. *J. Chem. Phys.* **1993**, *98*, 10089–10092.

[63] Johnson, E. R.; Becke, A. D. A Post-Hartree–Fock Model of Intermolecular Interactions. *J. Chem. Phys.* **2005**, *123*, 024101.

[64] Vydrov, O. A.; Van Voorhis, T. Nonlocal van der Waals density functional: The simpler the better. *J. Chem. Phys.* **2010**, *133*.

[65] Caldeweyher, E.; Ehlert, S.; Hansen, A.; Neugebauer, H.; Spicher, S.; Bannwarth, C.; Grimme, S. A generally applicable atomic-charge dependent London dispersion correction. *J. Chem. Phys.* **2019**, *150*, 154122.

[66] Caldeweyher, E.; Mewes, J.-M.; Ehlert, S.; Grimme, S. Extension and evaluation of the D4 London-dispersion model for periodic systems. *Phys. Chem. Chem. Phys.* **2020**, *22*, 8499–8512.

[67] Najibi, A.; Goerigk, L. DFT-D4 counterparts of leading meta-generalized-gradient approximation and hybrid density functionals for energetics and geometries. *J. Comp. Chem.* **2020**, *41*, 2562–2572.

[68] Weigend, F.; Ahlrichs, R. Balanced Basis Sets of Split Valence, Triple Zeta Valence and Quadruple Zeta Valence Quality for H to Rn: Design and Assessment of Accuracy. *Phys. Chem. Chem. Phys.* **2005**, *7*, 3297–3305.

[69] Zheng, J.; Xu, X.; Truhlar, D. G. Minimally Augmented Karlsruhe Basis Sets. *Theor. Chem. Acc.* **2011**, *128*, 295–305.

[70] Valeev, E. F. Libint: A Library for the Evaluation of Molecular Integrals of Many-Body Operators over Gaussian functions. 2022; version 2.8.0.

[71] Eastman, P.; Behara, P. K.; Dotson, D. L.; Galvelis, R.; Herr, J. E.; Horton, J. T.; Mao, Y.; Chodera, J. D.; Pritchard, B. P.; Wang, Y.; De Fabritiis, G.; Markland, T. E. SPICE, A Dataset of Drug-like Molecules and Peptides for Training Machine Learning Potentials. *Sci. Data* **2023**, *10*, 11.
